# Modalities and preferred routes of geographic spread of cholera from endemic areas in eastern Democratic Republic of the Congo

**DOI:** 10.1371/journal.pone.0263160

**Published:** 2022-02-07

**Authors:** Harry César Ntumba Kayembe, Didier Bompangue, Catherine Linard, Jérémie Muwonga, Michel Moutschen, Hippolyte Situakibanza, Pierre Ozer

**Affiliations:** 1 Département des Sciences de Base, Faculté de Médecine, Université de Kinshasa, Kinshasa, Democratic Republic of the Congo; 2 Département des Sciences et gestion de l’environnement, UR SPHERES, Faculté des Sciences, Université de Liège, Arlon, Belgium; 3 Chrono-Environnement, UMR CNRS 6249, Université de Franche-Comté, Besançon, France; 4 Département de Géographie, Université de Namur, Namur, Belgium; 5 Département de Biologie Médicale, Faculté de Médecine, Université de Kinshasa, Kinshasa, Democratic Republic of the Congo; 6 Département des Sciences cliniques, Immunopathologie–Maladies infectieuses et Médecine interne générale, Université de Liège, Liege, Belgium; 7 Département de Médecine interne, Faculté de Médecine, Université de Kinshasa, Kinshasa, Democratic Republic of the Congo; 8 Département de Parasitologie et Médecine tropicale, Faculté de Médecine, Université de Kinshasa, Kinshasa, Democratic Republic of the Congo; Institut National de la Recherche Agronomique, FRANCE

## Abstract

Cholera is endemic along the Great Lakes Region, in eastern Democratic Republic of the Congo (DRC). From these endemic areas, also under perpetual conflicts, outbreaks spread to other areas. However, the main routes of propagation remain unclear. This research aimed to explore the modalities and likely main routes of geographic spread of cholera from endemic areas in eastern DRC. We used historical reconstruction of major outbreak expansions of cholera since its introduction in eastern DRC, maps of distribution and spatiotemporal cluster detection analyses of cholera data from passive surveillance (2000–2017) to describe the spread dynamics of cholera from eastern DRC. Four modalities of geographic spread and their likely main routes from the source areas of epidemics to other areas were identified: in endemic eastern provinces, and in non-endemic provinces of eastern, central and western DRC. Using non-parametric statistics, we found that the higher the number of conflict events reported in eastern DRC, the greater the geographic spread of cholera across the country. The present study revealed that the dynamics of the spread of cholera follow a fairly well-defined spatial logic and can therefore be predicted.

## Introduction

Cholera is an epidemic-prone acute diarrheal disease caused by a well-recognized pathogen for humans “*Vibrio cholerae*” [1]. It is transmitted through ingestion of water or food contaminated with toxigenic forms of the bacterium [2]. Human to human contamination is also reported following limited access to clean drinking water and poor sanitation [2, 3].

The disease remains a worldwide major public health threat, especially in Sub-Saharan countries [4]. Large epidemics are annually recorded, particularly in the Democratic Republic of the Congo (DRC). According to World Health Organization (WHO), the DRC reported more than 420,000 suspected cholera cases during the 2000–2017 period. It accounted for nearly 10% and 17% of the global and African cholera-related morbidity, respectively [4].

Cholera was first reported in eastern DRC in 1978. Cases were determined to be imported from Kigoma in Tanzania to Kalemie, both bordering Lake Tanganyika, then the disease spread along the Great Lakes Region (GLR) [5, 6]. Over two decades later, the cholera control model in the DRC was essentially based on one-off response interventions in areas affected [5–7], and less attention has been paid to understanding the dynamics of the disease. Therefore, the role of certain factors, especially during inter-epidemic periods, in the recurrence of cholera outbreaks has not yet been investigated. Thus, the occurrence of epidemics seemed unpredictable, anarchic, even without spatio-temporal logic.

Since the 2000s, several epidemiological and ecological studies identified the GLR in eastern DRC as cholera endemic with stable transmission foci bordering lakes [8–10]. The role of seasonal population movements driven by fishing and commercial activities has been suggested in the spread of cholera outbreaks from hotspots to other areas [10, 11]. Furthermore, cholera outbreaks are infrequently reported in other parts of the country, particularly in western DRC. The latter is affected by the spatial spread of cholera from the GLR to the major cities upstream of the Congo River, before contiguously reaching the areas of the downstream provinces, and then the capital of Kinshasa and the mouth of the Congo River [12, 13]. During the last decade, this propagation pattern was observed in 2011–2012 and 2015–2017. A few phylogenetic analyses have recently confirmed the westward spread dynamics of cholera from the GLR [14, 15].

Despite the growing body of evidence on the spread of cholera in the DRC, the propagation routes, particularly those reaching areas outside the endemic eastern provinces, and their original epidemic foci remain unclear. We therefore hypothesized that there are several modalities and main routes of geographic spread of cholera from endemic lakeside areas.

The eastern DRC is also known as an active conflict zone for decades. War and conflict lead to massive population displacement, the collapse of health systems, and the breakdown of surveillance, early warning and response systems [16]. Conflict-affected populations are at risk of exposure to inadequate access to water, sanitation, and hygiene (WASH) facilities and resources [17, 18], which further increases the risk of epidemics [19]. While the impact of conflicts on the geographic spread of cholera was demonstrated elsewhere [20–22], epidemic reactivations of cholera in health zones (HZs) were suggested to be favored by conflicts in eastern DRC [9]. Thus, in our context, conflict events would amplify the spatial spread of cholera outbreaks out of endemic eastern provinces.

In this study, we aimed to explore the modalities and likely main routes of geographic spread of cholera from endemic areas in eastern DRC.

## Data and methods

### Study setting

The DRC is located in Central Africa. It has a total area of 2,345,000 km^2^ and a population of 86,895,208 inhabitants [23] with a population density estimated at 37.06 people/km^2^. Overall, the proportion of household population using an improved source of drinking water and improved sanitation facilities represents 59% and 32%, respectively [24].

**Fig 1:**
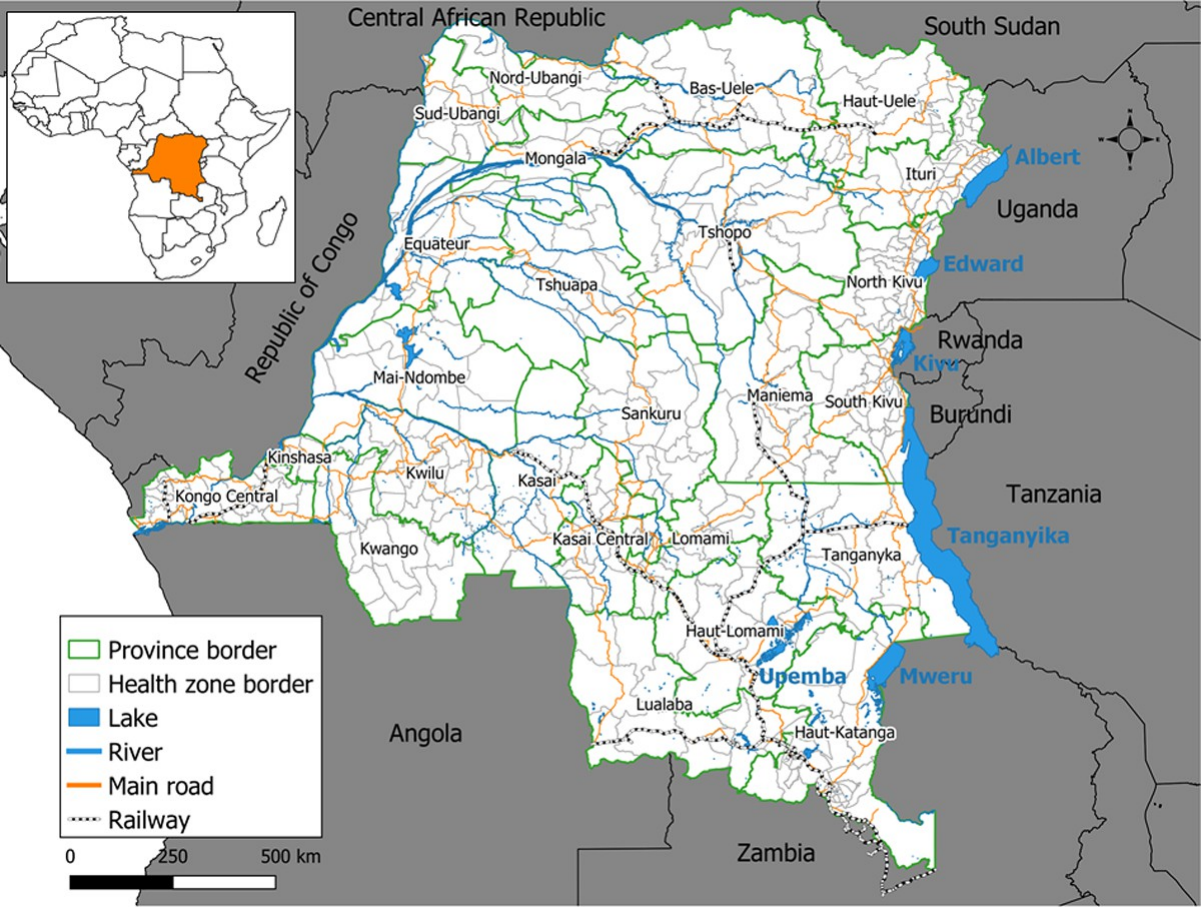
Administrative map of the DRC.

The country is currently subdivided into 26 administrative provinces also corresponding to provincial health divisions and 518 HZs (Fig 1). The HZ is the operational level of the health pyramid, while the provincial health division (intermediate level) play a technical and logistical role and the Minister of Health’s office and the general secretariat with 13 directorates and 52 specialized programs (central level) sets standards. Each HZ counts an average population of 100,000 to 300,000 inhabitants and consists of one reference general hospital and 15–20 health centers.

### Data collection

#### Information on major outbreak expansions of cholera documented since its introduction in eastern DRC

historical reconstruction was conducted to summarize major outbreak expansions of cholera since its introduction in eastern DRC in 1978 using queries on PubMed, Google and Google scholar. We used the following key words: “Cholera OR Vibrio cholerae” AND “Democratic Republic of the Congo”. Peer-reviewed and non-peer-reviewed articles published in English or French and reports from humanitarian agencies focused on the period 1970–2017 were searched. Only full texts that addressed the diffusion processes were considered.

#### Epidemiological surveillance data

Cholera is of 15 diseases with epidemic potential reported weekly by the National Integrated Disease Surveillance and Response system (IDSRS), established in 2000 through the partnership between the DRC Ministry of Health and WHO. Suspected cholera cases and deaths are identified through a syndromic approach. According to WHO, a suspected cholera case is defined as follows: “In areas where a cholera outbreak has not been declared: Any patient aged 2 years and older presenting with acute watery diarrhea and severe dehydration or dying from acute watery diarrhea. In areas where a cholera outbreak is declared: any person presenting with or dying from acute watery diarrhea” [25]. Each new outbreak is confirmed by culture and isolation of *Vibrio cholerae* O1 from stool samples [26].

Cholera morbidity and mortality data are recorded by medical officers in each cholera treatment center (CTC) using line lists [25] on hard format, and then aggregated electronically by Ministry of Health officials at HZ scale. These data are transmitted weekly to the provincial health divisions, before being centralized at the General Direction of Disease Control (https://dhis2.fbp-rdc.org/). The latter is the organ of the Ministry of Health that manages the IDSRS.

Here, we used weekly suspected cholera cases data collected at the HZ level from January 2000 to December 2017. Also, we defined cholera outbreaks as periods with the doubling of suspected cases in endemic HZs or the occurrence of at least one case in non-endemic HZs over three consecutive weeks (the end being respectively this 3-week period with the lowest threshold or no case reports).

#### Conflict data

Data on conflict events were derived from a leading publicly available conflict events dataset which provide high-resolution georeferenced and disaggregated data, the Armed Conflict Location and Event Dataset (ACLED). The ACLED reports both violent and non-violent events, without any restrictive fatalities threshold, at a daily step from cross-checking of multiple information sources [27]. This concerns multiple geographic scale (local, regional, national and continental) media, reports from non-governmental or international organizations in addition to media reporting, selected social media accounts (Twitter and Telegram) and partnerships with local conflict observatories in hard-to-access cases [27, 28]. Types of conflict events collected are: battles, explosions/remote violence, violence against civilians, protests, riots, and strategic developments [27]. In this study, we focused on conflict events reported during 2000–2017. They were aggregated at a weekly time step and the HZ scale.

#### GIS data

Free open shapefiles of large-scale boundaries of African countries [29] and the DRC at the HZ level [30] were obtained from the open access data platform “The Humanitarian Data Exchange”.

### Data analysis

Major outbreak expansions of cholera documented since its introduction in eastern DRC were summarized by cross-checking of information from published articles, epidemiological reports, and historical records of cholera outbreaks. We then mapped the propagation routes. Other maps of distribution of cholera cases per week and per HZ were generated to describe the geographic spread modalities of cholera from endemic areas in eastern DRC. All maps were produced using Quantum GIS version 3.8.3.

To explore the likely main routes of geographic spread of cholera outbreaks from endemic areas, the Kulldorff’s retrospective space-time permutation scan statistic was implemented to detect spatiotemporal clusters of annual cholera cases using SaTScan software version 9.6 [31]. This model does not require population-at-risk data, only the number of cases to estimate the expected number of cases. The scanning window is defined using a large number of overlapping cylinders which the circular base and the height represent respectively geographical location and time. Adjustments are made using a large number of random permutations of the spatial and temporal attributes of each observation in the dataset being studied. The most likely cluster is estimated for each permutation of the simulated dataset. Statistical significance is assessed using the Monte Carlo hypothesis testing [32]. In our study, the *p-value* was estimated using 999 Monte Carlo simulations. As Horwood et al. [33], after identifying very large spatiotemporal clusters containing a number of statistically significant sub-clusters in preliminary analyses, we set the maximum spatial window as a circle with a 125 km radius.

In addition, as eastern DRC is heavily affected by armed conflicts, we compared differences in the weekly number of conflict events reported in cholera-endemic provinces (Haut Katanga, Haut Lomami, Ituri, North Kivu, South Kivu, and Tanganyika) according to the observed geographic spread modalities of outbreaks. Given this variable followed a skewed distribution, we used medians to assess whether differences found in our study were statistically significant using the Kruskal-Wallis test and Wilcoxon-rank sum tests. The statistical analyses were performed with R^®^ version 3.6.1.

### Ethics approval

Ethics approval was not required because this study was carried out with routinely collected surveillance data and aggregated at the HZ level.

## Results

### Summary of major outbreak expansions of cholera since its introduction in eastern DRC

A list of 1,288 studies and reports were identified using the search terms. We removed 384 records due to duplication, and then we screened 904 records of which 889 were considered not relevant according to titles and abstracts or full texts that did not address the dynamics of the spread of cholera in and from eastern DRC. 15 studies and reports were eligible and detailed in Table 1.

**Table 1:** Summaries of the 15 studies and reports considered as relevant in our study.

Citation	Type	Periods	Geographical context	Approach
Schyns.1979. Cholera in Eastern Zaire, 1978	Research	1978	Eastern Zaïre	Reconstruction of the spatio-temporal evolution and epidemiological analysis
Malengreau. 1979. The cholera epidemic in Eastern Zaire in 1978.	Research	1978	Eastern Zaïre	Reconstruction of the spatio-temporal evolution and epidemiological analysis
Carme. 1983. L’implantation et l’extension du choléra en Afrique Noire: 1970–1980	Research	1970–1980	West Africa, East Africa, and Southern Africa	Reconstruction of the spatio-temporal evolution
Rémy & Dejours. 1988. L’Africanisation du choléra	Research	1970–1985	North East Africa, East Central Africa, Southern Africa, West Central Africa, and West Africa	Reconstruction of the spatio-temporal evolution
Swerdlow & Isaäcson. 1994. The Epidemiology of Cholera in Africa.	Research	1972–1991	Africa	Reconstruction of the epidemic and epidemiological analyses
World Health Organization. 1997—Health situation in Rwandan refugee camp in Zaire.	Report of disease outbreak	1997	North-eastern Zaïre	Reconstruction and description of the epidemic
Centers for Disease Control and Prevention. 1998. Cholera Outbreak among Rwandan Refugees—Democratic Republic of Congo, April 1997.	Report of disease outbreak	1997	North-eastern Zaïre	Description of the epidemic
World Health Organization. 1997—Cholera in Zaire.	Report of disease outbreak	1997	North-eastern Zaïre	Description of the epidemic
Bompangue. 2008. Lakes as Source of Cholera Outbreaks, Democratic Republic of Congo.	Research	2002–2005	South-Eastern and Central DRC	Reconstruction of the spatio-temporal evolution and epidemiological analyses
UNICEF. 2011. UNICEF fights "one of the worst ever" cholera outbreaks in West and Central Africa	Report of disease outbreak	2011	The West Congo Basin (DRC, Congo and the Central African Republic)	Description of the epidemic
Bompangue. 2012. Cholera ante portas–The re-emergence of cholera in Kinshasa after a ten-year hiatus	Research	1996–2011	Kinshasa (western DRC)	Reconstruction of the spatio-temporal evolution (2011 epidemic) and epidemiological analyses
Weill. 2017. Genomic history of the seventh pandemic of cholera in Africa	Research	1966–2014	Africa	Reconstruction of the spatio-temporal evolution through genomic analysis
Moore. 2018. Epidemiological study of cholera hotspots and epidemiological basins in East and Southern Africa. In-depth report on cholera epidemiology in Angola	Report	2006–2018	Eastern DRC, western DRC, and Angola	Epidemiological analyses
Ingelbeen. 2019. Recurrent cholera outbreaks, Democratic Republic of the Congo, 2008–2017	Research	2008–2017	DRC	Reconstruction of the spatio-temporal evolution and epidemiological analyses
Breurec. 2021. Seventh pandemic *Vibrio cholerae* O1 sublineages, Central African Republic	Research	1997–2016	Eastern DRC, western DRC, and Central African Republic	Reconstruction of the spatio-temporal evolution through genomic analysis

**Fig 2:**
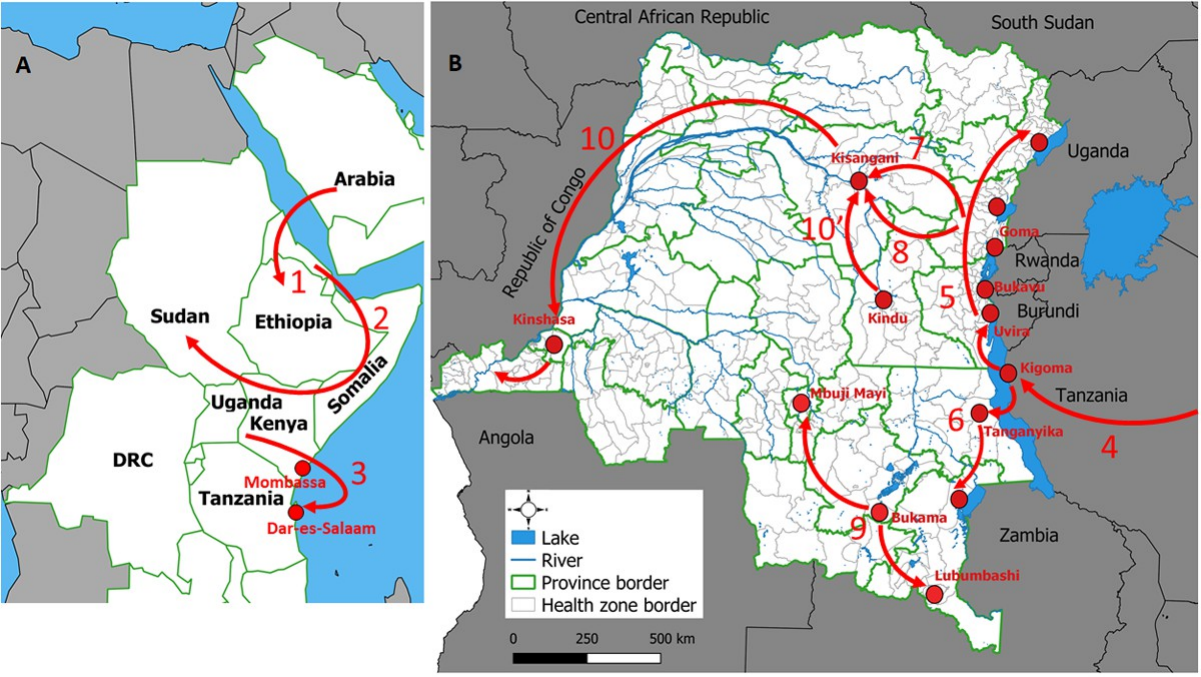
A and B. Major outbreaks expansions of cholera documented since its introduction in eastern DRC in 1978. Sources: Fig 2A [29] and Fig 2B [30].

The introduction of cholera in 1978 in eastern DRC resulted from the importation of the disease into East Africa via Ethiopia from the Middle East in 1970 (number 1, Fig 2A) [14, 34–36]. Since then, neighboring countries were affected: Djibouti, Somalia, Sudan, Uganda and Kenya (number 2, Fig 2A) [34, 35]. In 1974, cholera spread from the shores of Lake Victoria to the Indian Ocean coastline and then reached Dar-es-Salaam (1977) (number 3, Fig 2A) [35].

Fig 2B summarized the following diffusion dynamics: 4 –From the Tanzanian cost to inland areas, Kigoma (April, 1978) [34, 35]; 5 –Northward from Kigoma to the Congolese lakeside areas of lakes Tanganyika, Kivu, Edward and Albert between June and December [5, 34, 35]; 6 –Southward from Kigoma to Kalemie (May, 1978), and then to the shores of Lake Mweru (1981) [5, 6, 34, 35]; 7 –Westward from the GLR to Kisangani (March, 1979) [6, 35]; 8 –Outbreaks occurred in temporary refugee camps after fleeing from the former camps in eastern DRC and other provinces (Equateur and Kinshasa) (1997) [37–39]; 9 –From Bukama to Mbuji Mayi and Lubumbashi (2002) [8]; 10 and 10’–Westward to non-endemic provinces of western DRC (2011–2012 and 2015–2017) [12, 13, 15]. Neighboring countries of the West Congo Basin were affected during these outbreak expansions [14, 15, 40, 41].

### Geographic spread of cholera from endemic eastern areas: Modalities and likely main routes

Using maps of propagation of cholera cases per week and per HZ (S1 Fig), the geographic spread modalities of the disease were described as follows: (i) The spread within endemic eastern provinces including: along lakeside HZs in the same province or in two bordering provinces, from lake areas to adjacent non-endemic HZs (directly sharing their borders) in the same and/or neighboring province, and to non-adjacent HZs (not directly sharing their borders) in the same and/or neighboring province; (ii) The spread outside the endemic provinces to HZs in non-endemic provinces of eastern DRC; (iii) The spread outside the endemic provinces to the HZs in non-endemic provinces of central DRC; (iv) The spread outside the endemic provinces to the HZs in non-endemic provinces of western DRC.

**Fig 3:**
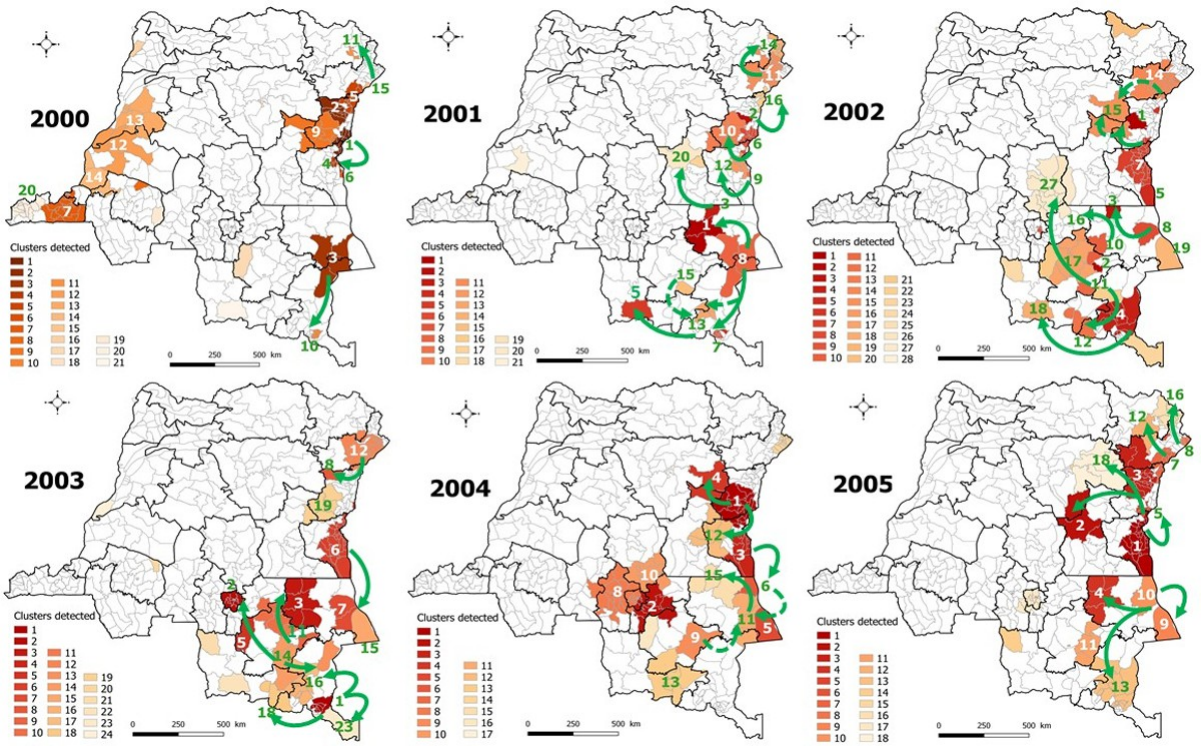
Spatiotemporal clusters of cholera cases, DRC, 2000–2005. Arrows indicate the likely routes of spread. Dashed arrows represent two probable routes at the same period. Republished from [30] under a CC BY license, with permission from [Claire Halleux], original copyright [2021].

Fig 3 described the spatiotemporal clusters of cholera cases identified and the likely routes of spread of outbreaks from endemic eastern areas during 2000–2005. Details on space-time clustering results were summarized in S1–S6 Tables. Cholera most likely spread to non-endemic eastern provinces (Maniema and Tshopo) from areas around Lake Kivu through the westernmost HZs of North Kivu, Pinga and Walikale. Those provinces were also affected in a westerly direction from lakeside HZs of Lake Albert (Ituri) and Lake Tanganyika. The disease reached the city of Lubumbashi in southeastern DRC from the shores of lakes Mweru (Haut Katanga), Tanganyika, and Upemba (Haut Lomami). The latter was at the origin of the spread of cholera to the central part of the DRC. Clusters detected in western DRC in 2000 were related to the dynamics that started in previous years.

**Fig 4:**
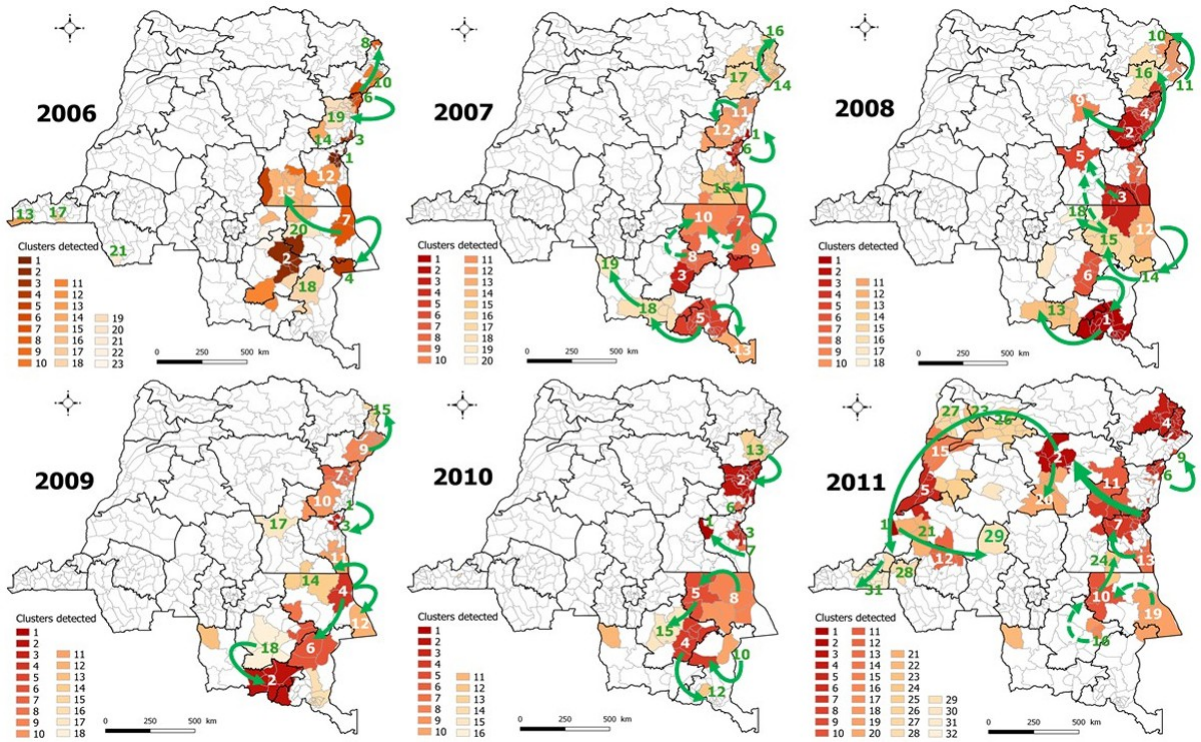
Spatiotemporal clusters of cholera cases, DRC, 2006–2011. Arrows indicate the likely routes of spread. Dashed arrows represent two probable routes at the same period. Republished from [30] under a CC BY license, with permission from [Claire Halleux], original copyright [2021].

Fig 4 depicted the spatiotemporal clusters of cholera cases and the likely routes of spread of outbreaks from endemic eastern areas during 2006–2011. Detailed results of cluster detection analyses were shown in S7–S12 Tables. The western part of Tanganyika province was successively affected from the shores of Lake Tanganyika, Lake Mweru and Lake Upemba, and then the disease progressed to the southern part of Maniema (2006) and the city of Kindu (2008). Clusters detected in southwestern DRC in 2006 were epidemiologically linked to the outbreak in neighboring country, Angola. The spread of cholera to the city of Kisangani (Tshopo) and then to non-endemic western provinces was most likely initiated around Lake Kivu via the Walikale HZ in North Kivu (2010–2011).

**Fig 5:**
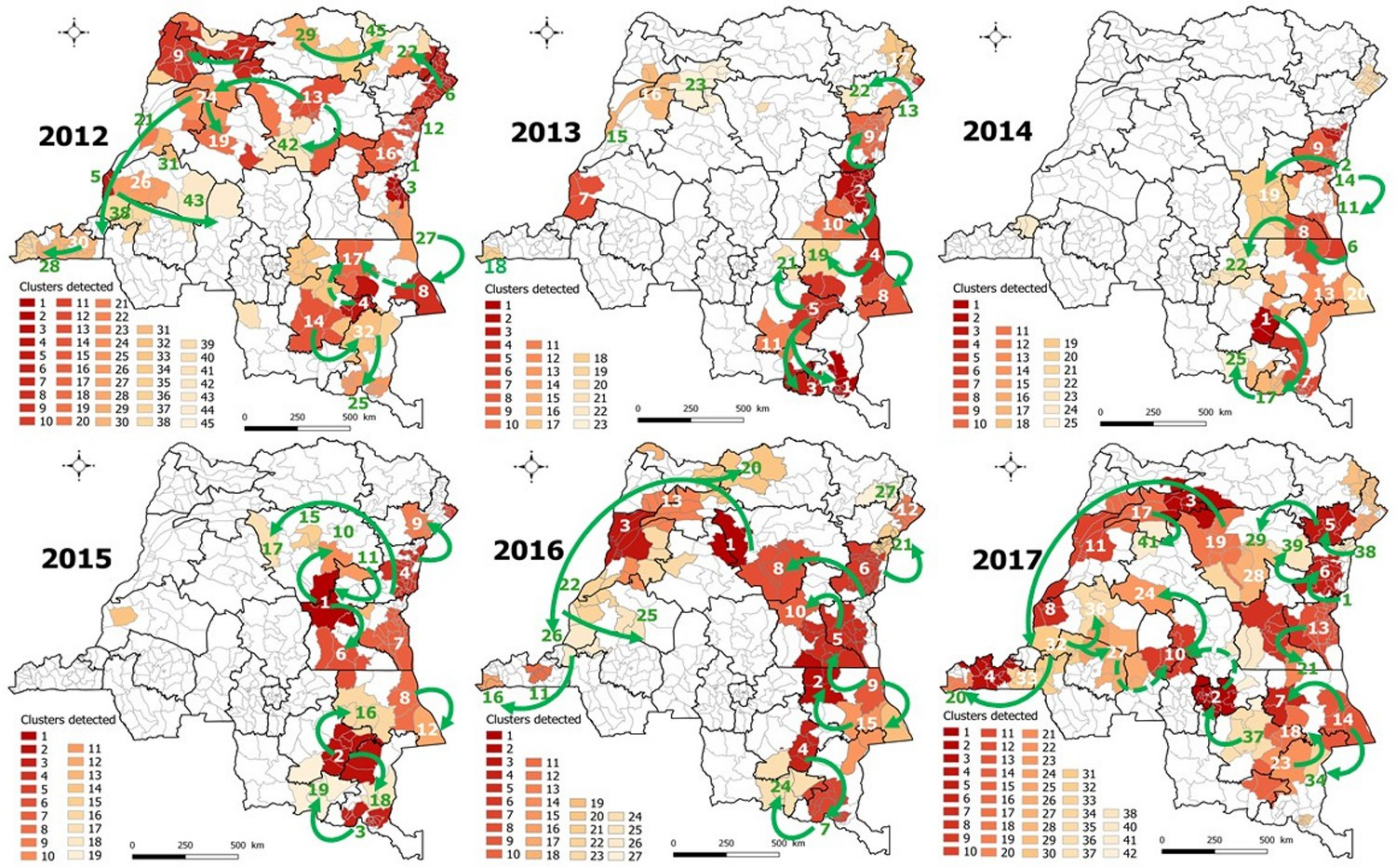
Spatiotemporal clusters of cholera cases, DRC, 2012–2017. Arrows indicate the likely routes of spread. Dashed arrows represent two probable routes at the same period. Republished from [30] under a CC BY license, with permission from [Claire Halleux], original copyright [2021].

Fig 5 illustrated the space-time clusters of cholera cases detected and the likely routes of spread of outbreaks from endemic eastern areas during 2012–2017. The clusters were detailed in S13–S18 Tables. All the spatial spread modalities were observed during 2012–2017. Beyond the east-west propagation, several most likely routes of cholera spread to Kisangani were identified: from Kindu (initiated around Lake Kivu and then via the north of Maniema), from lake areas bordering lakes Kivu and Albert respectively via Pinga and Walikale HZs, and the western part of Ituri. The western HZs of Tanganyika province were affected from lakes Upemba and Tanganyika by northward and westward spread, respectively. A southward spread of cholera was repeatedly observed from the lakeside HZs of Upemba to Lubumbashi before reaching other HZs in Lualaba province. Outbreak affecting provinces of Lomami and Kasaï Oriental was also originated around Lake Upemba.

### Comparison of the weekly number of conflicts reported in endemic eastern provinces according to geographic spread modalities of cholera outbreaks

**Fig 6:**
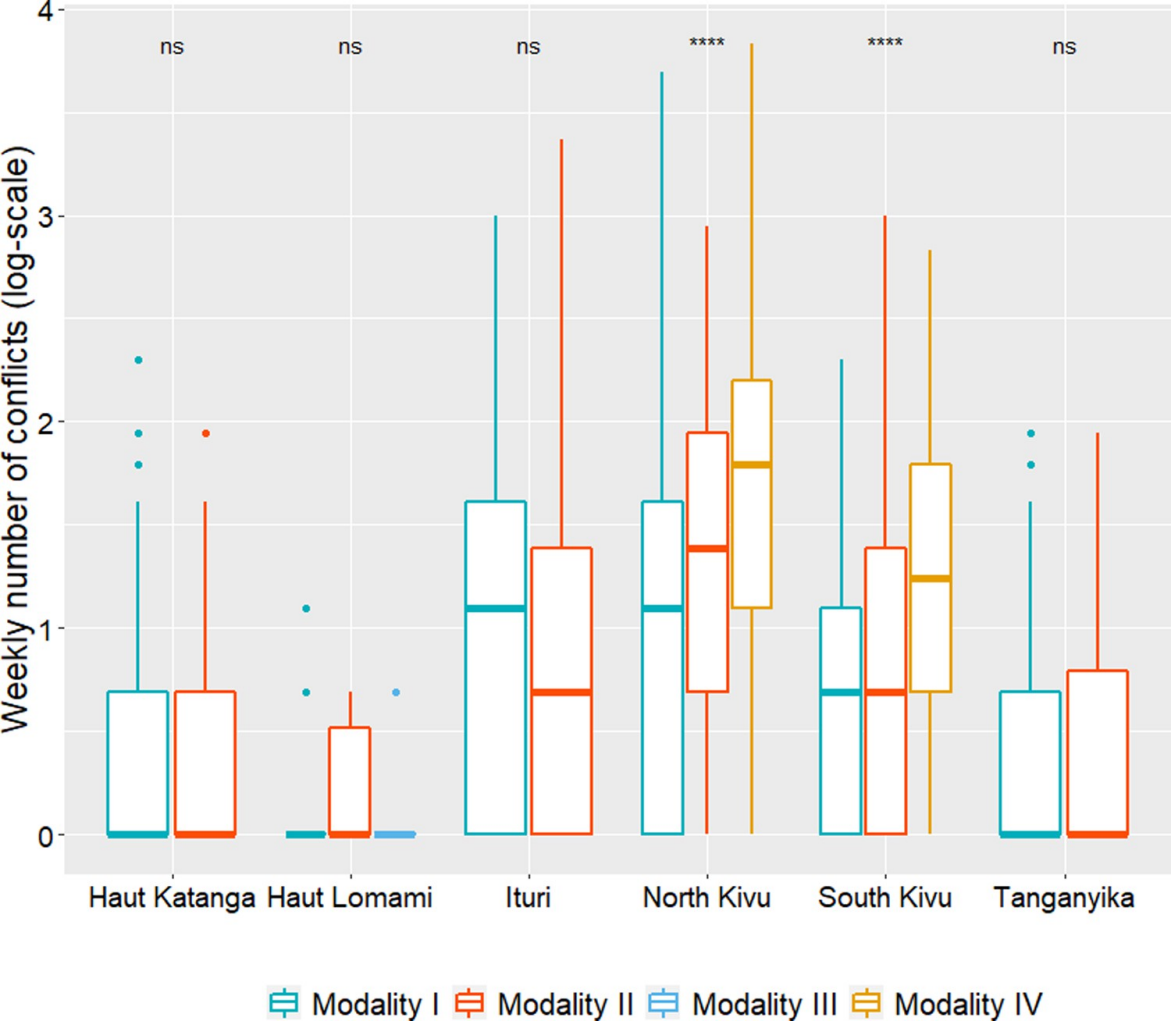
Boxplots of the weekly numbers of conflict events reported in eastern endemic provinces by geographic spread modalities of cholera outbreaks.

The Kruskal-Wallis test showed that the median weekly number of conflict events reported in endemic eastern provinces was significantly greater for Modality IV than for Modality II and Modality I in North Kivu (*X*^2^ = 159.58, df = 2, *p* < 0.001) and South Kivu (*X*^2^ = 100.97, df = 2, *p* < 0.001) (Fig 6). Moreover, the Wilcoxon-rank sum tests for each pairwise comparison of geographic spread modalities of cholera confirmed that all differences between the weekly number of conflict events reported in those provinces were statistically significant (Table 2).

**Table 2:** Wilcoxon-rank sum test results.

Pairwise comparisons	Medians	Test statistics (W)	p-value
*North Kivu*			
Modality I–Modality II	0.00–0.69	21411	< 0.001
Modality I–Modality IV	0.00–1.79	13510	< 0.001
Modality II–Modality IV	0.69–1.79	15630	< 0.001
*South Kivu*			
Modality I–Modality II	0.00–0.00	22695	0.004
Modality I–Modality IV	0.00–1.10	5474.5	< 0.001
Modality II–Modality IV	0.00–1.10	9165	< 0.001

## Discussion

The present study described four geographic spread modalities of cholera outbreaks from endemic eastern foci and their main likely propagation routes according to the affected regions at the national level: around lake areas and to their surroundings in endemic eastern provinces, to other areas respectively located into eastern, central and western non-endemic provinces. In addition, according to the observed spread modalities, the higher the weekly number of conflict events reported in endemic eastern provinces (North and South Kivu), the greater the geographic spread of cholera across the country.

Understanding the spread dynamics of infectious diseases is of utmost importance in the anticipation and control of their epidemics [42]. In this study, although the processes of diffusion of cholera epidemics from source areas are multidirectional, main likely routes of spread have emerged as preferential trajectories. The propagation route from the areas around Lake Kivu to the westernmost HZs of North Kivu, Pinga and Walikale, via the main roads would probably be the most involved in the spread of cholera out of endemic eastern DRC to the northern part of Maniema, including the city of Kindu, and to the city of Kisangani (Tshopo). The latter could also be affected by the westward spread of the disease from the shores of Lake Albert in Ituri through the main road network. Furthermore, the lakeside areas of Haut Lomami and Tanganyika would be the most probable original foci of outbreaks affecting the western part of Tanganyika province (Ankoro, Manono, Kabalo, Mbulala, and Kongolo). From there, the disease could gradually progress to the southern part of Maniema, then to Kindu as well as to Kisangani. Note that Kisangani serves as a transport hub in the east-west spread of cholera reported in the literature [12–15].

Other main likely routes of disease spread were observed. Lubumbashi, the capital of Haut Katanga in southeastern DRC, would be affected by the southward spread of outbreaks from areas bordering either Lake Upemba or Lake Mweru, most likely via major roads. The lake areas of Haut Lomami would also be the main source of epidemics affecting the eastern part of Grand Kasai in central DRC through the railways.

The main propagation routes of cholera described as preferential trajectories through major roads, rivers, and railways are consistent with other findings which considered these transport networks as key drivers of the disease spread through population movements [8, 13, 43, 44]. Targeted prevention and control efforts should also take into account these preferential trajectories from known endemic areas in order to effectively eliminate the disease in the DRC.

The geographic spread of cholera epidemics out of endemic eastern DRC may be linked to the exacerbation of conflict events reported in this region, particularly in North and South Kivu. Interestingly, this finding is supported by evidence of increased spatial spread of infectious diseases in countries heavily affected by conflicts [20–22]. The humanitarian crisis in Yemen, resulting from the ongoing devastating war, led to the largest and fastest spreading cholera epidemic worldwide [20, 45–47]. In our context, these dynamics of spread would be consecutive to the migration flows of thousands of internally displaced persons (IDPs) due to war and conflict situations. The latter also negatively impact the health system and other infrastructures that lose the capacity to provide even basic services [16]. As a result, conflict-affected populations are at increased risk of food insecurity, shortages of potable water, and poor sanitation and hygiene practices in host sites due to rapid and massive relocation [17, 18]. It has been shown previously that a one-day interruption in water supply is followed by a substantial increase in the incidence rate of suspected cholera cases within 12 days [48].

Nevertheless, we also found other propagation routes of epidemics out of endemic eastern provinces initiated from areas less affected by conflicts. As previously suggested, these dynamics of spread are likely associated with seasonal fishing and fish trading activities [10, 11]. Although this explained the spread of cholera to the eastern part of the central provinces of the DRC in 2002 [8, 49], an explosive outbreak occurred 15 years later in this region, following a similar pattern, under a conflict-fueled humanitarian crisis that led to the collapse of almost the entire health system [50]. These observations corroborate the idea that the risk of geographic spread of cholera is multifactorial. In addition to conflict events [16, 51], it may require the interaction of ecological, socioeconomic and behavioral factors involving human migratory dynamics [52].

This study presented a number of limitations. First, we used data on suspected cholera cases. It may have over or underestimated the true burden of cholera. However, a recent assessment on the level of adequacy of the 15 weekly reported epidemic prone diseases monitored by the DRC’s surveillance system demonstrated that the use of these data may be relevant for epidemiological or public health research purposes [53]. Furthermore, another assessment of IDSR key performance indicators showed that the DRC figures among the African countries with high coverage of IDSR implementation at subnational [54]. Second, the lack of genomic data from *Vibrio cholerae* O1 isolates. Phylogenetic analysis would have allowed determination of stable propagation routes of circulating cholera strains from the different endemic foci. Nonetheless, to our knowledge, this is the first epidemiological study carried out in such a long period (18 years) that explored the geographic spread modalities and their most likely preferential trajectories from endemic eastern areas. This provides additional understanding elements to the current state of knowledge on the spread dynamics of cholera in the DRC. Third, we just looked at the weekly number of conflict events in endemic provinces, without including non-endemic conflict events. Considering that endemic provinces account for 73% of reported conflict events in the country [27], two-thirds of which occur in North and South Kivu, we hypothesized that the exacerbation of conflicts in eastern DRC would be responsible for the spread of cholera to far-off areas in non-endemic provinces. Our results revealed that the most likely routes of spread involved in these dynamics would originate mainly from endemic areas in North and South Kivu. However, the few dynamics of spread out of endemic provinces initiated from eastern areas less affected by conflicts further highlight the key role of population movements related to IDPs on the one hand, and commercial activities on the other. There is need for futures studies to explore how conjunctural and structural population movements may affect the geographic spread of cholera from endemic eastern areas.

## Conclusions

The modalities and the likely main routes of geographic spread of cholera outbreaks from the source areas described in this study highlight that the dynamics of the disease’s expansion follow a fairly well-defined spatial logic, and can therefore be predicted. These results could contribute to the development of a plan to build resilience in HZs iteratively affected by epidemic waves spreading from endemic areas to achieve the 2030 goals of reducing cholera as a major public health threat [55]. Further phylogenetic researches will help to confirm the likely preferred routes of spread of cholera epidemics identified in the DRC.

The exacerbation of conflict events reported in eastern DRC is most likely associated with the spread of outbreaks affecting areas increasingly distant from endemic foci. This implies the possibility of setting up early warning systems including monitoring of conflict dynamics to anticipate the risk of geographic spread of cholera in the DRC.

## Supporting information

S1 Fig Distribution of weekly suspected cholera cases by health zone, DRC, 2000–2017. Republished from [30] under a CC BY license, with permission from [Claire Halleux], original copyright [2021].
(DOCX)

S1 Data (TXT)

S2 Data (TXT)

S1 Table Spatiotemporal clusters of cholera cases, DRC, 2000. (DOCX)

S2 Table Spatiotemporal clusters of cholera cases, DRC, 2001. (DOCX)

S3 Table Spatiotemporal clusters of cholera cases, DRC, 2002. (DOCX)

S4 Table Spatiotemporal clusters of cholera cases, DRC, 2003. (DOCX)

S5 Table Spatiotemporal clusters of cholera cases, DRC, 2004. (DOCX)

S6 Table Spatiotemporal clusters of cholera cases, DRC, 2005. (DOCX)

S7 Table Spatiotemporal clusters of cholera cases, DRC, 2006. (DOCX)

S8 Table Spatiotemporal clusters of cholera cases, DRC, 2007. (DOCX)

S9 Table Spatiotemporal clusters of cholera cases, DRC, 2008. (DOCX)

S10 Table Spatiotemporal clusters of cholera cases, DRC, 2009. (DOCX)

S11 Table Spatiotemporal clusters of cholera cases, DRC, 2010. (DOCX)

S12 Table Spatiotemporal clusters of cholera cases, DRC, 2011. (DOCX)

S13 Table Spatiotemporal clusters of cholera cases, DRC, 2012. (DOCX)

S14 Table Spatiotemporal clusters of cholera cases, DRC, 2013. (DOCX)

S15 Table Spatiotemporal clusters of cholera cases, DRC, 2014. (DOCX)

S16 Table Spatiotemporal clusters of cholera cases, DRC, 2015. (DOCX)

S17 Table Spatiotemporal clusters of cholera cases, DRC, 2016. (DOCX)

S18 Table Spatiotemporal clusters of cholera cases, DRC, 2017. (DOCX)

## References

[1] de Magny GC, Murtugudde R, Sapiano MRP, Nizam A, Brown CW, Busalacchi AJ, et al. Environmental signatures associated with cholera epidemics. PNAS. 2008;105(46):17676–81. doi: 10.1073/pnas.0809654105 19001267 PMC2584748

[2] Sack DA, Sack RB, Nair GB, Siddique AK. Cholera. Lancet. 2004;(363):223–33. 10.1016/s0140-6736(03)15328-7 14738797

[3] Harris JB, LaRocque RC, Qadri F, Ryan ET, Calderwood SB. Cholera. Lancet. 2012;(379):2466–76. 22748592 10.1016/S0140-6736(12)60436-X PMC3761070

[4] World Health Organization. Cholera case and death numbers by country. Wkly Epidemiol Rec. Available from: http://www.who.int/cholera/en/.

[5] Malengreau M, Gillieaux M, De Feyter M, Wittman L. The cholera epidemic in Eastern Zaire in 1978. Ann Soc Belg Med Trop. 1979;59(4):401–12. 543737

[6] Schyns C, Fossa A, Mutombo-Nfenda null, Kabuyahiya null, Hennart P, Piot P, et al. Cholera in Eastern Zaire, 1978. Ann Soc Belg Med Trop. 1979;59(4):391–400. 543736

[7] Goma Epidemiology Group. Public health impact of Rwandan refugee crisis: what happened in Goma, Zaire, in July, 1994? Lancet. 1995;345(8946):339–44. 7646638

[8] Bompangue D, Giraudoux P, Handschumacher P, Piarroux M, Sudre B, Ekwanzala M, et al. Lakes as Source of Cholera Outbreaks, Democratic Republic of Congo. Emerg Infect Dis. 2008;14(5):798–800. doi: 10.3201/eid1405.071260 18439365 PMC2600234

[9] Bompangue D, Giraudoux P, Piarroux M, Mutombo G, Shamavu R, Sudre B, et al. Cholera Epidemics, War and Disasters around Goma and Lake Kivu: An Eight-Year Survey. PLoS Negl Trop Dis. 2009;3(5). 10.1371/journal.pntd.0000436 PMC2677153 19436726

[10] Bompangue Nkoko D, Giraudoux P, Plisnier P-D, Tinda AM, Piarroux M, Sudre B, et al. Dynamics of cholera outbreaks in great lakes region of Africa, 1978–2008. Emerg Infect Dis. 2011;17(11):2026–34. doi: 10.3201/eid1711.110170 22099090 PMC3310557

[11] Muyembe JJ, Bompangue D, Mutombo G, Akilimali L, Mutombo A, Miwanda B, et al. Elimination of Cholera in the Democratic Republic of the Congo: The New National Policy. J Infect Dis. 2013;208(suppl_1):S86–91. doi: 10.1093/infdis/jit204 24101651

[12] Bompangue D, Vesenbeckh SM, Giraudoux P, Castro M, Muyembe J-J, Ilunga BK, et al. Cholera ante portas–The re-emergence of cholera in Kinshasa after a ten-year hiatus. PLOS Curr Disasters. 2012;4:RRN1310. doi: 10.1371/currents.RRN1310 22453903 PMC3299488

[13] Ingelbeen B, Hendrickx D, Miwanda B, van der Sande MAB, Mossoko M, Vochten H, et al. Recurrent Cholera Outbreaks, Democratic Republic of the Congo, 2008–2017. Emerg Infect Dis. 2019;25(5):856–64. doi: 10.3201/eid2505.181141 31002075 PMC6478228

[14] Weill F-X, Domman D, Njamkepo E, Tarr C, Rauzier J, Fawal N, et al. Genomic history of the seventh pandemic of cholera in Africa. Science. 2017;358(6364):785–9. doi: 10.1126/science.aad5901 29123067

[15] Breurec S, Franck T, Njamkepo E, Mbecko J-R, Rauzier J, Sanke-Waïgana H, et al. Seventh Pandemic Vibrio cholerae O1 Sublineages, Central African Republic. Emerg Infect Dis. 2021;27(1):262–6. doi: 10.3201/eid2701.200375 33350910 PMC7774542

[16] Gayer M, Legros D, Formenty P, Connolly MA. Conflict and emerging infectious diseases. Emerg Infect Dis. 2007;13(11):1625–31. doi: 10.3201/eid1311.061093 18217543 PMC3375795

[17] Als D, Meteke S, Stefopulos M, Gaffey MF, Kamali M, Munyuzangabo M, et al. Delivering water, sanitation and hygiene interventions to women and children in conflict settings: a systematic review. BMJ Glob Health. 2020;5(Suppl 1):e002064. doi: 10.1136/bmjgh-2019-002064 32641288 PMC7348465

[18] Sikder M, Daraz U, Lantagne D, Saltori R. Water, sanitation, and hygiene access in southern Syria: analysis of survey data and recommendations for response. Confl Health. 2018;12(1):17. 29713372 10.1186/s13031-018-0151-3 PMC5911958

[19] Ramesh A, Blanchet K, Ensink JHJ, Roberts B. Evidence on the Effectiveness of Water, Sanitation, and Hygiene (WASH) Interventions on Health Outcomes in Humanitarian Crises: A Systematic Review. PLOS ONE. 2015;10(9):e0124688. doi: 10.1371/journal.pone.0124688 26398228 PMC4580573

[20] Bin Ghouth AS. The Multi-Epidemics in Yemen: the Ugly Face of the War. Ann Infect Dis Epidemiol. 2018;3(2):1033.

[21] Awofeso N, Aldbak K. Cholera, Migration, and Global Health–A Critical Review. Int J Travel Med Glob Health. 2018;6(3):92–9.

[22] Dureab FA, Shibib K, Al-Yousufi R, Jahn A. Yemen: Cholera outbreak and the ongoing armed conflict. J Infect Dev Ctries. 2018;12(05):397–403. doi: 10.3855/jidc.10129 31865306

[23] Mondiale Banque. Données par pays. 2016; Available from: http://donnees.banquemondiale.org/pays.

[24] INS. Enquêtes par grappes à indicateurs multiples, 2017–2018, rapport des résultats de l’enquête. Kinshasa, République Démocratique du Congo. 2019. Available from: https://www.unicef.org/drcongo/rapports/resume-mics-palu-2017-2018.

[25] GTFCC Surveillance Working Group. Interim guidance document on cholera surveillance. 2017. Available from: https://www.who.int/cholera/task_force/GTFCC-Guidance-cholera-surveillance.pdf?ua = 1.

[26] World Health Organization. Guidelines for cholera control. 1993. Available from: https://apps.who.int/iris/handle/10665/36837.

[27] ACLED. Armed Conflict Location & Event Data Project (ACLED) Codebook: Version 8. 2017. Available from: https://reliefweb.int/report/world/armed-conflict-location-event-data-project-acled-codebook-version-8-2017.

[28] Raleigh C, Linke A, Hegre H, Karlsen J. Introducing ACLED: An Armed Conflict Location and Event Dataset. J Peace Res. 2010;47(5):651–60.

[29] Humanitarian Data Exchange. Large Scale International Boundaries (LSIB). Available from: https://data.humdata.org/dataset/large-scale-international-boundaries-lsib.

[30] Humanitarian Data Exchange. Zones de santé (RDC) / Health zones (DRC). Available from: https://data.humdata.org/dataset/zones-de-sante-rdc.

[31] Kulldorff M. SaTScanTM v9.6: Software for the Spatial and Space-Time Scan Statistics. Information Management Services. 2018. Available from: https://www.satscan.org.

[32] Kulldorff M, Heffernan R, Hartman J, Assunção R, Mostashari F. A Space–Time Permutation Scan Statistic for Disease Outbreak Detection. PLOS Med. 2005;2(3):e59. doi: 10.1371/journal.pmed.0020059 15719066 PMC548793

[33] Horwood PF, Karl S, Mueller I, Jonduo MH, Pavlin BI, Dagina R, et al. Spatio-temporal epidemiology of the cholera outbreak in Papua New Guinea, 2009–2011. BMC Infect Dis. 2014;14(1):449. doi: 10.1186/1471-2334-14-449 25141942 PMC4158135

[34] Carme B, Mavumu Ngwak M, Trape J-F, Yala F, Felix M. L’implantation et l’extension du choléra en Afrique Noire: 1970–1980. Rev Méd Congo. 1983;2(34):17–29.

[35] Rémy G, Dejours H. L’Africanisation du choléra. Cahiers d’outre-mer. 1988;41(162):105–38.

[36] Swerdlow DL, Isaäcson M. The Epidemiology of Cholera in Africa. Vibrio Cholerae Cholera. 1994;297–307.

[37] World Health Organization. 1997—Health situation in Rwandan refugee camp in Zaire. 1997. Available from: https://www.who.int/csr/don/1997_01_20/en/.

[38] Centers for Disease Control and Prevention. Cholera Outbreak among Rwandan Refugees—Democratic Republic of Congo, April 1997. MMWR Wkly. 1998;47(19):389–91. 9614007

[39] World Health Organization. 1997—Cholera in Zaire. 1997. Available from: https://www.who.int/csr/don/1997_04_15b/en/.

[40] UNICEF. UNICEF fights “one of the worst ever” cholera outbreaks in West and Central Africa—Cameroon. ReliefWeb. 2011. Available from: https://reliefweb.int/report/cameroon/unicef-fights-%E2%80%9Cone-worst-ever%E2%80%9D-cholera-outbreaks-west-and-central-africa.

[41] Moore S, Dunoyer J, Sudre B, Valingot C, Rebaudet S, Piarroux R. Epidemiological study of cholera hotspots and epidemiological basins in East and Southern Africa. In-depth report on cholera epidemiology in Angola. UNICEF. 2018. Available from: http://www.platefor.mywhc.ca/attachments/article/794/Cholera%20epidemiology%20in%20Angola_UNICEF_2018_FINAL.pdf.

[42] Congdon P. Spatiotemporal Frameworks for Infectious Disease Diffusion and Epidemiology. Int J Environ Res Public Health]. 2016;13(12). doi: 10.3390/ijerph13121261 27999420 PMC5201402

[43] Ngwa MC, Liang S, Kracalik IT, Morris L, Blackburn JK, Mbam LM, et al. Cholera in Cameroon, 2000–2012: Spatial and Temporal Analysis at the Operational (Health District) and Sub Climate Levels. PLoS Negl Trop Dis. 2016;10(11):e0005105. doi: 10.1371/journal.pntd.0005105 27855171 PMC5113893

[44] Ngwa MC, Ihekweazu C, Okwor T, Yennan S, Williams N, Elimian K, et al. The cholera risk assessment in Kano State, Nigeria: A historical review, mapping of hotspots and evaluation of contextual factors. PLoS Negl Trop Dis. 2021;15(1):e0009046. doi: 10.1371/journal.pntd.0009046 33465091 PMC7846125

[45] Camacho A, Bouhenia M, Alyusfi R, Alkohlani A, Naji MAM, de Radiguès X, et al. Cholera epidemic in Yemen, 2016–18: an analysis of surveillance data. Lancet Glob Health. 2018;6(6):e680–90. doi: 10.1016/S2214-109X(18)30230-4 29731398 PMC5952990

[46] Federspiel F, Ali M. The cholera outbreak in Yemen: lessons learned and way forward. BMC Public Health. 2018;18(1):1338. doi: 10.1186/s12889-018-6227-6 30514336 PMC6278080

[47] Blackburn CC, Lenze PE, Casey RP. Conflict and Cholera: Yemen’s Man-Made Public Health Crisis and the Global Implications of Weaponizing Health. Health Secur. 2020;18(2):125–31. doi: 10.1089/hs.2019.0113 32324073

[48] Jeandron A, Saidi JM, Kapama A, Burhole M, Birembano F, Vandevelde T, et al. Water Supply Interruptions and Suspected Cholera Incidence: A Time-Series Regression in the Democratic Republic of the Congo. PLOS Med. 2015;12(10):e1001893. doi: 10.1371/journal.pmed.1001893 26506001 PMC4624412

[49] Piarroux R, Bompangue D, Oger P-Y, Haaser F, Boinet A, Vandevelde T. From research to field action: example of the fight against cholera in the Democratic Republic of Congo. Field Actions Sci Rep. 2009;2:69–77.

[50] IFRC. DRC: As Kasai humanitarian crisis reaches new heights, Red Cross expands response to cholera outbreak—Democratic Republic of the Congo. ReliefWeb. 2018. Available from: https://reliefweb.int/report/democratic-republic-congo/drc-kasai-humanitarian-crisis-reaches-new-heights-red-cross-expands.

[51] Kelly-Hope LA. Conflict and Emerging Infectious Diseases. Emerg Infect Dis. 2008;14(6):1004–5. doi: 10.3201/eid1406.080027 18507934 PMC2600301

[52] Kraemer MUG, Hay SI, Pigott DM, Smith DL, Wint GRW, Golding N. Progress and Challenges in Infectious Disease Cartography. Trends Parasitol. 2016;32(1):19–29. doi: 10.1016/j.pt.2015.09.006 26604163

[53] Mandja B-AM, Bompangue D, Handschumacher P, Gonzalez J-P, Salem G, Muyembe J-J, et al. The score of integrated disease surveillance and response adequacy (SIA): a pragmatic score for comparing weekly reported diseases based on a systematic review. BMC Public Health. 2019;19(1):624. doi: 10.1186/s12889-019-6954-3 31118016 PMC6532185

[54] Fall IS, Rajatonirina S, Yahaya AA, Zabulon Y, Nsubuga P, Nanyunja M, et al. Integrated Disease Surveillance and Response (IDSR) strategy: current status, challenges and perspectives for the future in Africa. BMJ Glob Health. 2019;4(4):e001427. doi: 10.1136/bmjgh-2019-001427 31354972 PMC6615866

[55] Global Task Force on Cholera Control. Ending Cholera: A Global Roadmap to 2030. Geneva: World Health Organization. 2017. Available from: http://www.who.int/cholera/publications/global-roadmap/en/.

