# Supplementary material for: Modalities and preferred routes of geographic spread of cholera from endemic areas in eastern Democratic Republic of the Congo
Source: PLoS One. 2022 Feb 7;17(2):e0263160. doi: 10.1371/journal.pone.0263160 (PMC8820636; doi:10.1371/journal.pone.0263160)

**Geographic spread of cholera from endemic eastern areas: modalities and likely routes**

**S1 Fig. Distribution of weekly suspected cholera cases by health zone, DRC, 2000-2017**. Republished from [30] under a CC BY license, with permission from [Claire Halleux], original copyright [2021].
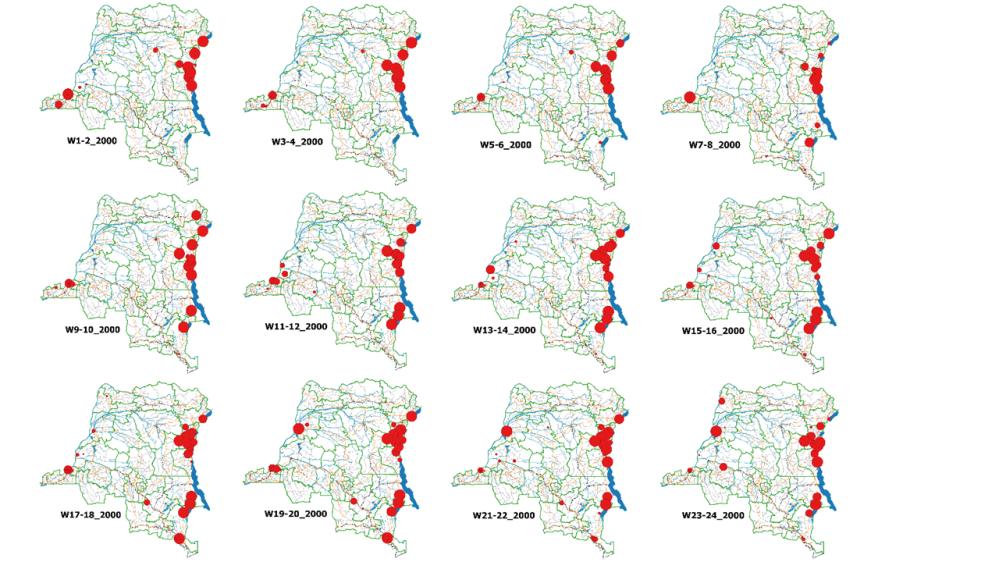

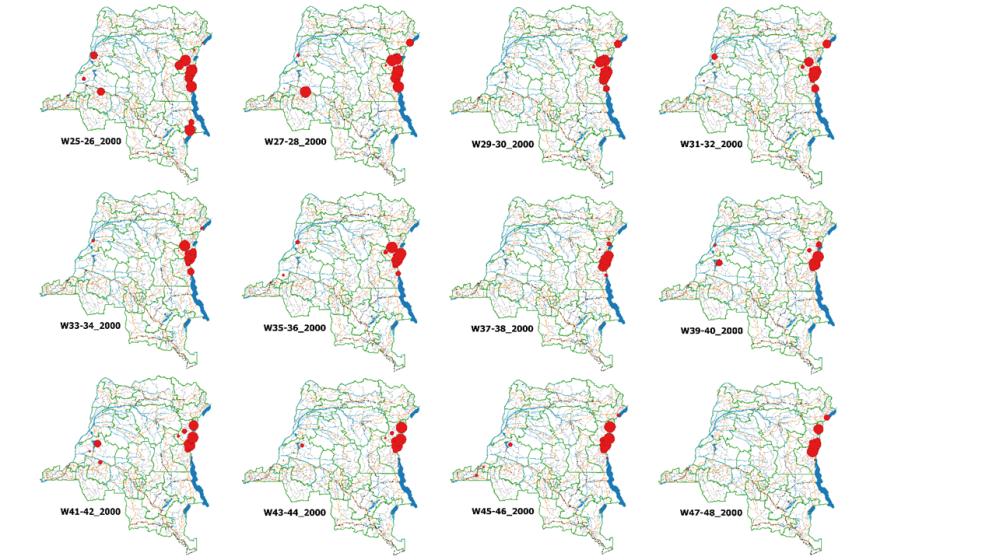

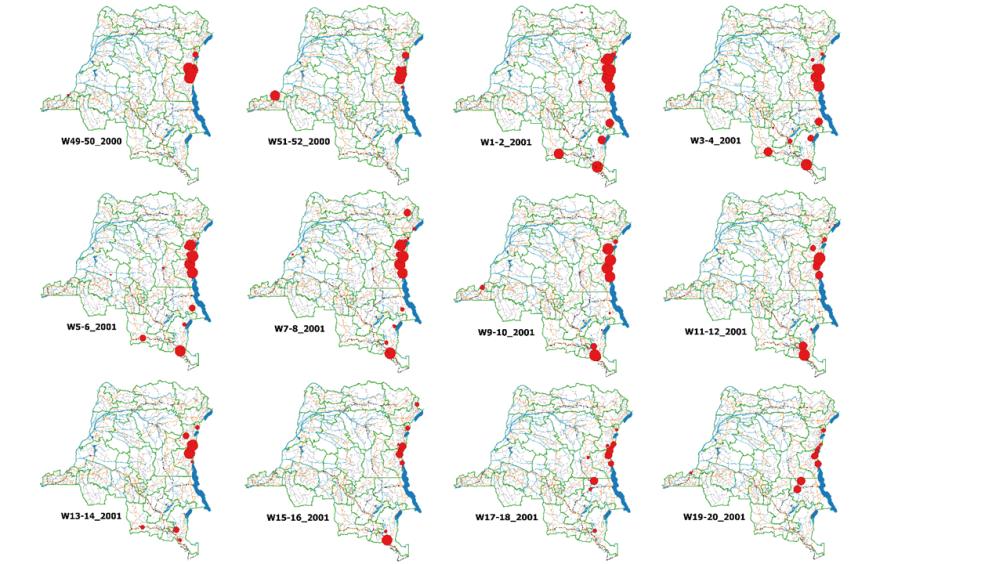

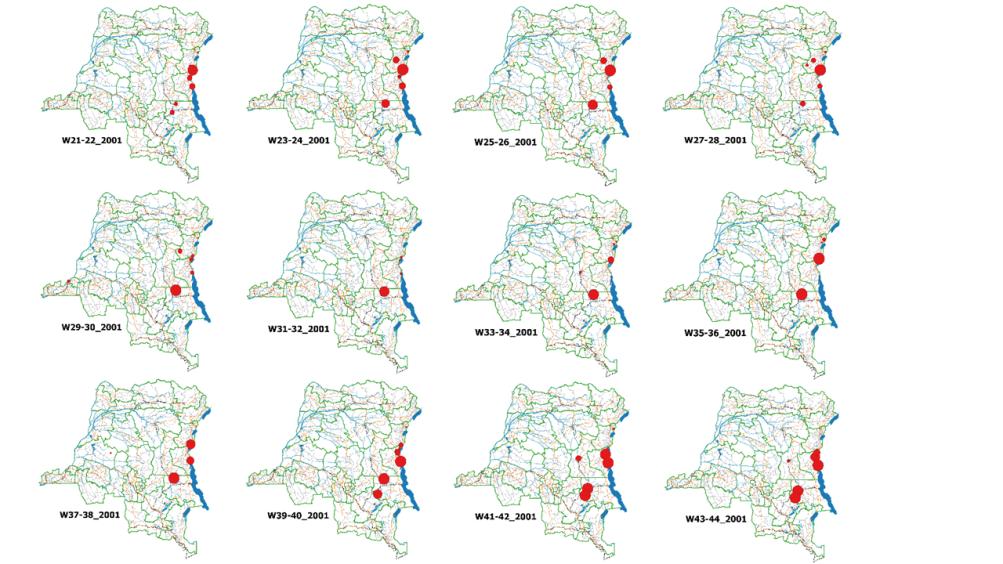

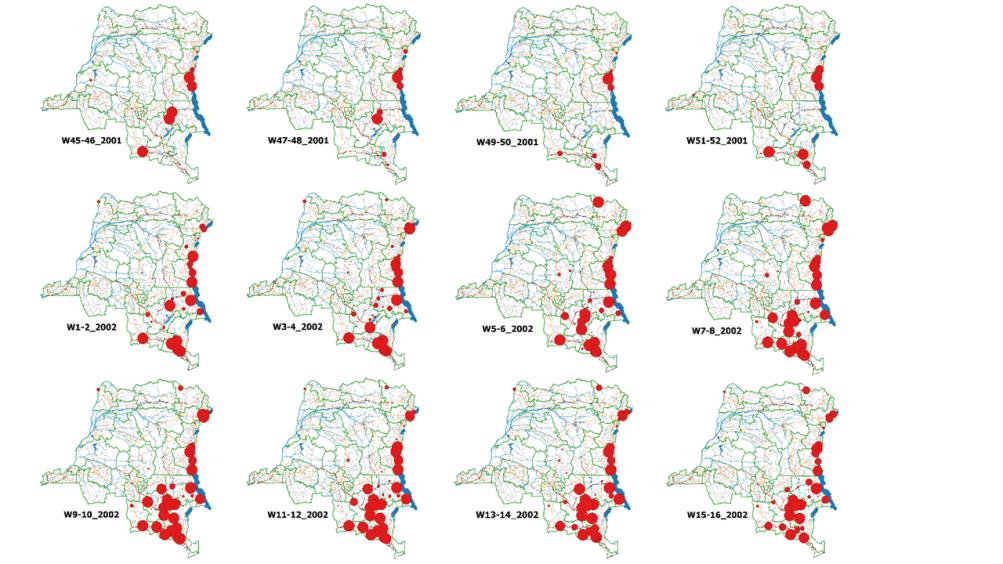

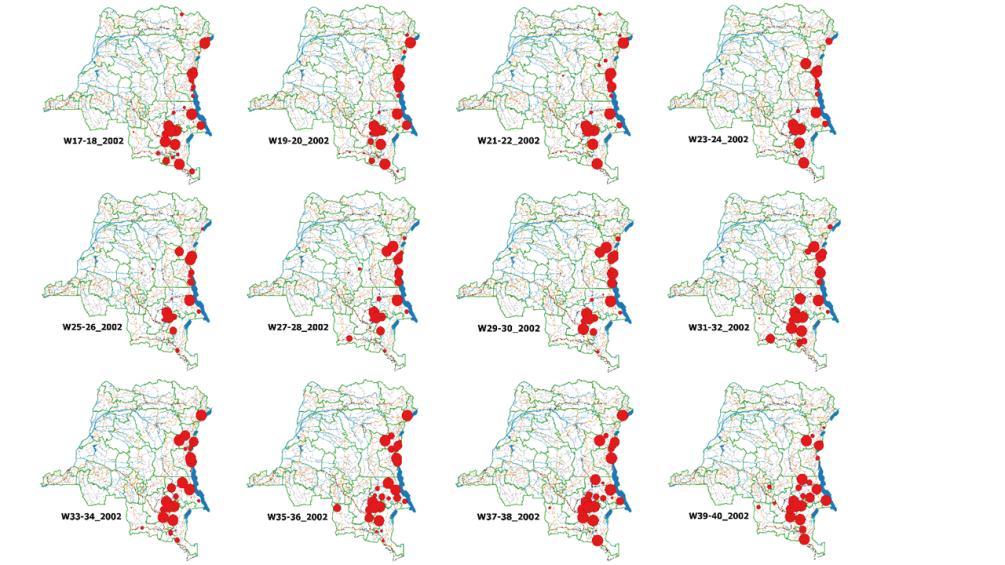

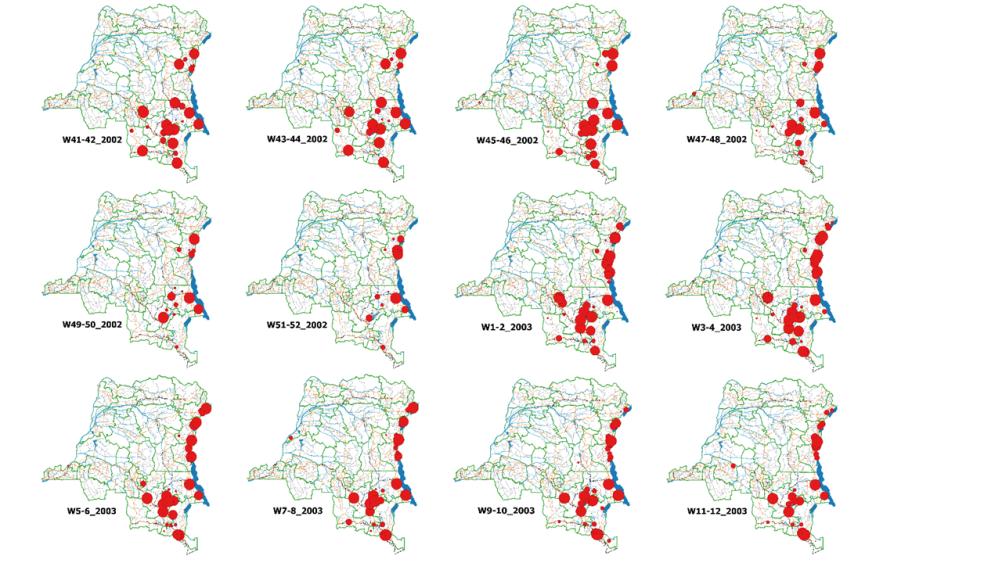

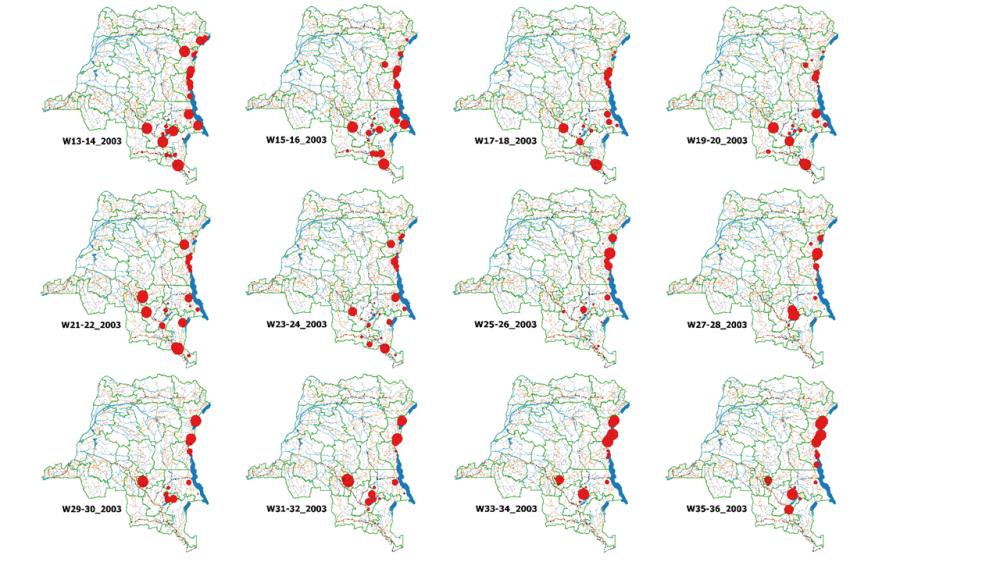

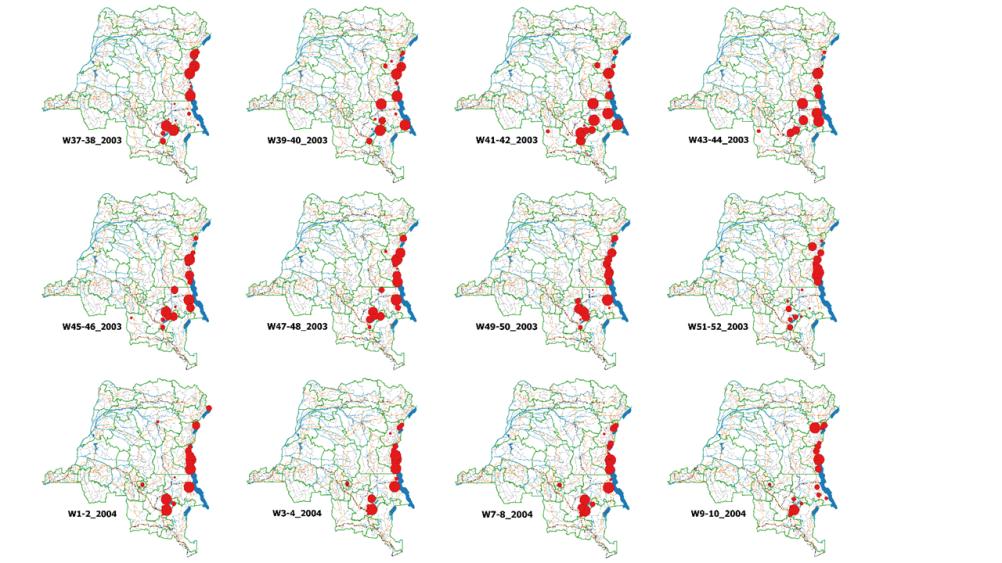

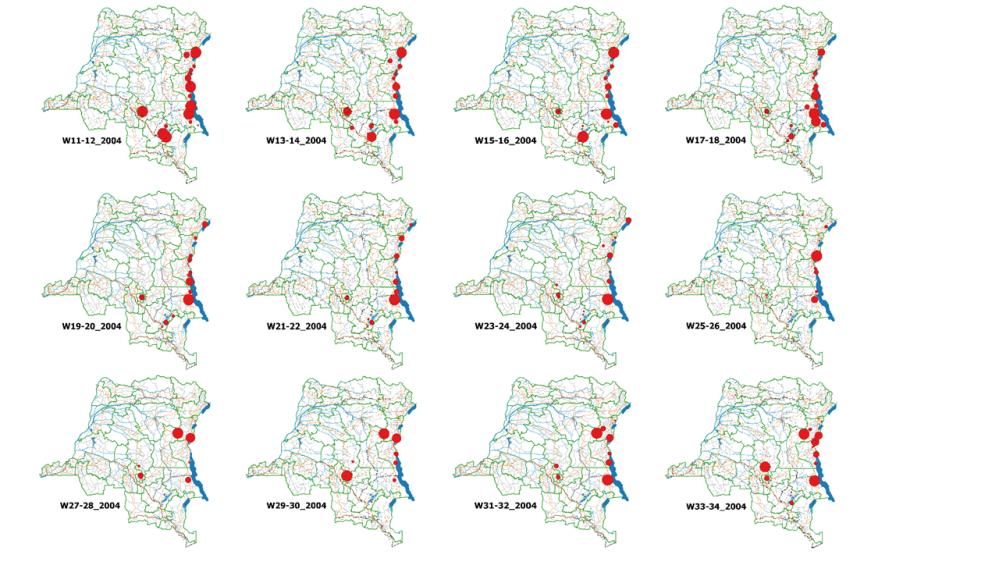

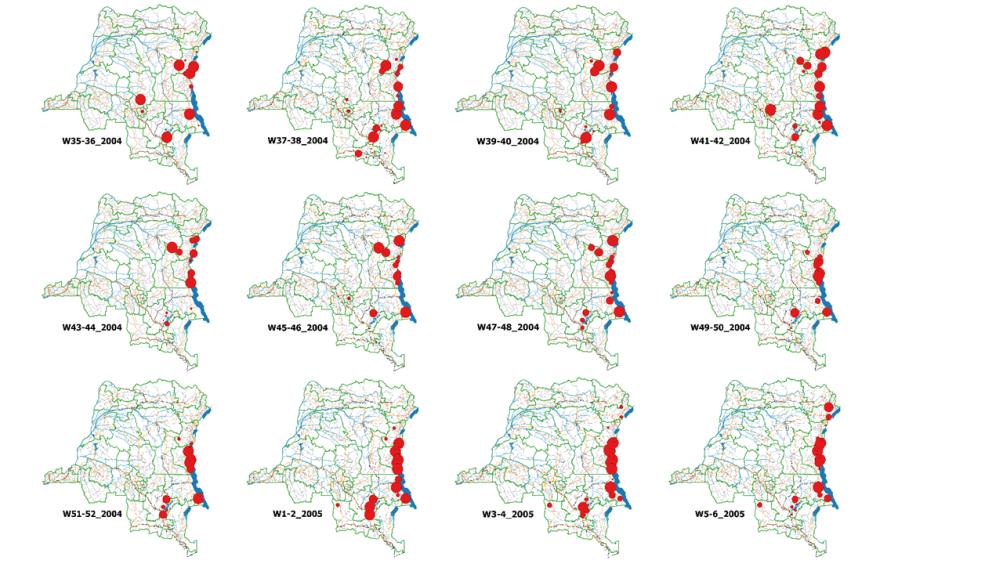

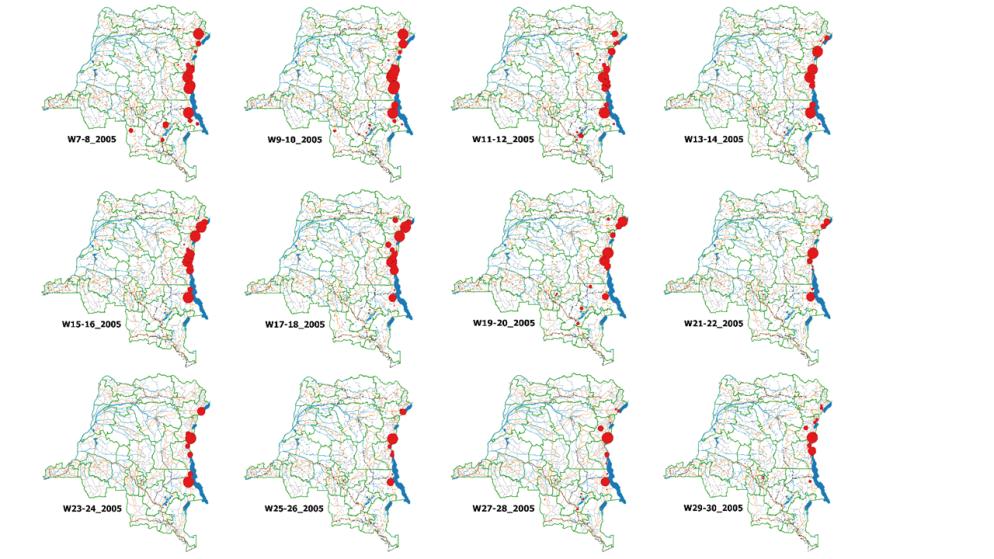

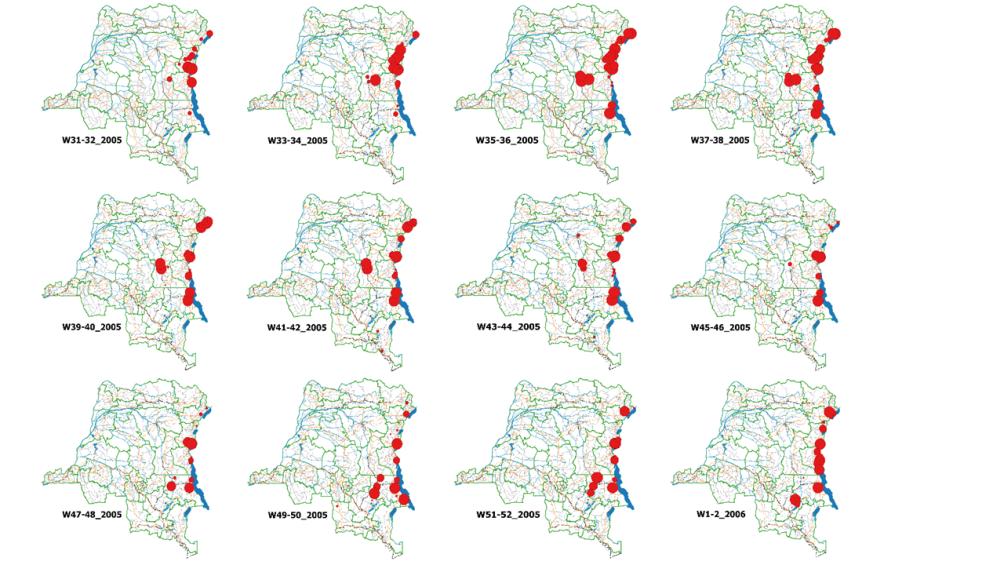

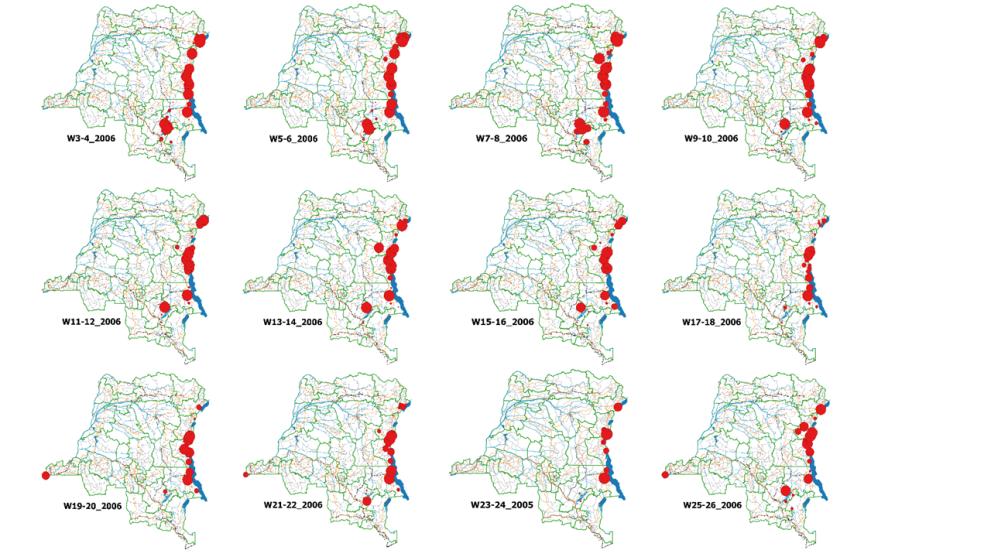

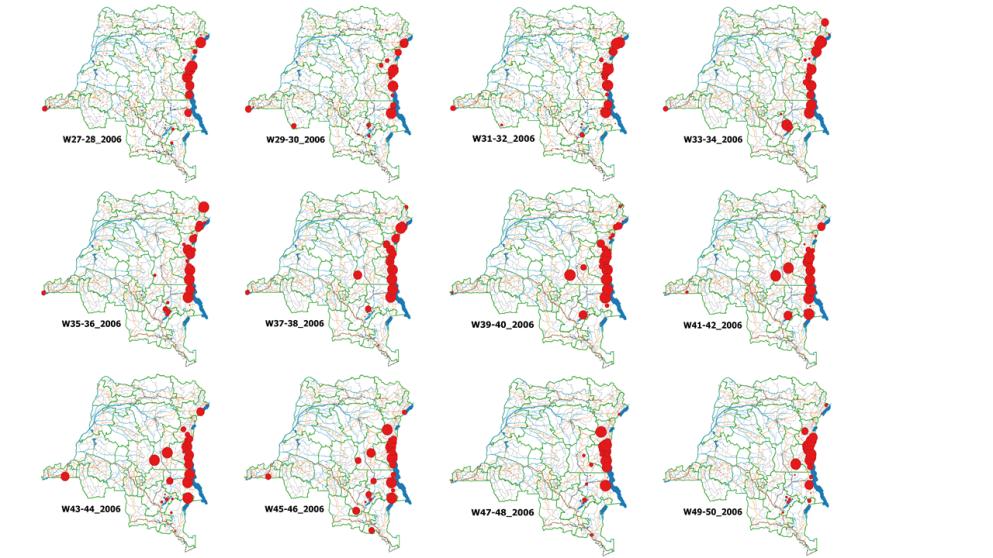

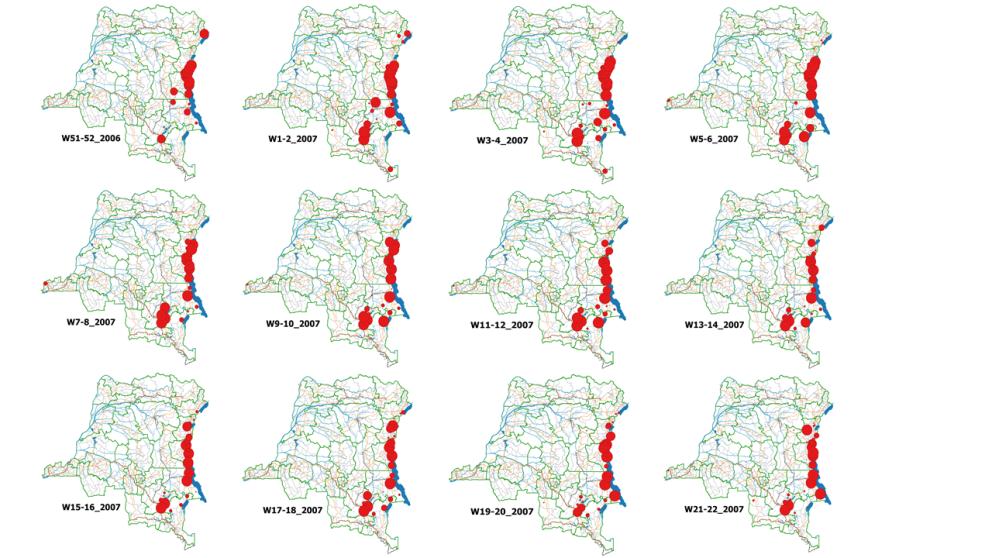

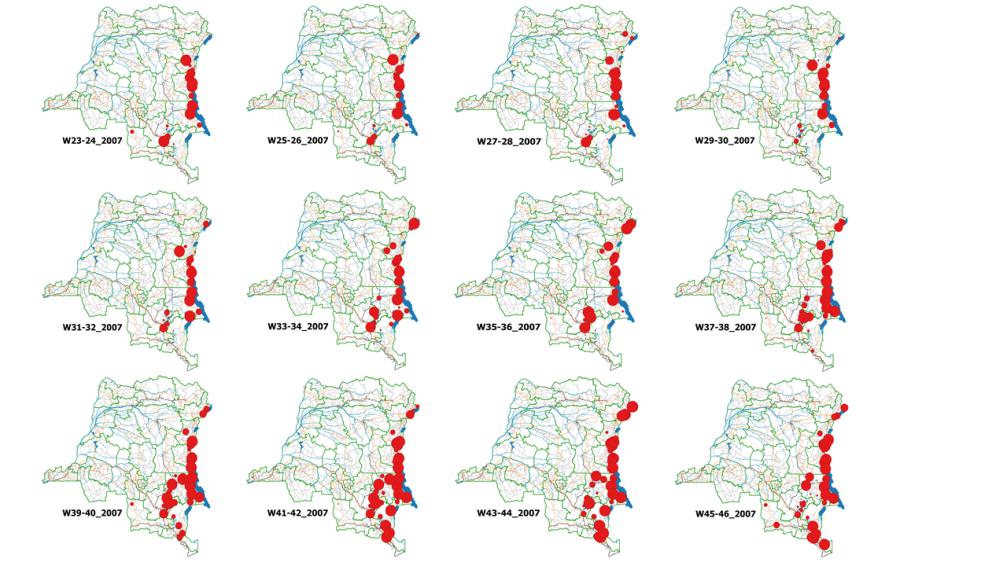

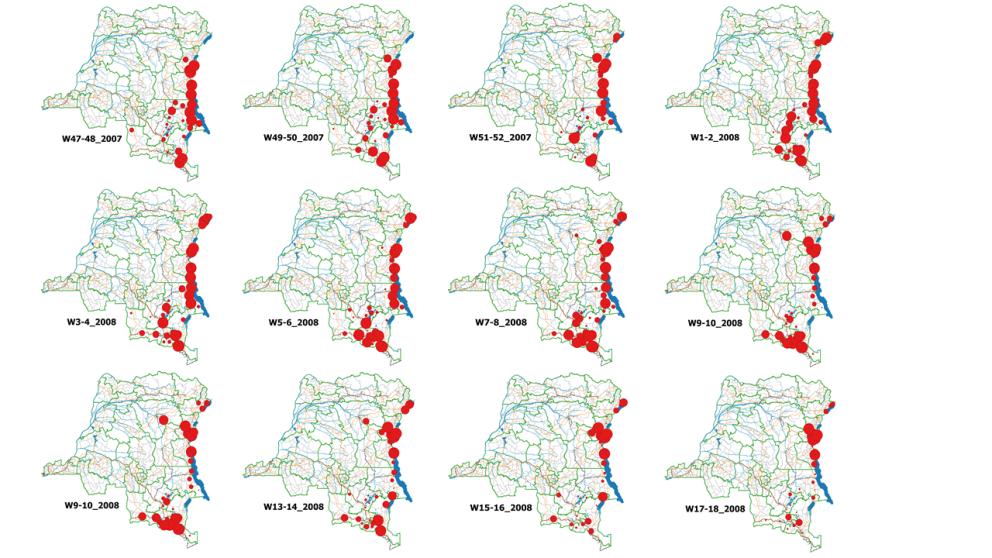

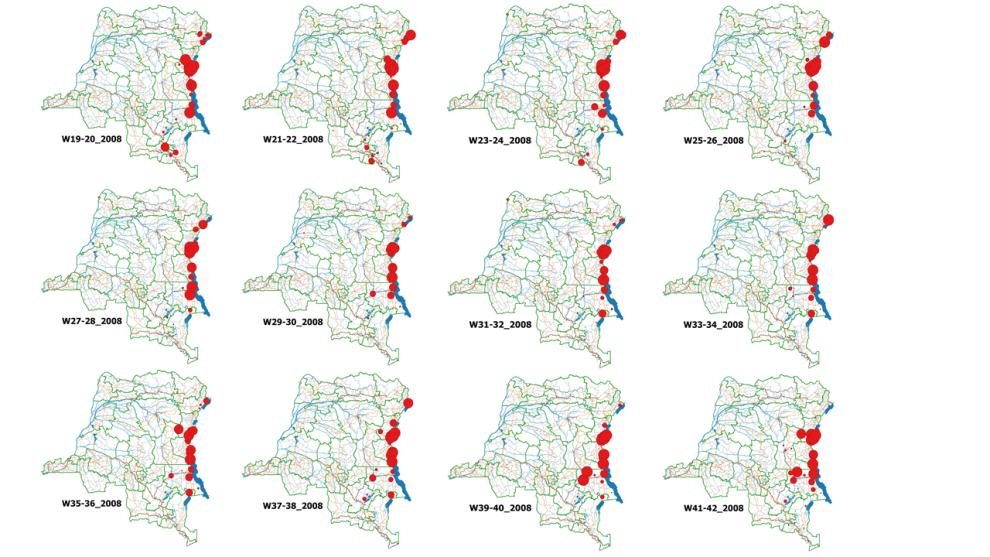

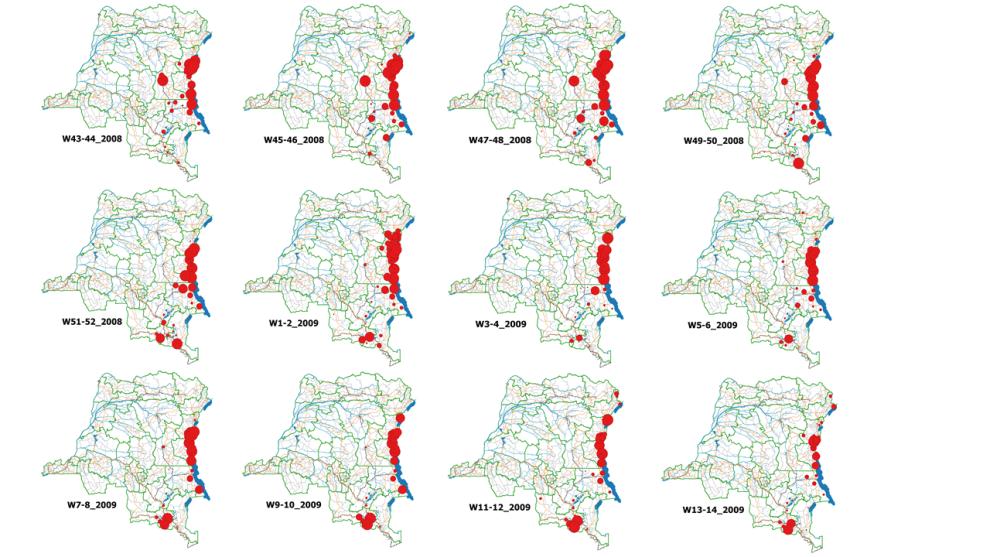

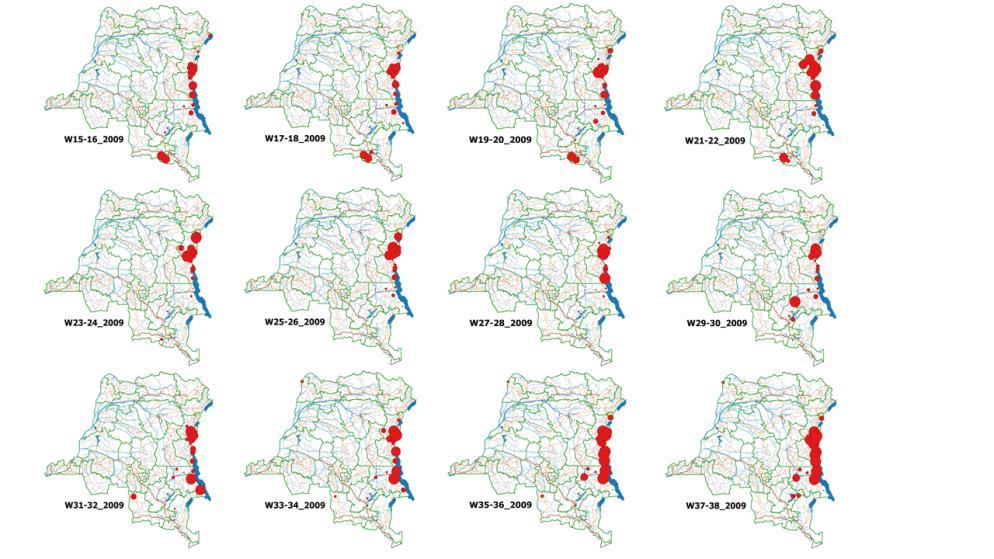

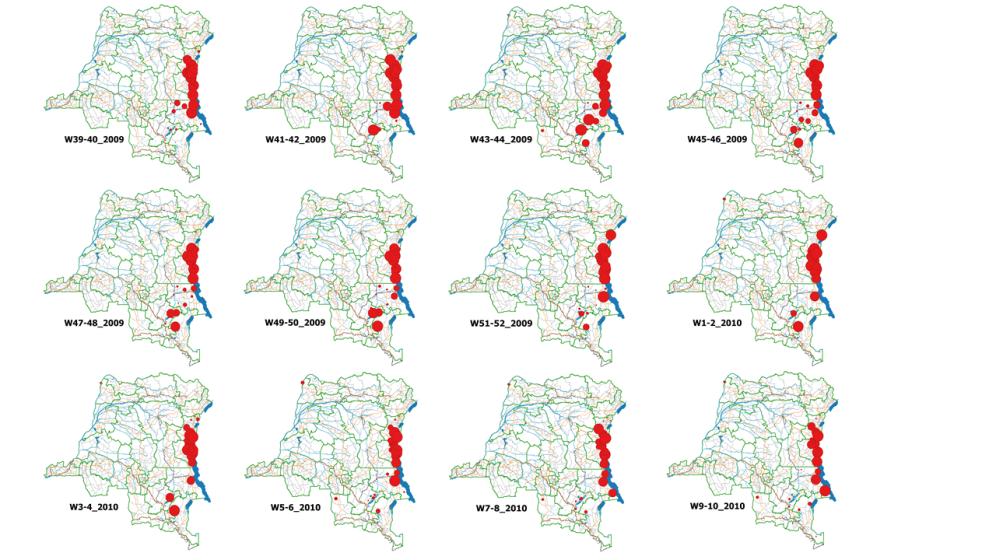

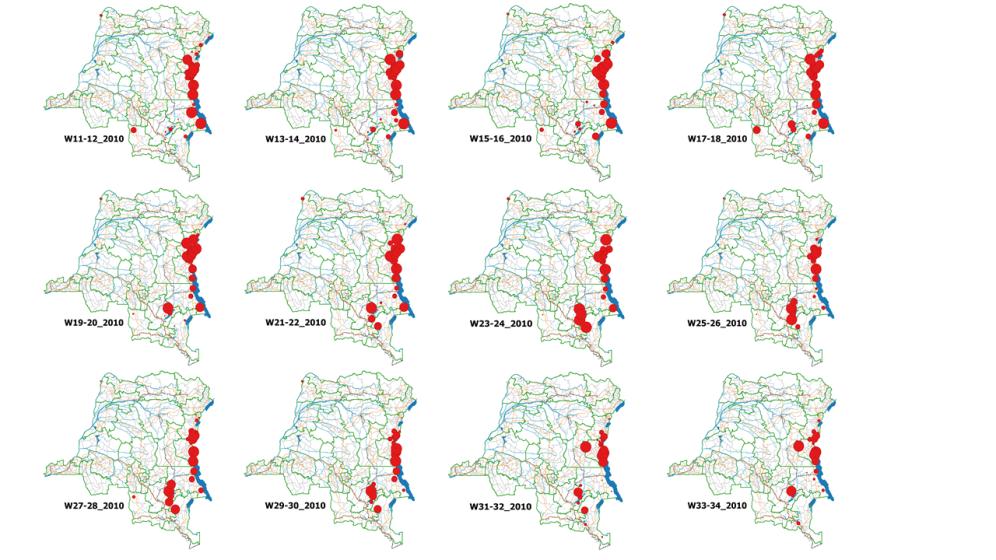

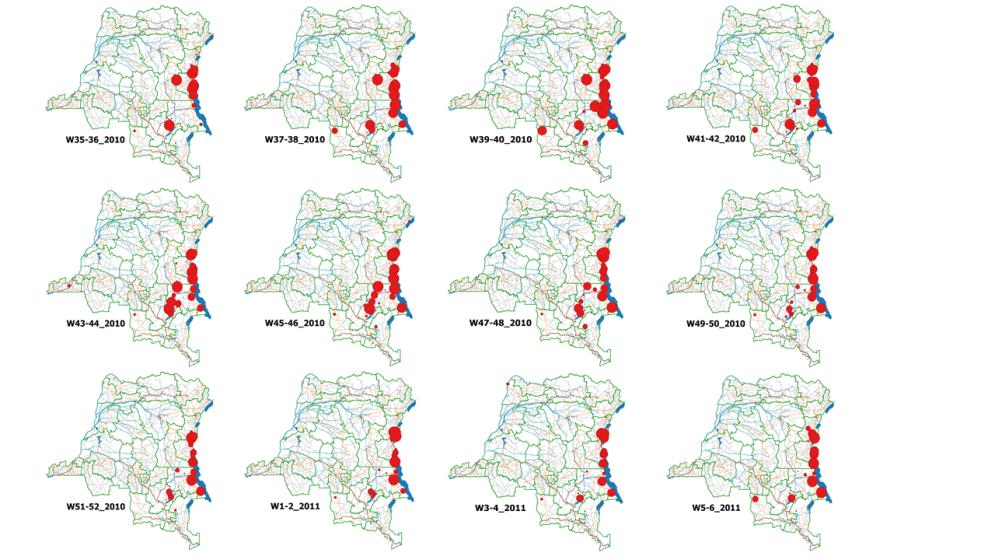

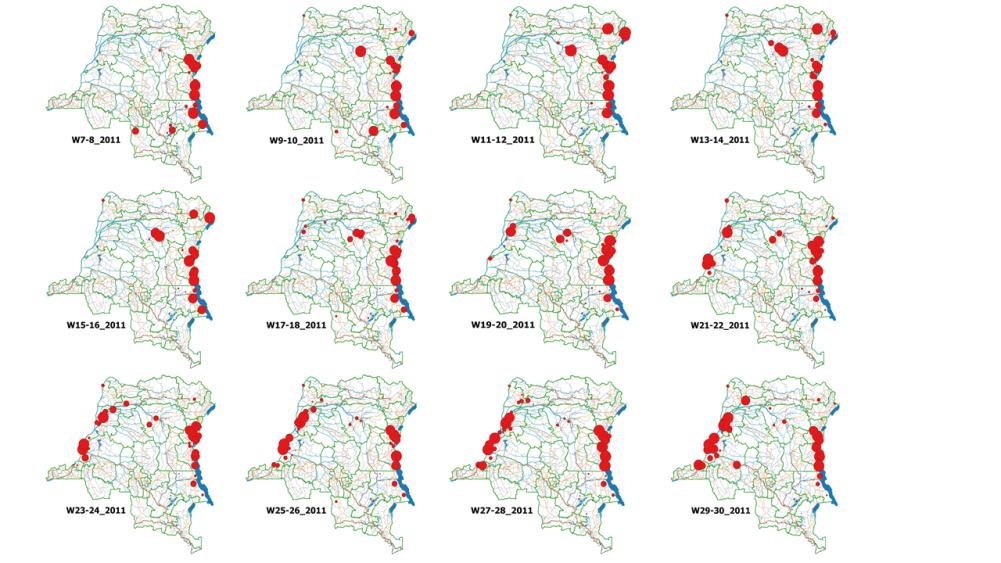

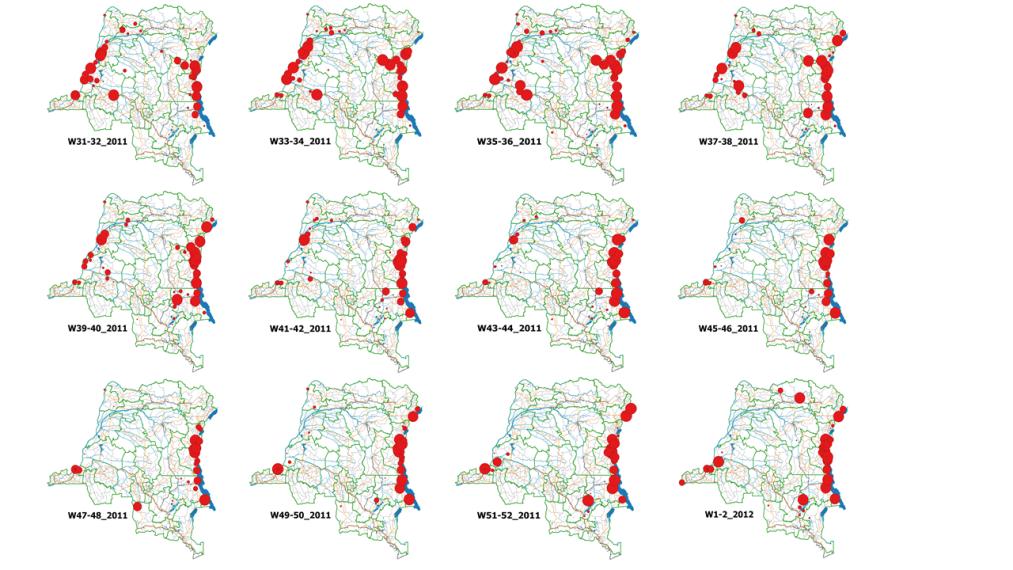

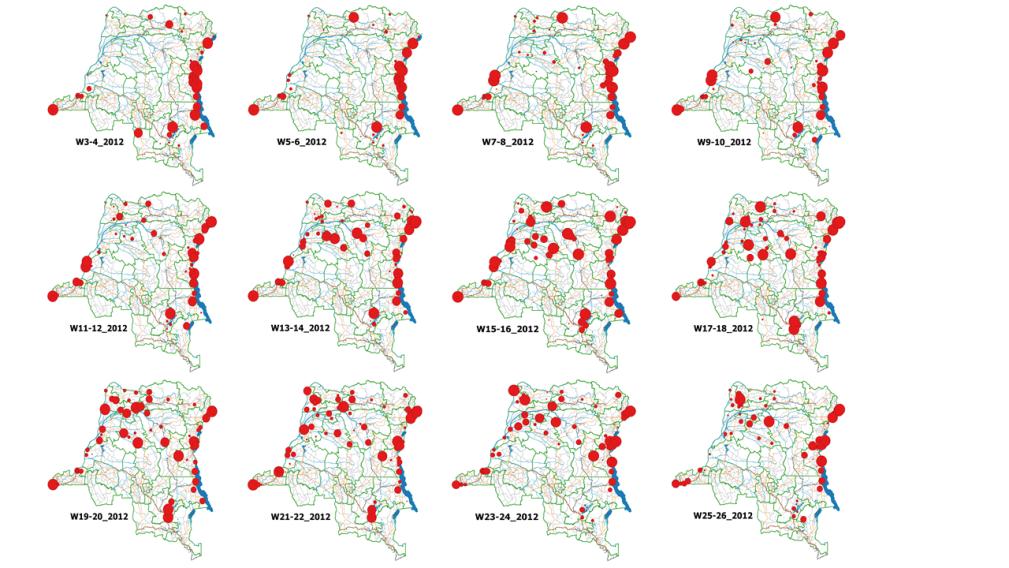

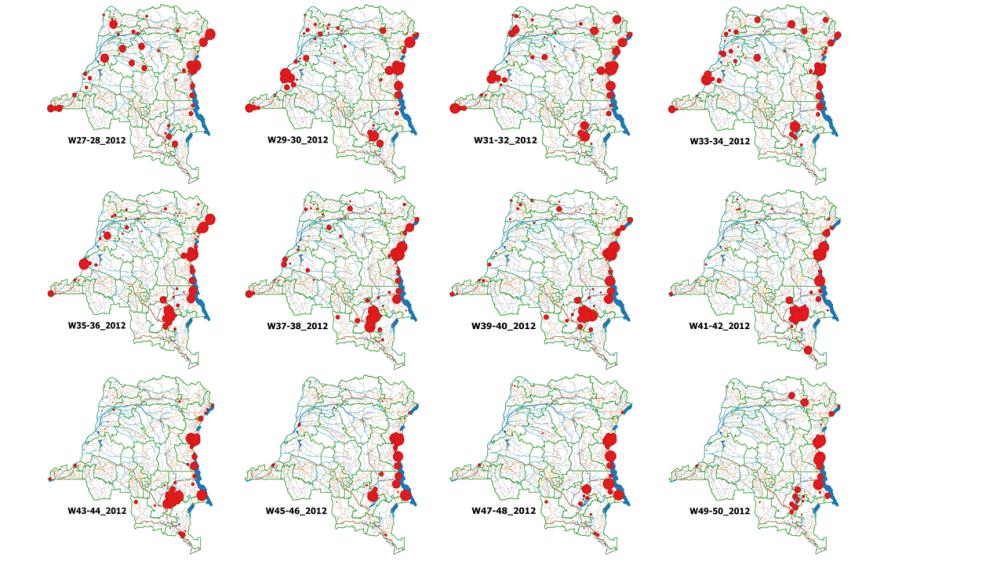

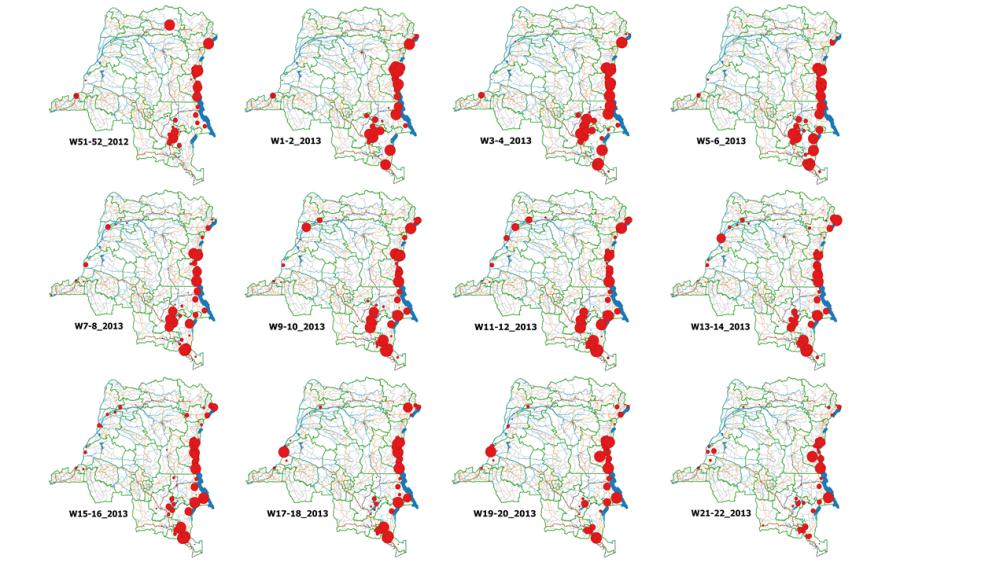

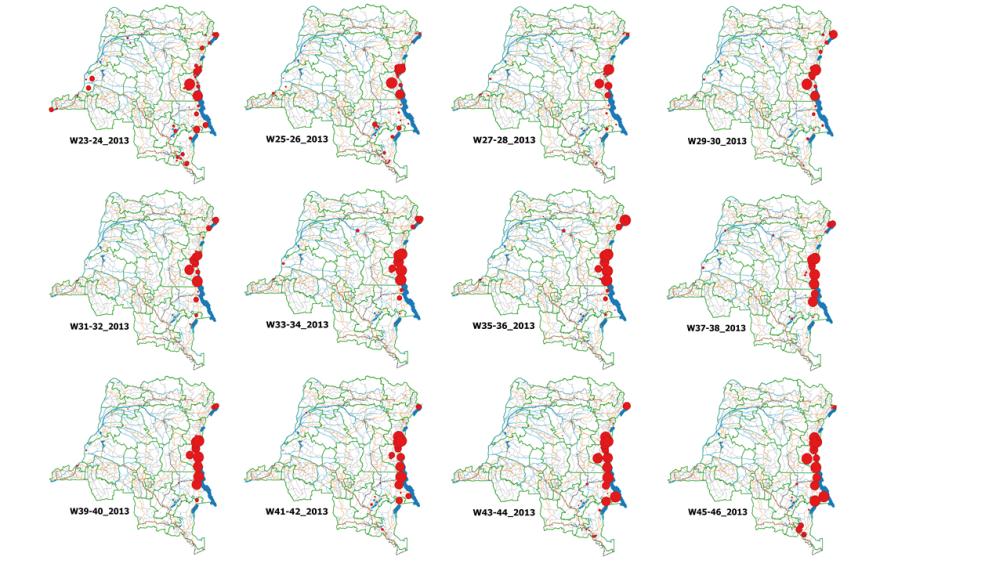

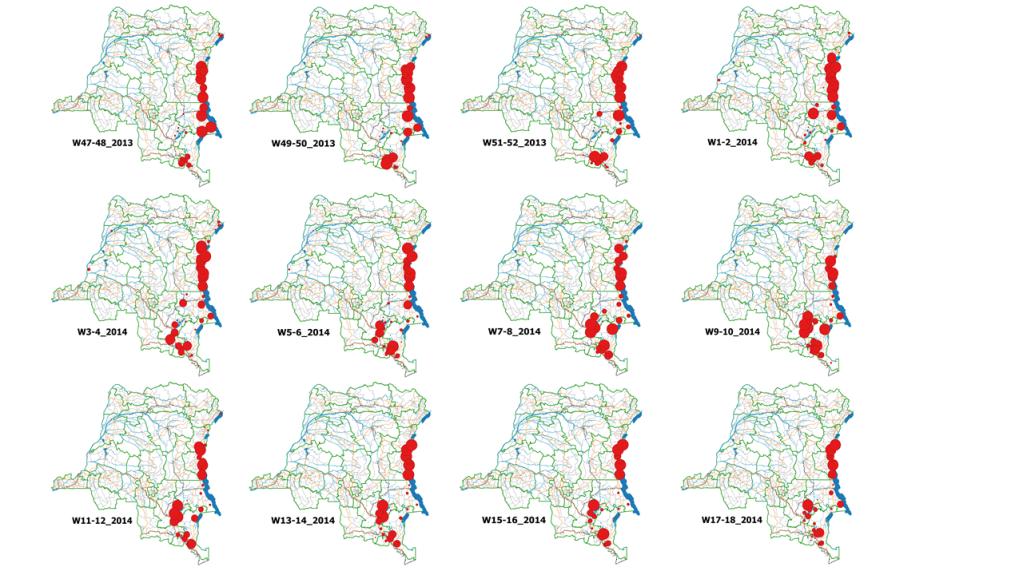

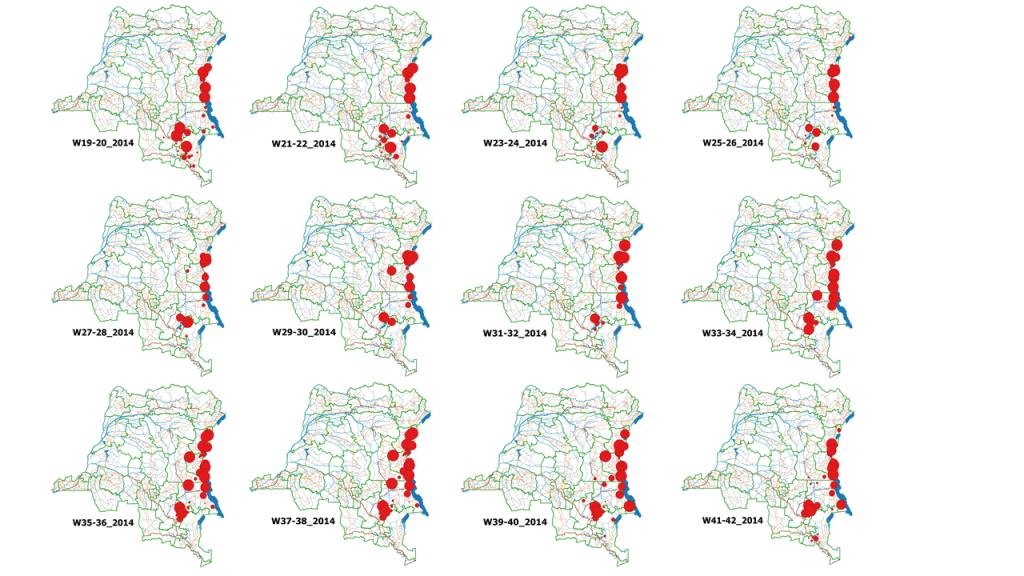

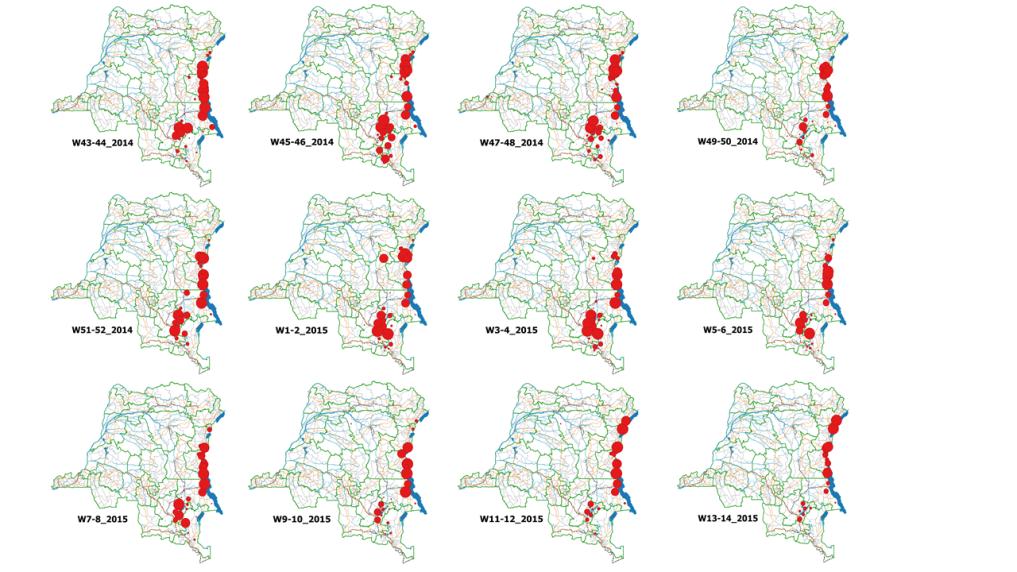

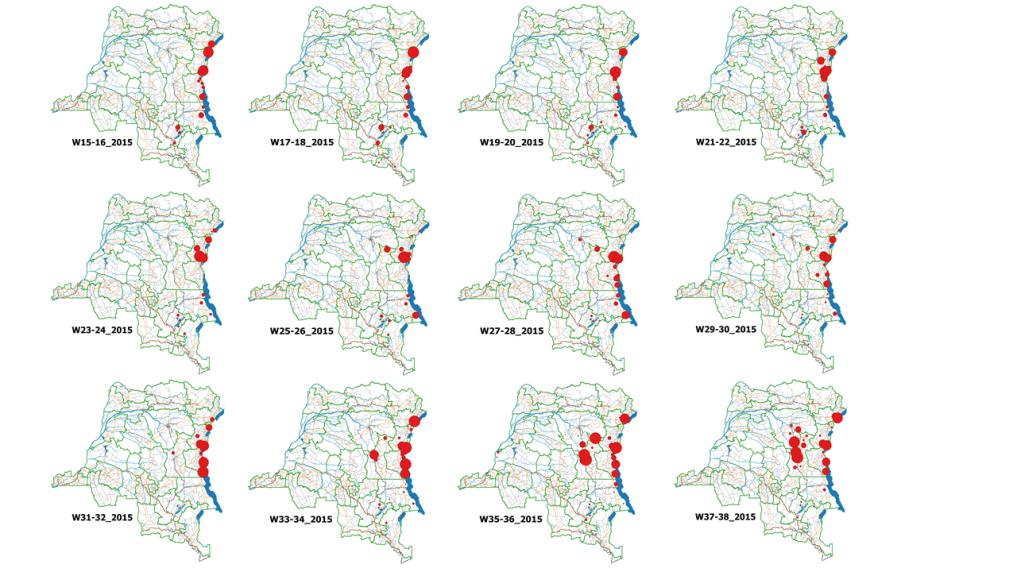

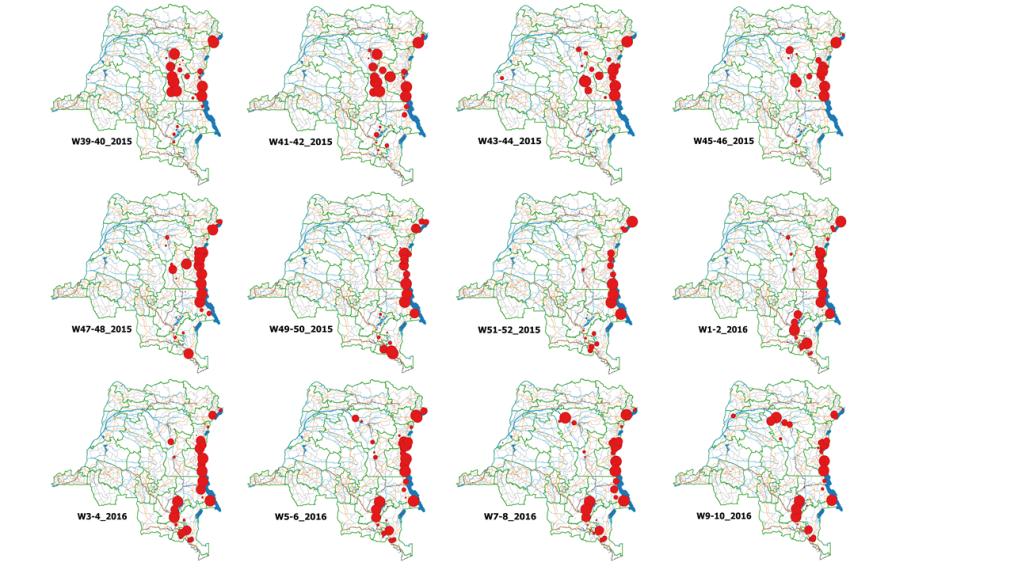

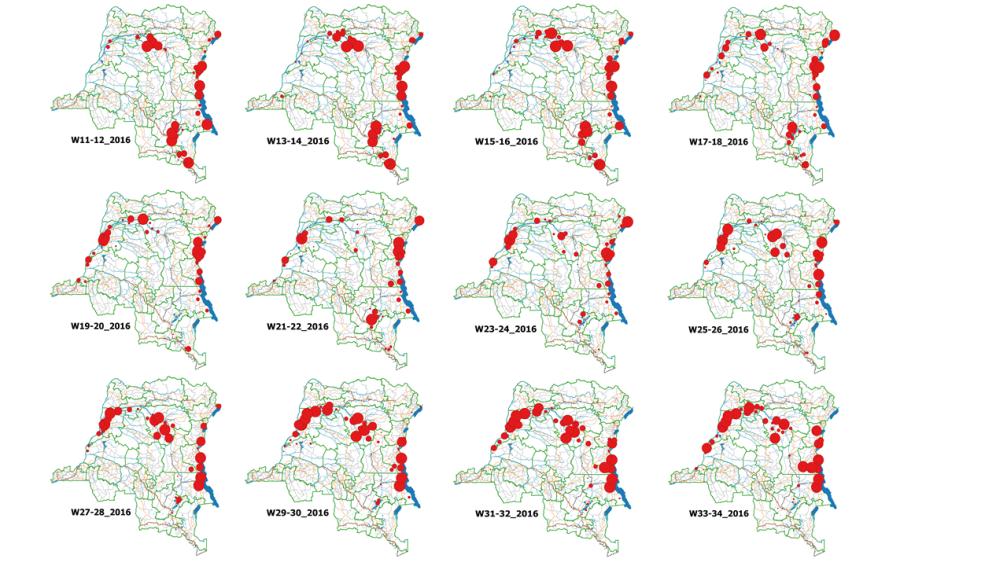

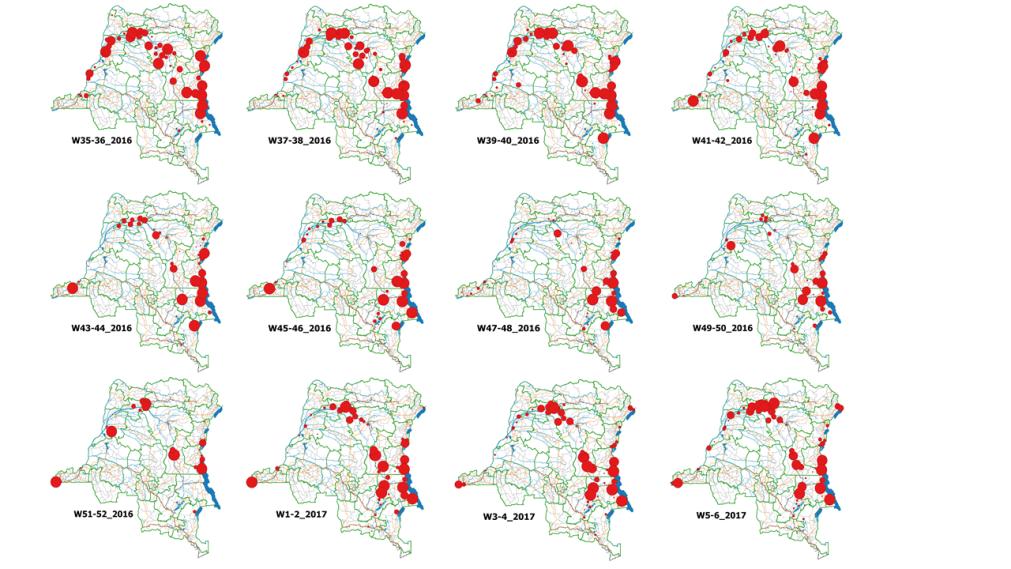

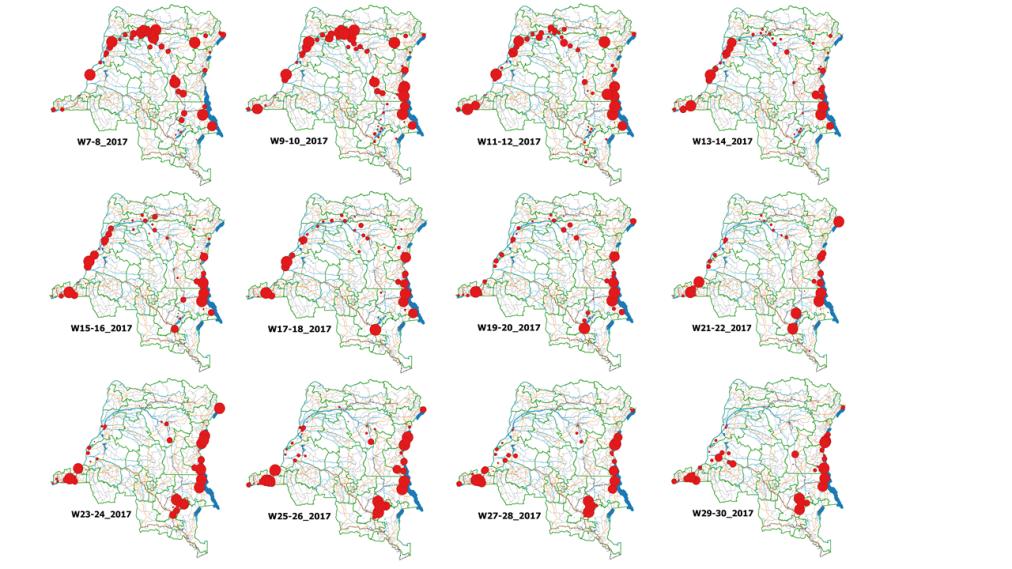


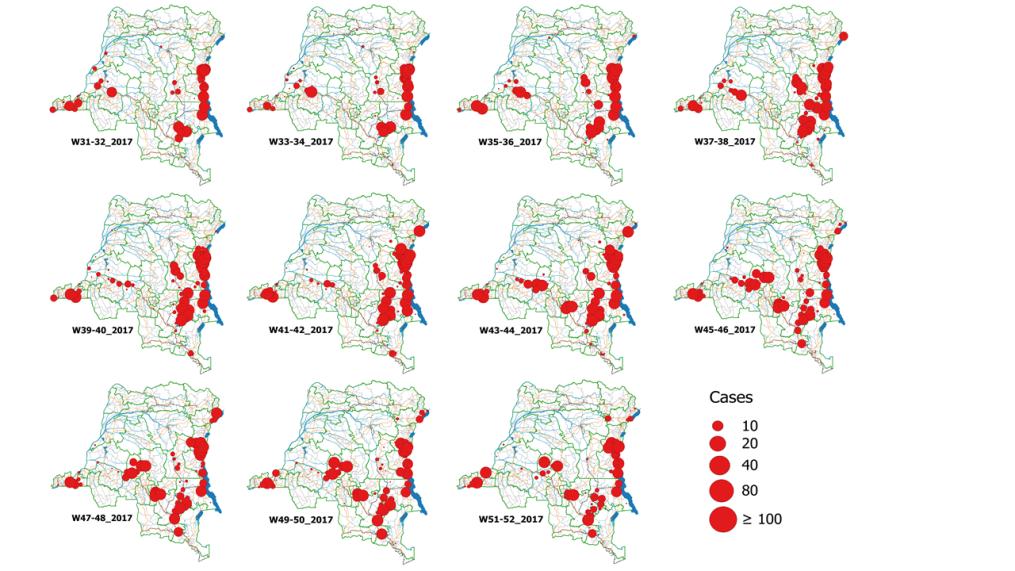

Supplement: S1 Fig — Republished from [30] under a CC BY license, with permission from [Claire Halleux], original copyright [2021]. (DOCX) [file pone.0263160.s001.docx]
