# Supplementary material for: Modalities and preferred routes of geographic spread of cholera from endemic areas in eastern Democratic Republic of the Congo
Source: PLoS One. 2022 Feb 7;17(2):e0263160. doi: 10.1371/journal.pone.0263160 (PMC8820636; doi:10.1371/journal.pone.0263160)
Supplement: S1 Table — (DOCX) [file pone.0263160.s004.docx]

**S1 Table.** Spatiotemporal clusters of cholera cases, DRC, 2000.

| **Cluster number** | **Health zones** | **Start time** | **End time** | **Radius (km)** | **Observed cases** | **Expected cases** | ***p*** |
| --- | --- | --- | --- | --- | --- | --- | --- |
| 1 | Karisimbi, Goma, Nyiragongo, Kirotshe, Minova, Rutshuru, Birambizo, Katana, Bambo, Kitoyi, Idjwi, Rwanguba | Week 29 | Week 50 | 54.23 | 3185 | 1446.40 | 1.0x10^-17^ |
| 2 | Musienene, Alimbongo, Biena, Manguredjipa, Kayna, Butembo, Lubero | Week 17 | Week 22 | 62.52 | 1121 | 281.31 | 1.0x10^-17^ |
| 3 | Pweto, Kasimba, Kiambi, Kilwa | Week 10 | Week 17 | 120.85 | 1218 | 465.47 | 1.0x10^-17^ |
| 4 | Kaziba, Mwana, Nyangezi, Mubumbano, Nyatende, Walungu, Lemera, Bagira Kasha, Kadutu | Week 48 | Week 52 | 34.89 | 576 | 132.89 | 1.0x10^-17^ |
| 5 | Kamango, Oicha, Boga, Mutwanga, Kalunguta, Beni, Komanda, Gethy, Mabalako, Kyondo | Week 42 | Week 47 | 76.23 | 245 | 39.73 | 1.0x10^-17^ |
| 6 | Uvira | Week 23 | Week 27 | 0 | 507 | 167.11 | 1.0x10^-17^ |
| 7 | Boko Kivulu, Mbanza Ngungu, Kisantu, Kwilu Ngongo, Kimpangu, Gombe Matadi, Sona Bata, Ngidinga, Nselo, Kimpese, Massa, Mont Ngafula I, Mont Ngafula II, Selembao, Kisenso, Kimbanseke, Biyela, Lemba, Binza Météo, Kikimi, Nsele, Ndjili, Makala, Bumbu, Kingasani, Ngaba, Matete, Ngiri Ngiri, Binza Ozone, Kintambo, Kalamu I, Bandalungwa, Limeté, Masina I, Kasa Vubu, Kokolo, Kimvula, Kalamu II, Lingwala, Kinshasa, Police, Barumbu, Kingabwa, Masina II | Week 1 | Week 8 | 117.10 | 366 | 113.99 | 1.0x10^-17^ |
| 8 | Mokala | Week 24 | Week 28 | 0 | 97 | 11.14 | 1.0x10^-17^ |
| 9 | Walikale, Itebero, Kibua, Punia, Pinga, Lubutu, Masisi | Week 14 | Week 16 | 111.76 | 418 | 174.00 | 1.0x10^-17^ |
| 10 | Kapemba, Kenya, Lubumbashi, Kamalondo, Katuba | Week 14 | Week 22 | 27.15 | 174 | 41.03 | 1.0x10^-17^ |
| 11 | Watsa | Week 10 | Week 10 | 0 | 40 | 1.16 | 1.0x10^-17^ |
| 12 | Bandjau, Tandembele, Ntondo, Lukolela, Nioki, Inongo | Week 40 | Week 46 | 102.54 | 64 | 5.26 | 1.0x10^-17^ |
| 13 | Ingende, Iboko, Bikoro, Mbandaka, Bolenge, Wangata, Pendjwa, Lotumbe, Bolomba | Week 19 | Week 27 | 93.17 | 230 | 77.52 | 1.0x10^-17^ |
| 14 | Kwamouth, Bandundu, Kikongo, Bolobo | Week 12 | Week 15 | 102.86 | 81 | 11.87 | 1.0x10^-17^ |
| 15 | Tchomia | Week 2 | Week 10 | 0 | 249 | 112.92 | 1.0x10^-17^ |
| 16 | Mawuya | Week 23 | Week 23 | 0 | 20 | 0.82 | 1.0x10^-17^ |
| 17 | Kaniama | Week 16 | Week 21 | 0 | 50 | 11.30 | 1.0x10^-14^ |
| 18 | Lubunga | Week 1 | Week 6 | 0 | 29 | 3.78 | 1.0x10^-13^ |
| 19 | Mukedi | Week 12 | Week 12 | 0 | 5 | 0.14 | 0.0013 |
| 20 | Kinkonzi, Kizu, Tshela, Vaku, Seke Banza, Kibunzi, Kuimba, Kangu, Inga, Lukula, Luozi | Week 9 | Week 11 | 82.63 | 7 | 0.64 | 0.024 |
| 21 | Kasaji | Week 6 | Week 7 | 0 | 4 | 0.12 | 0.036 |
