# Supplementary material for: Modalities and preferred routes of geographic spread of cholera from endemic areas in eastern Democratic Republic of the Congo
Source: PLoS One. 2022 Feb 7;17(2):e0263160. doi: 10.1371/journal.pone.0263160 (PMC8820636; doi:10.1371/journal.pone.0263160)
Supplement: S2 Table — (DOCX) [file pone.0263160.s005.docx]

**S2 Table.** Spatiotemporal clusters of cholera cases, DRC, 2001.

| **Cluster number** | **Health zones** | **Start time** | **End time** | **Radius (km)** | **Observed cases** | **Expected cases** | ***p*** |
| --- | --- | --- | --- | --- | --- | --- | --- |
| 1 | Kabalo, Ankoro | Week 41 | Week 47 | 79.68 | 1503 | 368.78 | 1.0x10^-17^ |
| 2 | Kayna | Week 6 | Week 10 | 0 | 961 | 222.46 | 1.0x10^-17^ |
| 3 | Kongolo | Week 29 | Week 39 | 0 | 484 | 52.38 | 1.0x10^-17^ |
| 4 | Lubumbashi | Week 11 | Week 11 | 0 | 166 | 12.14 | 1.0x10^-17^ |
| 5 | Kasaji | Week 50 | Week 52 | 0 | 184 | 17.45 | 1.0x10^-17^ |
| 6 | Karisimbi, Goma, Nyiragongo, Kirotshe, Minova, Rutshuru, Birambizo, Katana, Bambo, Kitoyi, Mweso, Rwanguba | Week 10 | Week 28 | 55.73 | 1050 | 485.22 | 1.0x10^-17^ |
| 7 | Katuba, Kisanga, Kenya, Kamalondo, Mubunda, Tshamilemba, Ruashi | Week 1 | Week 6 | 26.11 | 391 | 118.04 | 1.0x10^-17^ |
| 8 | Pweto, Kasimba, Kiambi, Kilwa | Week 1 | Week 5 | 120.85 | 127 | 28.53 | 1.0x10^-17^ |
| 9 | Uvira | Week 38 | Week 42 | 0 | 265 | 105.51 | 1.0x10^-17^ |
| 10 | Walikale, Itebero, Kibua, Punia, Pinga | Week 23 | Week 29 | 91.89 | 68 | 8.16 | 1.0x10^-17^ |
| 11 | Mongbwalu, Nyakunde, Kilo, Damasi, Bambu, Mangala, Kambala, Nizi, Rwampara, Biringi, Fataki, Bunia, Aungba, Lita, Lolwa, Rimba, Mandima, Tchomia, Watsa | Week 8 | Week 8 | 92.04 | 30 | 1.39 | 1.0x10^-17^ |
| 12 | Mwana, Mubumbano, Kaziba, Mwenga, Walungu, Nyangezi, Nyatende, Kaniola, Bagira Kasha, Lemera, Kadutu, Ibanda, Haut Plateau, Kabare | Week 48 | Week 50 | 52.36 | 120 | 35.61 | 1.0x10^-17^ |
| 13 | Bunkeya, Kambove, Kikula | Week 47 | Week 52 | 54.82 | 71 | 14.08 | 1.0x10^-17^ |
| 14 | Adi, Laybo, Ariwara, Adia, Aba, Aru | Week 15 | Week 15 | 61.23 | 10 | 0.10 | 2.2x10^-14^ |
| 15 | Bukama | Week 3 | Week 3 | 0 | 12 | 0.40 | 2.2x10^-11^ |
| 16 | Kamango, Oicha, Boga, Mutwanga | Week 13 | Week 23 | 39.25 | 42 | 11.22 | 3.1x10^-09^ |
| 17 | Kalima | Week 17 | Week 17 | 0 | 6 | 0.042 | 8.4x10^-09^ |
| 18 | Mont Ngafula I | Week 9 | Week 9 | 0 | 12 | 0.70 | 2.3x10^-08^ |
| 19 | Ngiri Ngiri | Week 30 | Week 30 | 0 | 6 | 0.050 | 2.4x10^-08^ |
| 20 | Kindu | Week 41 | Week 43 | 0 | 21 | 5.14 | 0.0004 |
| 21 | Nioki | Week 46 | Week 46 | 0 | 4 | 0.14 | 0.035 |
