# Supplementary material for: Modalities and preferred routes of geographic spread of cholera from endemic areas in eastern Democratic Republic of the Congo
Source: PLoS One. 2022 Feb 7;17(2):e0263160. doi: 10.1371/journal.pone.0263160 (PMC8820636; doi:10.1371/journal.pone.0263160)
Supplement: S3 Table — (DOCX) [file pone.0263160.s006.docx]

**S3 Table.** Spatiotemporal clusters of cholera cases, DRC, 2002.

| **Cluster number** | **Health zones** | **Start time** | **End time** | **Radius (km)** | **Observed cases** | **Expected cases** | ***p*** |
| --- | --- | --- | --- | --- | --- | --- | --- |
| 1 | Pinga | Week 28 | Week 31 | 0 | 845 | 76.15 | 1.0x10^-17^ |
| 2 | Malemba Nkulu | Week 33 | Week 38 | 0 | 2560 | 862.46 | 1.0x10^-17^ |
| 3 | Kongolo | Week 39 | Week 46 | 0 | 699 | 101.10 | 1.0x10^-17^ |
| 4 | Lukafu, Kikula, Kasenga, Bunkeya, Kapolobwe, Kafubu, Kambove, Lubumbashi, Tshamilemba, Vangu, Kowe, Ruashi, Likasi | Week 1 | Week 11 | 105.48 | 2288 | 909.51 | 1.0x10^-17^ |
| 5 | Fizi | Week 1 | Week 4 | 0 | 591 | 77.34 | 1.0x10^-17^ |
| 6 | Kyondo | Week 43 | Week 51 | 0 | 380 | 38.93 | 1.0x10^-17^ |
| 7 | Nyangezi, Nyatende, Kaziba, Bagira Kasha, Kadutu, Ibanda, Walungu, Mubumbano, Lemera, Mwana, Kabare, Ruzizi, Haut Plateau, Kaniola, Mwenga, Idjwi, Kalonge, Miti Murhesa, Katana, Bunyakiri, Uvira, Kahele, Minova, Itombwe, Kamituga, Nundu, Kitoyi, Goma, Karisimbi, Mulungu, Kirotshe | Week 19 | Week 27 | 114.41 | 1033 | 356.62 | 1.0x10^-17^ |
| 8 | Kalemie | Week 27 | Week 29 | 0 | 648 | 163.71 | 1.0x10^-17^ |
| 9 | Tshilenge, Tshitenge, Bonzola, Lubilanji, Dibindi, Lukelenge, Kansele | Week 42 | Week 43 | 23.62 | 180 | 7.83 | 1.0x10^-17^ |
| 10 | Ankoro, Kinkondja | Week 7 | Week 18 | 79.05 | 3730 | 2420.55 | 1.0x10^-17^ |
| 11 | Bukama | Week 5 | Week 6 | 0 | 431 | 100.05 | 1.0x10^-17^ |
| 12 | Lualaba, Dilala, Manika | Week 8 | Week 17 | 66.39 | 644 | 217.90 | 1.0x10^-17^ |
| 13 | Bunia | Week 34 | Week 37 | 0 | 348 | 83.33 | 1.0x10^-17^ |
| 14 | Lolwa, Komanda, Mandima, Kilo, Rwampara, Boga, Gethy, Mongbwalu, Bambu, Nizi, Oicha, Nyakunde, Mambasa, Damasi, Kamango, Tchomia | Week 17 | Week 22 | 101.47 | 295 | 62.07 | 1.0x10^-17^ |
| 15 | Lubutu, Obokote, Opienge, Walikale | Week 23 | Week 44 | 103.69 | 673 | 291.09 | 1.0x10^-17^ |
| 16 | Kitenge | Week 10 | Week 14 | 0 | 144 | 24.47 | 1.0x10^-17^ |
| 17 | Songa, Kamina Base, Kamina, Kabongo, Kayamba, Kabondo Dianda | Week 48 | Week 51 | 104.14 | 138 | 23.27 | 1.0x10^-17^ |
| 18 | Kasaji | Week 1 | Week 10 | 0 | 519 | 247.78 | 1.0x10^-17^ |
| 19 | Moba | Week 40 | Week 52 | 0 | 286 | 107.26 | 1.0x10^-17^ |
| 20 | Doruma | Week 5 | Week 8 | 0 | 126 | 27.29 | 1.0x10^-17^ |
| 21 | Kaniama | Week 8 | Week 11 | 0 | 165 | 46.18 | 1.0x10^-17^ |
| 22 | Mufunga Sampwe | Week 42 | Week 45 | 0 | 235 | 87.90 | 1.0x10^-17^ |
| 23 | Sakania | Week 17 | Week 19 | 0 | 21 | 1.06 | 1.0x10^-17^ |
| 24 | Kapanga | Week 36 | Week 43 | 0 | 60 | 16.38 | 1.0x10^-13^ |
| 25 | Limeté, Kalamu I, Kalamu II, Ngaba, Kasa Vubu, Kingabwa, Barumbu, Matete, Makala, Ngiri Ngiri, Kinshasa, Bumbu, Lingwala, Lemba | Week 47 | Week 47 | 5.27 | 11 | 0.19 | 3.0x10^-13^ |
| 26 | Katako Kombe | Week 25 | Week 25 | 0 | 11 | 0.87 | 9.0x10^-06^ |
| 27 | Dikungu Tshumbe, Minga, Wembo Nyama, Vanga Kete, Pania Mutombo, Tumba, Lusambo, Djalo Djeka, Tshofa, Lodja | Week 6 | Week 7 | 120.99 | 15 | 2.81 | 0.0016 |
| 28 | Zongo | Week 1 | Week 4 | 0 | 12 | 1.87 | 0.0036 |
