# Supplementary material for: Modalities and preferred routes of geographic spread of cholera from endemic areas in eastern Democratic Republic of the Congo
Source: PLoS One. 2022 Feb 7;17(2):e0263160. doi: 10.1371/journal.pone.0263160 (PMC8820636; doi:10.1371/journal.pone.0263160)
Supplement: S4 Table — (DOCX) [file pone.0263160.s007.docx]

**S4 Table.** Spatiotemporal clusters of cholera cases, DRC, 2003.

| **Cluster number** | **Health zones** | **Start time** | **End time** | **Radius (km)** | **Observed cases** | **Expected cases** | ***p*** |
| --- | --- | --- | --- | --- | --- | --- | --- |
| 1 | Lubumbashi, Kapemba, Kafubu, Kamalondo, Kenya, Tshamilemba, Katuba, Vangu, Ruashi | Week 9 | Week 23 | 48.19 | 2189 | 809.39 | 1.0x10^-17^ |
| 2 | Nzaba, Mpokolo, Bimpemba, Diulu, Kansele, Bonzola, Lubilanji, Dibindi, Muya, Lukelenge, Tshitshimbi, Tshitenge, Miabi, Mukumbi, Tshilenge, Tshilundu, Bibanga, Kasansa, Kabeya Kamuanga | Week 29 | Week 33 | 45.60 | 676 | 119.78 | 1.0x10^-17^ |
| 3 | Kabalo, Ankoro, Mbulala, Kongolo, Manono | Week 40 | Week 43 | 105.29 | 495 | 61.33 | 1.0x10^-17^ |
| 4 | Rwanguba | Week 33 | Week 38 | 0 | 363 | 34.54 | 1.0x10^-17^ |
| 5 | Kaniama | Week 7 | Week 23 | 0 | 1519 | 610.21 | 1.0x10^-17^ |
| 6 | Haut Plateau, Uvira, Lemera, Ruzizi, Kaziba, Nundu, Itombwe, Mwana, Mwenga, Nyangezi, Mubumbano, Nyatende, Walungu, Bagira Kasha, Kadutu, Ibanda, Minembwe, Kamituga, Kaniola, Kabare, Kitutu, Kalonge, Miti Murhesa, Idjwi, Kimbi Lulenge, Fizi, Katana | Week 37 | Week 52 | 121.23 | 1959 | 934.94 | 1.0x10^-17^ |
| 7 | Kalemie, Kasimba | Week 43 | Week 49 | 85.08 | 752 | 212.61 | 1.0x10^-17^ |
| 8 | Manguredjipa | Week 13 | Week 13 | 0 | 81 | 1.85 | 1.0x10^-17^ |
| 9 | Mukanga | Week 38 | Week 40 | 0 | 192 | 32.41 | 1.0x10^-17^ |
| 10 | Kitenge, Kalonda Est, Kayamba, Kabongo | Week 50 | Week 52 | 84.83 | 79 | 3.01 | 1.0x10^-17^ |
| 11 | Kinkondja | Week 33 | Week 38 | 0 | 316 | 98.08 | 1.0x10^-17^ |
| 12 | Rwampara, Nizi, Gethy, Bambu, Tchomia, Bunia, Kilo, Boga, Komanda, Lita, Mongbwalu, Lolwa, Mangala, Nyakunde, Fataki, Kamango, Drodro, Oicha, Damasi, Kambala, Jiba, Linga, Rimba, Rethy, Mandima, Logo, Beni | Week 5 | Week 8 | 117.09 | 189 | 43.63 | 1.0x10^-17^ |
| 13 | Kilwa | Week 22 | Week 23 | 0 | 51 | 2.69 | 1.0x10^-17^ |
| 14 | Bukama, Butumba, Lubudi, Kabondo Dianda | Week 1 | Week 5 | 83.83 | 539 | 275.31 | 1.0x10^-17^ |
| 15 | Moba | Week 39 | Week 42 | 0 | 141 | 35.43 | 1.0x10^-17^ |
| 16 | Kambove, Likasi, Fungurume, Bunkeya, Kapolobwe, Kikula | Week 14 | Week 16 | 60.46 | 76 | 10.21 | 1.0x10^-17^ |
| 17 | Mufunga Sampwe | Week 1 | Week 6 | 0 | 119 | 28.28 | 1.0x10^-17^ |
| 18 | Lualaba, Dilala, Manika | Week 24 | Week 24 | 66.39 | 19 | 0.19 | 1.0x10^-17^ |
| 19 | Walikale, Itebero, Kibua, Punia, Pinga | Week 22 | Week 24 | 91.89 | 61 | 10.45 | 1.0x10^-17^ |
| 20 | Ipamu | Week 12 | Week 12 | 0 | 12 | 0.31 | 1.8x10^-12^ |
| 21 | Kapanga | Week 41 | Week 46 | 0 | 19 | 2.75 | 6.0x10^-07^ |
| 22 | Kasaji | Week 1 | Week 9 | 0 | 68 | 30.03 | 1.3x10^-05^ |
| 23 | Sakania | Week 22 | Week 23 | 0 | 11 | 0.91 | 1.8x10^-05^ |
| 24 | Lukolela | Week 7 | Week 8 | 0 | 9 | 0.54 | 3.5x10^-05^ |
