# Supplementary material for: Modalities and preferred routes of geographic spread of cholera from endemic areas in eastern Democratic Republic of the Congo
Source: PLoS One. 2022 Feb 7;17(2):e0263160. doi: 10.1371/journal.pone.0263160 (PMC8820636; doi:10.1371/journal.pone.0263160)
Supplement: S5 Table — (DOCX) [file pone.0263160.s008.docx]

**S5 Table.** Spatiotemporal clusters of cholera cases, DRC, 2004.

| **Cluster number** | **Health zones** | **Start time** | **End time** | **Radius (km)** | **Observed cases** | **Expected cases** | ***p*** |
| --- | --- | --- | --- | --- | --- | --- | --- |
| 1 | Kibua, Masisi, Kitoyi, Mweso, Itebero, Pinga, Kirotshe, Walikale, Minova, Kahele, Birambizo, Bunyakiri, Miti Murhesa, Goma, Bambo, Kibirizi, Karisimbi, Kayna, Nyiragongo, Katana, Kalonge, Rutshuru | Week 25 | Week 36 | 109.65 | 1235 | 412.88 | 1.0x10^-17^ |
| 2 | Kalambayi Kabanga, Ngandajika, Lukashi lualu, Mulumba, Kanda Kanda, Kayamba, Kalonda Est, Bibanga, Tshilenge, Kabinda, Kasansa, Tshitenge, Luputa, Lukelenge, Mwene Ditu, Dibindi, Bonzola | Week 12 | Week 13 | 106.85 | 227 | 23.66 | 1.0x10^-17^ |
| 3 | Nundu, Uvira, Haut Plateau, Itombwe, Minembwe, Ruzizi, Lemera, Fizi, Mwenga, Kimbi Lulenge, Kaziba, Mwana, Nyangezi | Week 1 | Week 9 | 98.87 | 1376 | 672.29 | 1.0x10^-17^ |
| 4 | Opienge, Lubutu | Week 44 | Week 46 | 94.16 | 174 | 14.53 | 1.0x10^-17^ |
| 5 | Moba | Week 45 | Week 52 | 0 | 357 | 78.39 | 1.0x10^-17^ |
| 6 | Nyemba | Week 12 | Week 12 | 0 | 139 | 12.79 | 1.0x10^-17^ |
| 7 | Katwa | Week 41 | Week 48 | 0 | 233 | 45.88 | 1.0x10^-17^ |
| 8 | Lukonga, Bobozo, Kananga, Ndesha, Tshikaji, Katoka, Tshikula, Mikalayi, Mutoto, Demba, Bukonde, Katende, Bilonda, Dibaya, Ndekesha, Kabeya Kamuanga, Bena Leke, Lubondaie, Miabi, Tshilundu, Lubunga, Luebo, Tshibala, Mwetshi, Kamiji, Tshitshimbi, Mpokolo, Luiza, Mukumbi | Week 28 | Week 29 | 122.97 | 84 | 4.46 | 1.0x10^-17^ |
| 9 | Malemba Nkulu, Lwamba, x, x, Mukanga, Kabondo Dianda, Kinkondja, Butumba | Week 1 | Week 11 | 70.63 | 806 | 432.52 | 1.0x10^-17^ |
| 10 | Minga, Dikungu Tshumbe, Pania Mutombo, Tshofa, Wembo Nyama, Lusambo | Week 32 | Week 36 | 101.18 | 107 | 15.14 | 1.0x10^-17^ |
| 11 | Kasimba, Pweto, Kalemie | Week 13 | Week 24 | 85.08 | 548 | 280.74 | 1.0x10^-17^ |
| 12 | Shabunda, Lulingu, Kalima, Pangi, Mulungu, Kakole, Punia | Week 37 | Week 40 | 101.19 | 54 | 4.50 | 1.0x10^-17^ |
| 13 | Dilala, Mutshatsha, Kilela Balanda, Lualaba, Kinda, Lubudi | Week 38 | Week 38 | 119.73 | 28 | 0.71 | 1.0x10^-17^ |
| 14 | Jiba, Linga, Drodro, Rethy, Angumu, Logo, Fataki, Lita, Rimba, Nyarambe, Mahagi, Bunia | Week 19 | Week 25 | 56.34 | 46 | 3.86 | 1.0x10^-17^ |
| 15 | Kongolo, Lusangi, Mbulala, Nyunzu | Week 18 | Week 18 | 97.97 | 13 | 0.25 | 8.7x10^-15^ |
| 16 | Kaniama | Week 13 | Week 13 | 0 | 10 | 0.23 | 6.1x10^-10^ |
| 17 | Kabondo | Week 2 | Week 2 | 0 | 5 | 0.098 | 0.00035 |
