# Supplementary material for: Modalities and preferred routes of geographic spread of cholera from endemic areas in eastern Democratic Republic of the Congo
Source: PLoS One. 2022 Feb 7;17(2):e0263160. doi: 10.1371/journal.pone.0263160 (PMC8820636; doi:10.1371/journal.pone.0263160)
Supplement: S6 Table — (DOCX) [file pone.0263160.s009.docx]

**S6 Table.** Spatiotemporal clusters of cholera cases, DRC, 2005.

| **Cluster number** | **Health zones** | **Start time** | **End time** | **Radius (km)** | **Observed cases** | **Expected cases** | ***p*** |
| --- | --- | --- | --- | --- | --- | --- | --- |
| 1 | Haut Plateau, Uvira, Lemera, Ruzizi, Kaziba, Nundu, Itombwe, Mwana, Mwenga, Nyangezi, Mubumbano, Nyatende, Walungu, Bagira Kasha, Kadutu, Ibanda, Minembwe, Kamituga, Kaniola, Kabare, Kitutu, Kalonge, Miti Murhesa, Idjwi, Kimbi Lulenge, Fizi, Katana | Week 1 | Week 19 | 121.23 | 3500 | 1726.02 | 1.0x10^-17^ |
| 2 | Kailo, Kindu, Alunguli, Kalima, Lowa | Week 35 | Week 41 | 112.10 | 1337 | 387.33 | 1.0x10^-17^ |
| 3 | Manguredjipa, Musienene, Biena, Alimbongo, Mabalako, Butembo, Vohovi, Pinga, Mambasa, Kayna, Lubero, Niania, Masereka, Beni | Week 33 | Week 37 | 112.88 | 1081 | 270.11 | 1.0x10^-17^ |
| 4 | Kabalo, Ankoro, Mbulala, Kongolo | Week 48 | Week 51 | 100.92 | 264 | 15.10 | 1.0x10^-17^ |
| 5 | Kirotshe, Goma, Karisimbi, Nyiragongo, Kitoyi, Minova, Masisi, Birambizo | Week 20 | Week 33 | 32.97 | 1470 | 618.10 | 1.0x10^-17^ |
| 6 | Nyakunde | Week 6 | Week 10 | 0 | 260 | 32.04 | 1.0x10^-17^ |
| 7 | Boga, Gethy, Kamango, Komanda, Rwampara, Oicha, Tchomia, Nizi, Mutwanga | Week 14 | Week 19 | 72.14 | 334 | 65.06 | 1.0x10^-17^ |
| 8 | Angumu, Nyarambe, Rethy, Linga, Jiba, Logo | Week 36 | Week 41 | 27.10 | 395 | 102.55 | 1.0x10^-17^ |
| 9 | Moba | Week 50 | Week 50 | 0 | 93 | 3.49 | 1.0x10^-17^ |
| 10 | Nyemba, Kalemie | Week 38 | Week 46 | 87.81 | 848 | 443.54 | 1.0x10^-17^ |
| 11 | Kabondo Dianda, Butumba, Malemba Nkulu, Kamina Base, Bukama, Kinkondja | Week 1 | Week 8 | 92.11 | 283 | 93.30 | 1.0x10^-17^ |
| 12 | Gombari, Mandima | Week 17 | Week 19 | 71.58 | 22 | 0.71 | 1.0x10^-17^ |
| 13 | Lukafu, Kikula, Kasenga, Bunkeya, Kapolobwe, Kafubu, Kambove, Lubumbashi, Tshamilemba, Vangu, Kowe, Ruashi, Likasi, Kipushi, Kamalondo, Kapemba, Mufunga Sampwe, Mubunda | Week 42 | Week 43 | 116.39 | 15 | 0.84 | 2.3x10^-11^ |
| 14 | Kapanga | Week 5 | Week 7 | 0 | 23 | 3.98 | 1.3x10^-07^ |
| 15 | Kabondo | Week 44 | Week 44 | 0 | 6 | 0.077 | 4.6x10^-07^ |
| 16 | Aba, Laybo, Adi, Faradje, Makoro, Adia, Ariwara, Biringi | Week 50 | Week 50 | 100.64 | 6 | 0.11 | 4.2x10^-06^ |
| 17 | Tshilundu, Miabi, Tshitshimbi, Kamiji, Bonzola, Bimpemba, Lubilanji, Kansele, Kabeya Kamuanga, Diulu, Dibindi, Tshilenge, Muya, Lukelenge, Tshitenge, Mukumbi, Lubondaie, Mulumba, Mwene Ditu, Makota | Week 19 | Week 30 | 66.74 | 13 | 1.72 | 0.0002 |
| 18 | Bafwabogbo, Bafwasende, Opienge, Wanie Rukula | Week 11 | Week 11 | 117.02 | 4 | 0.068 | 0.0031 |
