# Supplementary material for: Modalities and preferred routes of geographic spread of cholera from endemic areas in eastern Democratic Republic of the Congo
Source: PLoS One. 2022 Feb 7;17(2):e0263160. doi: 10.1371/journal.pone.0263160 (PMC8820636; doi:10.1371/journal.pone.0263160)
Supplement: S7 Table — (DOCX) [file pone.0263160.s010.docx]

**S7 Table.** Spatiotemporal clusters of cholera cases, DRC, 2006.

| **Cluster number** | **Health zones** | **Start time** | **End time** | **Radius (km)** | **Observed cases** | **Expected cases** | ***p*** |
| --- | --- | --- | --- | --- | --- | --- | --- |
| 1 | Kadutu, Bagira Kasha, Ibanda, Nyatende, Kabare, Walungu, Nyangezi, Mubumbano, Kaziba, Miti Murhesa, Kalonge, Idjwi, Mwana, Kaniola | Week 46 | Week 52 | 46.83 | 3857 | 1340.71 | 1.0x10^-17^ |
| 2 | Kinkondja, Lwamba, Malemba Nkulu, Kabongo, Ankoro Kabondo Dianda | Week 2 | Week 7 | 92.11 | 1695 | 420.79 | 1.0x10^-17^ |
| 3 | Nyiragongo, Karisimbi, Goma, Kirotshe, Rutshuru | Week 22 | Week 29 | 28.70 | 1909 | 541.71 | 1.0x10^-17^ |
| 4 | Pweto | Week 42 | Week 45 | 0 | 457 | 42.63 | 1.0x10^-17^ |
| 5 | Tumba | Week 39 | Week 43 | 0 | 493 | 59.77 | 1.0x10^-17^ |
| 6 | Kamango, Oicha, Boga, Mutwanga, Kalunguta, Beni, Komanda, Gethy | Week 30 | Week 38 | 54.08 | 471 | 85.58 | 1.0x10^-17^ |
| 7 | Nyemba, Kalemie, Fizi | Week 32 | Week 42 | 107.35 | 1710 | 808.02 | 1.0x10^-17^ |
| 8 | Laybo, Adi | Week 35 | Week 36 | 24.34 | 139 | 6.09 | 1.0x10^-17^ |
| 9 | Pangi | Week 41 | Week 45 | 0 | 239 | 30.89 | 1.0x10^-17^ |
| 10 | Lita, Bunia, Drodro, Fataki, Jiba, Linga, Nizi, Tchomia, Mangala, Bambu | Week 1 | Week 15 | 47.08 | 1138 | 543.51 | 1.0x10^-17^ |
| 11 | Kinda, Kilela Balanda | Week 46 | Week 46 | 85.38 | 47 | 0.83 | 1.0x10^-17^ |
| 12 | Kitutu, Kamituga, Kakole, Itombwe, Mwenga | Week 18 | Week 21 | 65.59 | 73 | 4.38 | 1.0x10^-17^ |
| 13 | Moanda, Kitona, Boma Bungu | Week 19 | Week 37 | 55.44 | 209 | 61.76 | 1.0x10^-17^ |
| 14 | Walikale | Week 13 | Week 15 | 0 | 60 | 5.22 | 1.0x10^-17^ |
| 15 | Samba, Mbulala, Kasongo, Kunda, Lusangi, Kibombo, Lubao, Salamabila, Kongolo | Week 48 | Week 52 | 123.60 | 109 | 21.79 | 1.0x10^-17^ |
| 16 | Kyondo | Week 1 | Week 6 | 0 | 137 | 34.63 | 1.0x10^-17^ |
| 17 | Kimpangu, Kwilu Ngongo | Week 42 | Week 46 | 37.09 | 60 | 6.65 | 1.0x10^-17^ |
| 18 | Mufunga Sampwe, Bunkeya, Mitwaba, Butumba | Week 21 | Week 31 | 110.22 | 100 | 21.65 | 1.0x10^-17^ |
| 19 | Manguredjipa, Musienene, Biena, Alimbongo, Mabalako, Butembo, Vohovi, Katwa, Pinga | Week 45 | Week 48 | 97.58 | 115 | 34.59 | 1.0x10^-17^ |
| 20 | Kabalo | Week 43 | Week 47 | 0 | 48 | 6.19 | 1.0x10^-17^ |
| 21 | Tembo | Week 29 | Week 31 | 0 | 18 | 0.97 | 1.7x10^-13^ |
| 22 | Kipushi | Week 48 | Week 48 | 0 | 6 | 0.15 | 9.1x10^-05^ |
| 23 | Kayamba, Kalonda Est, Kitenge | Week 33 | Week 33 | 82.02 | 4 | 0.068 | 0.0052 |
