# Supplementary material for: Modalities and preferred routes of geographic spread of cholera from endemic areas in eastern Democratic Republic of the Congo
Source: PLoS One. 2022 Feb 7;17(2):e0263160. doi: 10.1371/journal.pone.0263160 (PMC8820636; doi:10.1371/journal.pone.0263160)
Supplement: S8 Table — (DOCX) [file pone.0263160.s011.docx]

**S8 Table.** Spatiotemporal clusters of cholera cases, DRC, 2007.

| **Cluster number** | **Health zones** | **Start time** | **End time** | **Radius (km)** | **Observed cases** | **Expected cases** | ***p*** |
| --- | --- | --- | --- | --- | --- | --- | --- |
| 1 | Rutshuru | Week 48 | Week 52 | 0 | 1466 | 228.04 | 1.0x10^-17^ |
| 2 | Nyatende, Bagira Kasha, Kadutu, Ibanda, Nyangezi, Walungu, Kabare, Kaziba, Mubumbano, Mwana, Kalonge, Lemera, Miti Murhesa | Week 1 | Week 11 | 46.77 | 3418 | 1342.83 | 1.0x10^-17^ |
| 3 | Butumba, Kabondo Dianda, Bukama | Week 2 | Week 16 | 66.35 | 4089 | 1834.61 | 1.0x10^-17^ |
| 4 | Pweto | Week 32 | Week 34 | 0 | 413 | 21.97 | 1.0x10^-17^ |
| 5 | Kapolobwe, Likasi, Kikula, Kambove, Panda, Kowe, Vangu, Kipushi, Ruashi, Kanzenze, Tshamilemba, Mubunda, Lukafu, Kisanga, Kamalondo, Bunkeya, Katuba, Fungurume, Lubumbashi, Kapemba, Kenya, Kafubu, Manika | Week 41 | Week 52 | 122.08 | 1587 | 518.92 | 1.0x10^-17^ |
| 6 | Minova, Kitoyi, Bunyakiri, Katana, Kirotshe, Goma | Week 41 | Week 47 | 34.89 | 2153 | 882.18 | 1.0x10^-17^ |
| 7 | Nyemba, Kalemie | Week 19 | Week 38 | 87.81 | 2244 | 975.18 | 1.0x10^-17^ |
| 8 | Lwamba, Malemba Nkulu, Kinkondja, Mukanga, Mulongo, Ankoro | Week 36 | Week 41 | 95.71 | 852 | 206.94 | 1.0x10^-17^ |
| 9 | Moba, Kasimba | Week 37 | Week 47 | 101.09 | 1498 | 583.74 | 1.0x10^-17^ |
| 10 | Kongolo, Lusangi, Mbulala, Nyunzu, Kabalo | Week 39 | Week 42 | 100.92 | 597 | 116.83 | 1.0x10^-17^ |
| 11 | Butembo, Katwa, Vohovi, Masereka, Biena, Kyondo, Lubero, Mabalako, Kalunguta, Beni, Alimbongo, Mutwanga, Musienene, Kayna | Week 16 | Week 19 | 66.78 | 211 | 15.08 | 1.0x10^-17^ |
| 12 | Walikale, Itebero, Kibua, Punia, Pinga | Week 22 | Week 37 | 91.89 | 474 | 111.29 | 1.0x10^-17^ |
| 13 | Sakania | Week 45 | Week 46 | 0 | 132 | 8.67 | 1.0x10^-17^ |
| 14 | Lita, Bunia, Drodro, Fataki, Jiba, Linga, Nizi, Tchomia | Week 33 | Week 38 | 46.30 | 241 | 44.58 | 1.0x10^-17^ |
| 15 | Kimbi Lulenge, Minembwe, Kabambare, Itombwe, Fizi, Kitutu, Nundu, Kamituga, Kampene, Kakole, Haut Plateau, Uvira | Week 22 | Week 30 | 117.12 | 824 | 410.27 | 1.0x10^-17^ |
| 16 | Ariwara, Laybo, Adi, Aru, Adia, Aungba, Biringi, Makoro, Aba, Mahagi, Kambala, Rimba, Logo, Mangala, Rethy, Nyarambe | Week 43 | Week 46 | 111.49 | 78 | 13.22 | 1.0x10^-17^ |
| 17 | Mandima, Lolwa, Gombari, Kilo, Damasi, Mongbwalu, Mambasa, Nyakunde | Week 28 | Week 28 | 93.12 | 15 | 0.19 | 1.0x10^-17^ |
| 18 | Mutshatsha, Dilala, Kilela Balanda, Lualaba, Kasaji | Week 45 | Week 50 | 121.56 | 37 | 5.10 | 1.1x10^-16^ |
| 19 | Kapanga | Week 46 | Week 47 | 0 | 18 | 2.25 | 1.6x10^-07^ |
| 20 | Kuimba | Week 5 | Week 9 | 0 | 18 | 2.57 | 1.7x10^-06^ |
