# Supplementary material for: Modalities and preferred routes of geographic spread of cholera from endemic areas in eastern Democratic Republic of the Congo
Source: PLoS One. 2022 Feb 7;17(2):e0263160. doi: 10.1371/journal.pone.0263160 (PMC8820636; doi:10.1371/journal.pone.0263160)
Supplement: S9 Table — (DOCX) [file pone.0263160.s012.docx]

**S9 Table.** Spatiotemporal clusters of cholera cases, DRC, 2008.

| **Cluster number** | **Health zones** | **Start time** | **End time** | **Radius (km)** | **Observed cases** | **Expected cases** | ***p*** |
| --- | --- | --- | --- | --- | --- | --- | --- |
| 1 | Kapolobwe, Likasi, Kikula, Kambove, Panda, Kowe, Vangu, Kipushi, Ruashi, Kanzenze, Tshamilemba, Mubunda, Lukafu, Kisanga, Kamalondo, Bunkeya, Katuba, Fungurume, Lubumbashi, Kapemba, Kenya, Kafubu, Manika | Week 2 | Week 11 | 122.08 | 5099 | 1984.60 | 1.0x10^-17^ |
| 2 | Kibua, Masisi, Kitoyi, Mweso, Itebero, Pinga, Kirotshe, Walikale, Minova, Kahele, Birambizo | Week 17 | Week 31 | 77.90 | 3043 | 1241.51 | 1.0x10^-17^ |
| 3 | Kabambare, Kimbi Lulenge, Minembwe, Kampene, Lusangi, Nyunzu, Kongolo, Fizi | Week 32 | Week 43 | 112.62 | 1126 | 364.78 | 1.0x10^-17^ |
| 4 | Kyondo, Masereka, Katwa, Butembo, Vohovi, Lubero, Mutwanga, Kalunguta, Beni, Mabalako, Biena, Alimbongo, Oicha, Kamango, Kayna, Binza, Musienene, Kibirizi, Boga, Komanda, Bambo, Rwanguba | Week 44 | Week 50 | 118.45 | 469 | 122.10 | 1.0x10^-17^ |
| 5 | Kindu, Alunguli, Kailo | Week 44 | Week 45 | 54.98 | 187 | 16.38 | 1.0x10^-17^ |
| 6 | Kabondo Dianda, Butumba, Malemba Nkulu, Kamina Base, Bukama, Kinkondja, Lwamba | Week 1 | Week 3 | 99.89 | 390 | 90.81 | 1.0x10^-17^ |
| 7 | Ruzizi, Lemera, Uvira, Haut Plateau, Nyangezi, Kaziba, Mwana, Nyatende, Nundu, Mubumbano, Bagira Kasha, Kadutu, Ibanda, Walungu, Mwenga, Itombwe, Kabare, Idjwi | Week 48 | Week 52 | 94.42 | 637 | 245.64 | 1.0x10^-17^ |
| 8 | Linga | Week 33 | Week 38 | 0 | 110 | 7.71 | 1.0x10^-17^ |
| 9 | Wanie Rukula | Week 9 | Week 13 | 0 | 130 | 13.14 | 1.0x10^-17^ |
| 10 | Ariwara, Laybo, Adi, Aru, Adia, Aungba, Biringi, Makoro, Aba, Mahagi, Kambala, Rimba, Logo, Mangala, Rethy, Nyarambe | Week 5 | Week 7 | 111.49 | 239 | 56.59 | 1.0x10^-17^ |
| 11 | Lita, Bunia, Drodro | Week 1 | Week 3 | 23.01 | 173 | 31.41 | 1.0x10^-17^ |
| 12 | Kalemie, Kasimba, Nyemba | Week 27 | Week 28 | 87.81 | 209 | 49.39 | 1.0x10^-17^ |
| 13 | Mutshatsha, Dilala, Kilela Balanda, Lualaba, Kasaji | Week 8 | Week 16 | 121.56 | 243 | 78.18 | 1.0x10^-17^ |
| 14 | Pweto | Week 31 | Week 38 | 0 | 99 | 18.30 | 1.0x10^-17^ |
| 15 | Manono, Mulongo, Kiambi, Ankoro, Kabalo | Week 35 | Week 41 | 105.29 | 112 | 26.87 | 1.0x10^-17^ |
| 16 | Mandima, Lolwa, Gombari, Kilo, Damasi, Mongbwalu, Mambasa, Nyakunde | Week 20 | Week 20 | 93.12 | 22 | 0.54 | 1.0x10^-17^ |
| 17 | Kaniama | Week 14 | Week 16 | 0 | 16 | 0.91 | 1.7x10^-11^ |
| 18 | Lubao, Kamana, Kitenge, Mbulala | Week 41 | Week 43 | 93.75 | 17 | 1.38 | 9.9x10^-10^ |
