# Supplementary material for: Modalities and preferred routes of geographic spread of cholera from endemic areas in eastern Democratic Republic of the Congo
Source: PLoS One. 2022 Feb 7;17(2):e0263160. doi: 10.1371/journal.pone.0263160 (PMC8820636; doi:10.1371/journal.pone.0263160)
Supplement: S10 Table — (DOCX) [file pone.0263160.s013.docx]

**S10 Table.** Spatiotemporal clusters of cholera cases, DRC, 2009.

| **Cluster number** | **Health zones** | **Start time** | **End time** | **Radius (km)** | **Observed cases** | **Expected cases** | ***p*** |
| --- | --- | --- | --- | --- | --- | --- | --- |
| 1 | Karisimbi, Goma | Week 33 | Week 35 | 3.56 | 916 | 194.61 | 1.0x10^-17^ |
| 2 | Lualaba, Dilala, Manika, Fungurume, Kilela Balanda, Mutshatsha, Lubudi | Week 7 | Week 20 | 113.63 | 893 | 192.14 | 1.0x10^-17^ |
| 3 | Kabare, Kadutu, Ibanda, Bagira Kasha, Nyatende, Walungu, Miti Murhesa, Kalonge, Bunyakiri | Week 39 | Week 52 | 35.54 | 2557 | 1392.54 | 1.0x10^-17^ |
| 4 | Nyemba, Kalemie | Week 32 | Week 40 | 87.81 | 1484 | 680.46 | 1.0x10^-17^ |
| 5 | Idjwi | Week 1 | Week 3 | 0 | 233 | 20.25 | 1.0x10^-17^ |
| 6 | Mitwaba, Mukanga, Kilwa, Mufunga Sampwe, Mulongo, Malemba Nkulu | Week 43 | Week 51 | 113.87 | 422 | 100.63 | 1.0x10^-17^ |
| 7 | Biena, Butembo, Katwa, Vohovi, Mabalako, Musienene, Alimbongo, Lubero, Masereka, Manguredjipa | Week 1 | Week 1 | 58.71 | 112 | 3.54 | 1.0x10^-17^ |
| 8 | Ankoro | Week 29 | Week 30 | 0 | 83 | 1.36 | 1.0x10^-17^ |
| 9 | Tchomia, Bunia, Nizi, Gethy, Rwampara, Lita, Boga, Bambu, Drodro, Fataki, Kilo, Jiba, Mangala, Komanda, Kamango, Linga, Mongbwalu, Rethy, Rimba, Nyakunde, Lolwa, Kambala, Logo, Angumu, Oicha, Mahagi, Mutwanga | Week 10 | Week 12 | 120.51 | 125 | 15.28 | 1.0x10^-17^ |
| 10 | Walikale, Itebero, Kibua, Punia, Pinga | Week 21 | Week 23 | 91.89 | 94 | 10.40 | 1.0x10^-17^ |
| 11 | Minembwe, Kimbi Lulenge, Itombwe, Nundu, Fizi | Week 36 | Week 39 | 69.75 | 746 | 416.24 | 1.0x10^-17^ |
| 12 | Moba | Week 31 | Week 32 | 0 | 43 | 2.53 | 1.0x10^-17^ |
| 13 | Kapanga | Week 31 | Week 31 | 0 | 17 | 0.26 | 1.0x10^-17^ |
| 14 | Kongolo, Lusangi, Mbulala, Nyunzu | Week 4 | Week 4 | 97.97 | 34 | 3.89 | 1.0x10^-17^ |
| 15 | Laybo, Adi, Ariwara, Adia, Aba, Aru | Week 11 | Week 14 | 52.60 | 21 | 1.12 | 1.0x10^-17^ |
| 16 | Kafubu, Lubumbashi, Kapemba, Kamalondo, Kenya, Tshamilemba, Katuba, Vangu, Kowe, Ruashi, Mubunda, Kipushi, Kisanga, Lukafu | Week 1 | Week 2 | 91.93 | 19 | 1.52 | 1.0x10^-11^ |
| 17 | Kindu, Alunguli, Kailo | Week 1 | Week 9 | 54.98 | 22 | 4.00 | 1.6x10^-06^ |
| 18 | Kamina Base, Songa, Kamina, Kabondo Dianda, Kinda, Bukama | Week 8 | Week 16 | 108.44 | 26 | 7.12 | 0.0003 |
