# Supplementary material for: Modalities and preferred routes of geographic spread of cholera from endemic areas in eastern Democratic Republic of the Congo
Source: PLoS One. 2022 Feb 7;17(2):e0263160. doi: 10.1371/journal.pone.0263160 (PMC8820636; doi:10.1371/journal.pone.0263160)
Supplement: S11 Table — (DOCX) [file pone.0263160.s014.docx]

**S11 Table.** Spatiotemporal clusters of cholera cases, DRC, 2010.

| **Cluster number** | **Health zones** | **Start time** | **End time** | **Radius (km)** | **Observed cases** | **Expected cases** | ***p*** |
| --- | --- | --- | --- | --- | --- | --- | --- |
| 1 | Shabunda | Week 33 | Week 37 | 0 | 783 | 110.07 | 1.0x10^-17^ |
| 2 | Masereka, Lubero, Kyondo, Katwa, Butembo, Vohovi, Alimbongo, Biena, Kalunguta, Mabalako, Kayna, Mutwanga, Beni, Musienene, Binza, Kibirizi, Oicha, Kamango, Bambo, Manguredjipa, Pinga, Rwanguba | Week 13 | Week 23 | 112.20 | 914 | 265.41 | 1.0x10^-17^ |
| 3 | Kaziba, Mwana, Nyangezi, Mubumbano, Nyatende, Walungu, Lemera, Bagira Kasha, Kadutu, Ibanda, Mwenga, Haut Plateau, Ruzizi, Kabare | Week 1 | Week 6 | 46.58 | 893 | 284.17 | 1.0x10^-17^ |
| 4 | Butumba, Kabondo Dianda, Bukama, Malemba Nkulu, Lwamba, Mufunga Sampwe, Mukanga, Kinkondja | Week 22 | Week 30 | 118.02 | 958 | 330.67 | 1.0x10^-17^ |
| 5 | Kabalo, Ankoro, Mbulala, Kongolo | Week 44 | Week 46 | 100.92 | 269 | 34.32 | 1.0x10^-17^ |
| 6 | Kitoyi, Masisi, Minova, Kirotshe | Week 42 | Week 51 | 28.11 | 982 | 395.87 | 1.0x10^-17^ |
| 7 | Nundu | Week 31 | Week 33 | 0 | 261 | 38.15 | 1.0x10^-17^ |
| 8 | Kalemie, Kasimba, Nyemba, Nyunzu | Week 38 | Week 42 | 115.22 | 458 | 153.90 | 1.0x10^-17^ |
| 9 | Kiambi, Manono | Week 43 | Week 43 | 86.61 | 22 | 0.53 | 1.0x10^-17^ |
| 10 | Kilwa | Week 10 | Week 18 | 0 | 66 | 11.95 | 1.0x10^-17^ |
| 11 | Kapanga | Week 39 | Week 41 | 0 | 47 | 10.57 | 6.7x10^-14^ |
| 12 | Kapolobwe | Week 32 | Week 33 | 0 | 13 | 0.37 | 6.7x10^-14^ |
| 13 | Lolwa, Komanda, Mandima, Kilo, Rwampara, Boga, Gethy | Week 12 | Week 15 | 75.42 | 16 | 1.61 | 2.8x10^-08^ |
| 14 | Mbanza Ngungu | Week 43 | Week 43 | 0 | 6 | 0.094 | 1.2x10^-06^ |
| 15 | Songa, Kamina Base, Kafakumba, Kabongo | Week 45 | Week 45 | 102.23 | 5 | 0.13 | 0.0008 |
| 16 | Jiba, Linga, Drodro | Week 46 | Week 47 | 13.81 | 6 | 0.39 | 0.014 |
