# Supplementary material for: Modalities and preferred routes of geographic spread of cholera from endemic areas in eastern Democratic Republic of the Congo
Source: PLoS One. 2022 Feb 7;17(2):e0263160. doi: 10.1371/journal.pone.0263160 (PMC8820636; doi:10.1371/journal.pone.0263160)
Supplement: S12 Table — (DOCX) [file pone.0263160.s015.docx]

**S12 Table.** Spatiotemporal clusters of cholera cases, DRC, 2011.

| **Cluster number** | **Health zones** | **Start time** | **End time** | **Radius (km)** | **Observed cases** | **Expected cases** | ***p*** |
| --- | --- | --- | --- | --- | --- | --- | --- |
| 1 | Bolobo | Week 23 | Week 26 | 0 | 960 | 136.18 | 1.0x10^-17^ |
| 2 | Isangi, Yakusu, Yabahondo, Bengamisa, Basoko, Mangobo, Basali, Tshopo, Lubunga | Week 9 | Week 17 | 94.80 | 799 | 125.89 | 1.0x10^-17^ |
| 3 | Idjwi, Katana, Bunyakiri, Minova, Kabare, Ibanda, Miti Murhesa, Kadutu, Bagira Kasha | Week 39 | Week 52 | 45.64 | 2110 | 849.28 | 1.0x10^-17^ |
| 4 | Makoro, Adia, Biringi, Watsa, Laybo, Aba, Faradje, Ariwara, Aungba, Damasi, Adi, Aru, Nyakunde, Kambala, Mongbwalu, Mangala, Rimba, Mahagi, Kilo, Gombari | Week 11 | Week 17 | 123.09 | 438 | 60.28 | 1.0x10^-17^ |
| 5 | Irebu, Ntondo, Bikoro, Lukolela, Mbandaka, Iboko, Bandjau, Lilanga Bobangi, Bolenge | Week 26 | Week 36 | 116.97 | 864 | 283.54 | 1.0x10^-17^ |
| 6 | Kalunguta, Beni, Mutwanga, Oicha, Mabalako, Vohovi | Week 43 | Week 46 | 34.15 | 216 | 21.49 | 1.0x10^-17^ |
| 7 | Lulingu, Shabunda, Punia, Mulungu, Itebero, Kalima, Ferekeni, Kaniola, Kamituga, Kahele, Kalonge | Week 14 | Week 20 | 120.99 | 241 | 28.81 | 1.0x10^-17^ |
| 8 | Birambizo | Week 1 | Week 4 | 0 | 173 | 12.14 | 1.0x10^-17^ |
| 9 | Tchomia | Week 50 | Week 52 | 0 | 210 | 21.21 | 1.0x10^-17^ |
| 10 | Kabalo, Ankoro, Mbulala | Week 38 | Week 40 | 89.14 | 181 | 15.30 | 1.0x10^-17^ |
| 11 | Lubutu, Obokote, Opienge, Walikale | Week 32 | Week 37 | 103.69 | 299 | 54.95 | 1.0x10^-17^ |
| 12 | Mokala, Djuma, Sia, Kimputu, Bulungu, Bosobe, Ipamu | Week 31 | Week 38 | 88.20 | 345 | 81.98 | 1.0x10^-17^ |
| 13 | Fizi, Nundu, Minembwe, Kimbi Lulenge, Uvira | Week 1 | Week 20 | 98.63 | 1418 | 765.44 | 1.0x10^-17^ |
| 14 | Lubero | Week 19 | Week 23 | 0 | 150 | 13.90 | 1.0x10^-17^ |
| 15 | Mankanza, Bomongo, Mampoko, Bokonzi, Bosomondanda | Week 18 | Week 26 | 101.75 | 401 | 131.52 | 1.0x10^-17^ |
| 16 | Lwamba, Malemba Nkulu | Week 1 | Week 10 | 35.12 | 135 | 17.06 | 1.0x10^-17^ |
| 17 | Kingabwa, Barumbu, Limeté, Masina II, Kalamu II | Week 46 | Week 51 | 4.56 | 243 | 61.03 | 1.0x10^-17^ |
| 18 | Kinkondja | Week 51 | Week 52 | 0 | 103 | 9.74 | 1.0x10^-17^ |
| 19 | Kasimba, Pweto, Kalemie, Moba | Week 1 | Week 8 | 101.09 | 354 | 129.99 | 1.0x10^-17^ |
| 20 | Opala, Ikela, Yaleko, Yahisule | Week 17 | Week 21 | 110.45 | 68 | 7.02 | 1.0x10^-17^ |
| 21 | Bandundu, Bagata, Nioki, Mushie | Week 29 | Week 34 | 84.95 | 112 | 26.44 | 1.0x10^-17^ |
| 22 | Kapanga | Week 47 | Week 47 | 0 | 34 | 1.45 | 1.0x10^-17^ |
| 23 | Mbaya, Ndage, Bulu, Binga, Karawa, Bominenge | Week 26 | Week 31 | 69.14 | 81 | 16.07 | 1.0x10^-17^ |
| 24 | Lusangi, Kampene, Kongolo | Week 41 | Week 45 | 63.79 | 61 | 9.85 | 1.0x10^-17^ |
| 25 | Bolomba, Pendjwa, Monika, Ingende | Week 28 | Week 30 | 93.17 | 31 | 2.95 | 1.0x10^-17^ |
| 26 | Yamaluka, Yambuku, Bosondjo, Bumba, Yamongili, Abuzi, Lisala, Lolo, Bosomanzi | Week 32 | Week 36 | 113.42 | 62 | 13.86 | 1.0x10^-17^ |
| 27 | Boto, Mawuya, Kungu, Bwamanda, Tandala, Bangabola | Week 44 | Week 46 | 88.31 | 23 | 2.12 | 2.0x10^-12^ |
| 28 | Boko, Kenge, Maluku I | Week 27 | Week 30 | 79.82 | 55 | 17.79 | 2.3x10^-08^ |
| 29 | Monkoto | Week 31 | Week 32 | 0 | 7 | 0.38 | 0.0022 |
| 30 | Wema | Week 36 | Week 36 | 0 | 5 | 0.13 | 0.0033 |
| 31 | Ngidinga, Nselo, Kimvula, Boko Kivulu, Kisantu, Kimpangu, Sona Bata, Kwilu Ngongo, Mbanza Ngungu, Gombe Matadi, Massa, Mont Ngafula I | Week 30 | Week 32 | 122.70 | 9 | 0.85 | 0.0044 |
| 32 | Zongo | Week 13 | Week 23 | 0 | 36 | 14.13 | 0.022 |
