# Supplementary material for: Modalities and preferred routes of geographic spread of cholera from endemic areas in eastern Democratic Republic of the Congo
Source: PLoS One. 2022 Feb 7;17(2):e0263160. doi: 10.1371/journal.pone.0263160 (PMC8820636; doi:10.1371/journal.pone.0263160)
Supplement: S13 Table — (DOCX) [file pone.0263160.s016.docx]

**S13 Table.** Spatiotemporal clusters of cholera cases, DRC, 2012.

| **Cluster number** | **Health zones** | **Start time** | **End time** | **Radius (km)** | **Observed cases** | **Expected cases** | ***p*** |
| --- | --- | --- | --- | --- | --- | --- | --- |
| 1 | Karisimbi | Week 31 | Week 36 | 0 | 1542 | 295.90 | 1.0x10^-17^ |
| 2 | Rwanguba | Week 25 | Week 28 | 0 | 891 | 86.34 | 1.0x10^-17^ |
| 3 | Mubumbano, Mwana, Walungu, Kaziba, Nyatende, Bagira Kasha, Nyangezi, Kaniola, Kadutu, Mwenga, Ibanda, Kabare, Kalonge, Lemera, Miti Murhesa, Haut Plateau, Ruzizi | Week 1 | Week 5 | 64.71 | 1084 | 191.48 | 1.0x10^-17^ |
| 4 | Mulongo, Mukanga, Manono, Lwamba, Malemba Nkulu | Week 37 | Week 44 | 96.42 | 830 | 122.42 | 1.0x10^-17^ |
| 5 | Bolobo, Yumbi | Week 8 | Week 12 | 60.96 | 736 | 151.79 | 1.0x10^-17^ |
| 6 | Kambala, Mangala, Aungba, Rimba, Fataki, Mahagi, Biringi, Logo, Nyakunde, Rethy, Aru, Linga, Mongbwalu, Bambu, Jiba, Lita, Drodro, Damasi, Adia, Angumu, Bunia, Kilo, Nyarambe, Nizi, Ariwara, Makoro, Rwampara, Laybo | Week 11 | Week 22 | 95.99 | 1882 | 788.50 | 1.0x10^-17^ |
| 7 | Abuzi, Wasolo, Yambuku, Yakoma, Businga, Yamaluka, Wapinda, Yamongili | Week 15 | Week 23 | 104.61 | 602 | 153.62 | 1.0x10^-17^ |
| 8 | Moba, Kasimba | Week 43 | Week 48 | 101.09 | 266 | 30.95 | 1.0x10^-17^ |
| 9 | Bwamanda, Tandala, Gemena, Boto, Bongosenubia, Mawuya, Bominenge, Kungu, Libenge, Budjala, Bulu, Karawa, Bangabola, Bosobolo, Zongo | Week 21 | Week 27 | 124.16 | 466 | 104.93 | 1.0x10^-17^ |
| 10 | Yalifafo | Week 17 | Week 19 | 0 | 189 | 15.82 | 1.0x10^-17^ |
| 11 | Tchomia | Week 1 | Week 9 | 0 | 856 | 350.74 | 1.0x10^-17^ |
| 12 | Mutwanga, Kalunguta, Beni, Kyondo, Kamango, Oicha, Vohovi, Katwa, Masereka, Butembo, Mabalako, Boga, Lubero, Komanda, Biena, Gethy, Alimbongo, Binza | Week 38 | Week 42 | 109.79 | 306 | 63.73 | 1.0x10^-17^ |
| 13 | Bengamisa, Tshopo, Mangobo, Kabondo, Yakusu, Makiso Kisangani, Lubunga, Isangi, Banalia | Week 13 | Week 16 | 87.72 | 240 | 42.50 | 1.0x10^-17^ |
| 14 | Kamina Base, Songa, Kamina, Kabondo Dianda, Kinda, Bukama | Week 17 | Week 21 | 108.44 | 378 | 109.48 | 1.0x10^-17^ |
| 15 | Lowa | Week 15 | Week 19 | 0 | 212 | 41.09 | 1.0x10^-17^ |
| 16 | Walikale, Itebero, Kibua, Punia, Pinga, Lubutu, Masisi | Week 43 | Week 45 | 111.76 | 154 | 21.22 | 1.0x10^-17^ |
| 17 | Kabalo, Ankoro, Mbulala, Kongolo | Week 44 | Week 51 | 100.92 | 114 | 12.14 | 1.0x10^-17^ |
| 18 | Shabunda | Week 19 | Week 25 | 0 | 156 | 25.52 | 1.0x10^-17^ |
| 19 | Boende, Wema, Monika | Week 16 | Week 20 | 90.68 | 150 | 24.08 | 1.0x10^-17^ |
| 20 | Yahuma | Week 24 | Week 28 | 0 | 113 | 12.64 | 1.0x10^-17^ |
| 21 | Bikoro, Mbandaka | Week 13 | Week 17 | 46.04 | 146 | 24.75 | 1.0x10^-17^ |
| 22 | Gombari | Week 14 | Week 18 | 0 | 134 | 20.98 | 1.0x10^-17^ |
| 23 | Bili | Week 14 | Week 18 | 0 | 126 | 19.86 | 1.0x10^-17^ |
| 24 | Djombo, Bosomondanda, Basankusu, Binga, Pimu, Befale, Bongandanga | Week 14 | Week 28 | 121.8 | 437 | 184.36 | 1.0x10^-17^ |
| 25 | Ruashi, Kowe, Kipushi, Vangu, Mubunda, Tshamilemba, Kisanga, Kamalondo, Katuba, Kenya, Kapemba, Lubumbashi, Kapolobwe, Kafubu, Kanzenze, Panda, Kikula, Likasi | Week 41 | Week 48 | 99.43 | 88 | 8.90 | 1.0x10^-17^ |
| 26 | Nioki, Bandundu, Bokoro, Mushie | Week 28 | Week 34 | 83.12 | 124 | 20.65 | 1.0x10^-17^ |
| 27 | Fizi, Nundu, Minembwe, Kimbi Lulenge, Uvira, Itombwe, Nyemba | Week 45 | Week 51 | 107.35 | 424 | 183.30 | 1.0x10^-17^ |
| 28 | Matadi, Nzanza, Inga, Boma, Sona Pangu | Week 22 | Week 32 | 53.20 | 158 | 35.83 | 1.0x10^-17^ |
| 29 | Bondo | Week 6 | Week 9 | 0 | 132 | 26.04 | 1.0x10^-17^ |
| 30 | Mbanza Ngungu, Gombe Matadi, Boko Kivulu, Kwilu Ngongo, Kisantu, Kimpese, Kimpangu, Sona Bata, Massa, Mont Ngafula I, Mangembo, Mont Ngafula II, Nselo, Ngidinga, Selembao, Binza Météo, Lemba, Kisenso, Binza Ozone, Bumbu, Makala, Kimbanseke, Ngaba, Kintambo, Biyela | Week 21 | Week 24 | 104.07 | 94 | 14.19 | 1.0x10^-17^ |
| 31 | Iboko, Pendjwa, Ingende | Week 27 | Week 30 | 63.53 | 53 | 3.49 | 1.0x10^-17^ |
| 32 | Mufunga Sampwe, Bunkeya, Mitwaba, Butumba | Week 27 | Week 32 | 110.22 | 152 | 40.60 | 1.0x10^-17^ |
| 33 | Kalonda Est, Lukashi lualu, Kayamba, Kitenge, Kamana, Kalambayi Kabanga, Kabinda, Lubao, Tshofa, Kabongo | Week 36 | Week 42 | 123.89 | 79 | 10.80 | 1.0x10^-17^ |
| 34 | Poko, Viandana, Ganga, Isiro | Week 49 | Week 52 | 89.63 | 99 | 18.09 | 1.0x10^-17^ |
| 35 | Bokonzi | Week 18 | Week 20 | 0 | 76 | 10.18 | 1.0x10^-17^ |
| 36 | Kilwa | Week 10 | Week 12 | 0 | 40 | 2.66 | 1.0x10^-17^ |
| 37 | Kuimba, Kangu, Tshela, Kizu, Vaku, Lukula, Kinkonzi, Seke Banza, Boma Bungu, Moanda | Week 4 | Week 12 | 90.15 | 388 | 200.01 | 1.0x10^-17^ |
| 38 | Kikongo, Kenge, Bagata, Yanga bosa, Kwamouth | Week 1 | Week 3 | 90.05 | 61 | 8.41 | 1.0x10^-17^ |
| 39 | Faradje | Week 31 | Week 33 | 0 | 43 | 5.84 | 1.0x10^-17^ |
| 40 | Gbadolite, Mobayi Mbongo | Week 12 | Week 14 | 31.73 | 35 | 3.61 | 1.0x10^-17^ |
| 41 | Kapanga | Week 3 | Week 4 | 0 | 36 | 4.11 | 1.0x10^-17^ |
| 42 | Opala, Ikela, Yaleko, Yahisule | Week 20 | Week 28 | 110.45 | 51 | 9.22 | 1.0x10^-17^ |
| 43 | Oshwe, Ipamu, Ilebo, Kimputu, Mimia, Koshibanda, Bosobe | Week 38 | Week 38 | 115.35 | 13 | 0.22 | 1.8x10^-15^ |
| 44 | Monkoto | Week 16 | Week 18 | 0 | 32 | 3.89 | 4.7x10^-15^ |
| 45 | Doruma | Week 9 | Week 9 | 0 | 10 | 0.41 | 3.0x10^-07^ |
