# Supplementary material for: Modalities and preferred routes of geographic spread of cholera from endemic areas in eastern Democratic Republic of the Congo
Source: PLoS One. 2022 Feb 7;17(2):e0263160. doi: 10.1371/journal.pone.0263160 (PMC8820636; doi:10.1371/journal.pone.0263160)
Supplement: S14 Table — (DOCX) [file pone.0263160.s017.docx]

**S14 Table.** Spatiotemporal clusters of cholera cases, DRC, 2013.

| **Cluster number** | **Health zones** | **Start time** | **End time** | **Radius (km)** | **Observed cases** | **Expected cases** | ***p*** |
| --- | --- | --- | --- | --- | --- | --- | --- |
| 1 | Kafubu, Lubumbashi, Kapemba, Kamalondo, Kenya, Tshamilemba, Katuba, Vangu, Kowe, Ruashi, Mubunda, Kipushi, Kisanga, Lukafu, Kikula | Week 4 | Week 17 | 103.68 | 6349 | 3529.08 | 1.0x10^-17^ |
| 2 | Walungu, Bagira Kasha Nyatende, Kadutu, Mubumbano, Ibanda, Kabare, Nyangezi, Kaziba, Mwana, Kaniola, Kalonge, Miti Murhesa, Mwenga, Lemera, Bunyakiri, Idjwi, Kahele, Katana, Ruzizi, Haut Plateau, Minova, Kamituga, Mulungu, Uvira, Kitoyi, Itombwe, Itebero, Goma, Kirotshe | Week 24 | Week 44 | 107.52 | 3151 | 1503.24 | 1.0x10^-17^ |
| 3 | Manika, Lualaba, Kanzenze, Panda, Fungurume, Likasi | Week 48 | Week 52 | 89.37 | 457 | 59.55 | 1.0x10^-17^ |
| 4 | Nyemba, Kalemie, Fizi | Week 37 | Week 52 | 107.35 | 1528 | 792.35 | 1.0x10^-17^ |
| 5 | Lwamba, Malemba Nkulu, Kinkondja, Mukanga, Mulongo, Ankoro, Kabondo Dianda, Butumba, Manono | Week 1 | Week 9 | 111.32 | 952 | 408.32 | 1.0x10^-17^ |
| 6 | Nyarambe, Angumu, Logo, Rethy, Linga | Week 29 | Week 39 | 38.36 | 302 | 66.63 | 1.0x10^-17^ |
| 7 | Bolobo, Yumbi, Mushie, Kwamouth | Week 18 | Week 24 | 102.86 | 188 | 24.32 | 1.0x10^-17^ |
| 8 | Moba, Kilwa, Kasimba | Week 43 | Week 48 | 121.44 | 438 | 145.23 | 1.0x10^-17^ |
| 9 | Alimbongo, Kayna, Lubero, Musienene, Biena, Masereka, Butembo, Katwa, Vohovi, Kyondo, Pinga, Kibirizi, Mabalako, Manguredjipa, Binza, Kalunguta, Beni, Mweso | Week 42 | Week 48 | 93.77 | 206 | 41.06 | 1.0x10^-17^ |
| 10 | Kampene, Lusangi, Kakole, Kasongo, Kabambare, Kitutu | Week 43 | Week 46 | 97.72 | 95 | 8.84 | 1.0x10^-17^ |
| 11 | Kinda, Kilela Balanda, Kamina, Kamina Base, Bukama | Week 7 | Week 13 | 115.79 | 203 | 65.35 | 1.0x10^-17^ |
| 12 | Kilo, Mongbwalu | Week 17 | Week 21 | 20.80 | 56 | 5.86 | 1.0x10^-17^ |
| 13 | Boga, Gethy, Kamango, Komanda, Rwampara, Oicha, Tchomia, Nizi, Mutwanga | Week 9 | Week 11 | 72.14 | 184 | 65.93 | 1.0x10^-17^ |
| 14 | Lemba, Ngaba, Makala, Kisenso, Matete, Bumbu, Kalamu I, Selembao, Limeté, Ngiri Ngiri, Ndjili, Kalamu II, Kimbanseke, Kasa Vubu, Bandalungwa, Kokolo, Binza Météo, Kintambo, Lingwala, Kinshasa, Barumbu, Kingabwa, Kingasani | Week 1 | Week 3 | 9.56 | 81 | 14.96 | 1.0x10^-17^ |
| 15 | Lilanga Bobangi, Bolenge | Week 12 | Week 15 | 36.33 | 66 | 11.23 | 1.0x10^-17^ |
| 16 | Bosomondanda, Djombo, Bangabola, Basankusu, Binga, Budjala, Mankanza | Week 8 | Week 12 | 101.75 | 112 | 34.53 | 1.0x10^-17^ |
| 17 | Adi, Laybo, Ariwara, Adia, Aba, Aru, Makoro, Biringi, Aungba, Faradje, Mahagi, Kambala, Rimba | Week 14 | Week 15 | 120.98 | 49 | 8.31 | 1.0x10^-17^ |
| 18 | Moanda, Kitona, Boma Bungu, Lukula, Boma | Week 23 | Week 24 | 62.80 | 15 | 0.34 | 1.1x10^-16^ |
| 19 | Mbulala, Samba, Kongolo, Kabalo | Week 52 | Week 52 | 89.14 | 15 | 0.88 | 1.9x10^-10^ |
| 20 | Isangi | Week 33 | Week 38 | 0 | 15 | 1.18 | 1.5x10^-08^ |
| 21 | Lubao, Kamana, Kitenge | Week 1 | Week 4 | 90.33 | 26 | 4.66 | 4.4x10^-08^ |
| 22 | Niania | Week 14 | Week 16 | 0 | 17 | 2.05 | 5.5x10^-07^ |
| 23 | Bosondjo, Yamaluka, Bumba, Lingomo, Lisala, Bongandanga, Yambuku, Yamongili, Pimu | Week 23 | Week 24 | 106.45 | 7 | 0.43 | 0.0036 |
