# Supplementary material for: Modalities and preferred routes of geographic spread of cholera from endemic areas in eastern Democratic Republic of the Congo
Source: PLoS One. 2022 Feb 7;17(2):e0263160. doi: 10.1371/journal.pone.0263160 (PMC8820636; doi:10.1371/journal.pone.0263160)
Supplement: S15 Table — (DOCX) [file pone.0263160.s018.docx]

**S15 Table.** Spatiotemporal clusters of cholera cases, DRC, 2014.

| **Cluster number** | **Health zones** | **Start time** | **End time** | **Radius (km)** | **Observed cases** | **Expected cases** | ***p*** |
| --- | --- | --- | --- | --- | --- | --- | --- |
| 1 | Butumba, Kabondo Dianda, Bukama | Week 1 | Week 11 | 66.35 | 867 | 202.39 | 1.0x10^-17^ |
| 2 | Karisimbi | Week 23 | Week 29 | 0 | 273 | 24.38 | 1.0x10^-17^ |
| 3 | Kayna, Alimbongo | Week 3 | Week 6 | 23.74 | 343 | 46.88 | 1.0x10^-17^ |
| 4 | Masereka, Lubero, Kyondo | Week 33 | Week 38 | 19.61 | 377 | 58.75 | 1.0x10^-17^ |
| 5 | Mufunga Sampwe | Week 19 | Week 24 | 0 | 268 | 28.11 | 1.0x10^-17^ |
| 6 | Nyemba | Week 33 | Week 36 | 0 | 301 | 47.10 | 1.0x10^-17^ |
| 7 | Kenya, Katuba, Kamalondo, Kapemba, Kisanga, Mubunda, Vangu, Kowe, Kipushi, Kafubu, Kapolobwe, Kikula, Lukafu | Week 5 | Week 18 | 123.15 | 742 | 270.19 | 1.0x10^-17^ |
| 8 | Kabambare, Kimbi Lulenge, Minembwe, Kampene, Lusangi, Nyunzu, Kongolo | Week 36 | Week 37 | 108.41 | 158 | 14.56 | 1.0x10^-17^ |
| 9 | Kibua, Masisi, Kitoyi, Mweso, Itebero, Pinga, Kirotshe, Walikale, Minova, Kahele, Birambizo, Bunyakiri, Miti Murhesa, Goma | Week 43 | Week 50 | 93.32 | 1018 | 497.27 | 1.0x10^-17^ |
| 10 | Malemba Nkulu | Week 38 | Week 42 | 0 | 294 | 67.96 | 1.0x10^-17^ |
| 11 | Uvira | Week 12 | Week 16 | 0 | 520 | 188.18 | 1.0x10^-17^ |
| 12 | Lulingu | Week 38 | Week 39 | 0 | 117 | 8.67 | 1.0x10^-17^ |
| 13 | Pweto, Kasimba, Kiambi, Kilwa | Week 7 | Week 11 | 120.85 | 210 | 36.74 | 1.0x10^-17^ |
| 14 | Nyangezi, Nyatende, Kaziba, Bagira Kasha, Kadutu, Ibanda, Walungu, Mubumbano, Lemera, Mwana, Kabare, Ruzizi | Week 1 | Week 7 | 44.78 | 645 | 271.59 | 1.0x10^-17^ |
| 15 | Kinkondja | Week 11 | Week 17 | 0 | 625 | 269.74 | 1.0x10^-17^ |
| 16 | Ankoro | Week 45 | Week 48 | 0 | 108 | 10.42 | 1.0x10^-17^ |
| 17 | Manika, Lualaba, Kanzenze, Panda, Fungurume, Likasi | Week 1 | Week 5 | 89.37 | 206 | 45.58 | 1.0x10^-17^ |
| 18 | Mukanga | Week 22 | Week 31 | 0 | 169 | 32.26 | 1.0x10^-17^ |
| 19 | Salamabila, Pangi, Kunda, Kasongo, Alunguli, Kalima, Kindu, Kibombo, Shabunda | Week 28 | Week 30 | 108.13 | 41 | 1.32 | 1.0x10^-17^ |
| 20 | Moba | Week 39 | Week 42 | 0 | 95 | 26.90 | 1.0x10^-17^ |
| 21 | Mangala, Kambala, Fataki, Bambu, Nyakunde, Rimba, Mongbwalu, Lita, Aungba, Bunia, Kilo, Logo, Drodro, Rethy, Linga, Mahagi, Nizi | Week 1 | Week 4 | 57.50 | 16 | 1.92 | 9.1x10^-07^ |
| 22 | Lubao, Kamana, Kitenge, Mbulala, Tshofa, Kalonda Est, Samba | Week 40 | Week 41 | 110.53 | 9 | 0.59 | 5.3x10^-05^ |
| 23 | Kalambayi Kabanga, Ngandajika, Lukashi lualu, Mulumba, Kanda Kanda, Kayamba | Week 39 | Week 39 | 83.13 | 6 | 0.17 | 9.7x10^-05^ |
| 24 | Maluku II, Nsele, Kikimi, Masina I, Biyela, Maluku I, Kingasani, Masina II, Kimbanseke, Ndjili, Kingabwa, Matete, Kisenso, Limeté, Ngaba, Lemba, Barumbu, Kalamu II, Kalamu I, Makala, Kinshasa, Kasa Vubu | Week 48 | Week 48 | 53.89 | 5 | 0.10 | 0.0003 |
| 25 | Mutshatsha, Dilala, Kilela Balanda | Week 6 | Week 6 | 104.73 | 4 | 0.080 | 0.0072 |
