# Supplementary material for: Modalities and preferred routes of geographic spread of cholera from endemic areas in eastern Democratic Republic of the Congo
Source: PLoS One. 2022 Feb 7;17(2):e0263160. doi: 10.1371/journal.pone.0263160 (PMC8820636; doi:10.1371/journal.pone.0263160)
Supplement: S16 Table — (DOCX) [file pone.0263160.s019.docx]

**S16 Table.** Spatiotemporal clusters of cholera cases, DRC, 2015.

| **Cluster number** | **Health zones** | **Start time** | **End time** | **Radius (km)** | **Observed cases** | **Expected cases** | ***p*** |
| --- | --- | --- | --- | --- | --- | --- | --- |
| 1 | Kailo, Kindu, Alunguli, Kalima, Lowa | Week 36 | Week 40 | 112.10 | 2641 | 819.77 | 1.0x10^-17^ |
| 2 | Butumba, Kabondo Dianda, Bukama, Malemba Nkulu, Lwamba, Mufunga Sampwe, Mukanga, Kinkondja, Mitwaba | Week 1 | Week 8 | 119.88 | 1180 | 273.11 | 1.0x10^-17^ |
| 3 | Ruashi, Kowe, Kipushi, Vangu, Mubunda, Tshamilemba, Kisanga, Kamalondo, Katuba, Kenya, Kapemba, Lubumbashi, Kapolobwe, Kafubu, Kanzenze, Panda | Week 49 | Week 50 | 93.55 | 497 | 39.61 | 1.0x10^-17^ |
| 4 | Binza, Kibirizi, Rwanguba, Bambo, Rutshuru, Birambizo, Lubero, Kayna, Masereka, Nyiragongo, Mweso, Kyondo, Karisimbi, Alimbongo, Goma, Kirotshe, Katwa, Butembo, Vohovi, Masisi, Mutwanga, Kitoyi, Biena, Minova, Pinga | Week 12 | Week 34 | 120.33 | 1800 | 707.06 | 1.0x10^-17^ |
| 5 | Nyarambe, Angumu, Logo, Rethy, Linga, Mahagi, Jiba, Rimba | Week 51 | Week 52 | 51.07 | 183 | 10.75 | 1.0x10^-17^ |
| 6 | Samba, Mbulala, Kasongo, Kunda, Lusangi, Kibombo | Week 40 | Week 43 | 102.73 | 205 | 33.77 | 1.0x10^-17^ |
| 7 | Itombwe, Minembwe, Haut Plateau, Nundu, Mwenga, Kamituga, Uvira, Kitutu, Mwana, Lemera, Kaziba, Kimbi Lulenge, Ruzizi, Mubumbano, Nyangezi, Fizi, Bagira Kasha, Kadutu, Ibanda, Kakole, Kabare, Kalonge | Week 5 | Week 11 | 120.02 | 893 | 431.14 | 1.0x10^-17^ |
| 8 | Kalemie, Kasimba, Nyemba | Week 47 | Week 52 | 87.81 | 469 | 178.64 | 1.0x10^-17^ |
| 9 | Lolwa, Komanda, Mandima, Kilo, Rwampara, Boga, Gethy | Week 33 | Week 34 | 75.42 | 112 | 12.99 | 1.0x10^-17^ |
| 10 | Wanie Rukula | Week 39 | Week 41 | 0 | 144 | 25.18 | 1.0x10^-17^ |
| 11 | Lubutu | Week 25 | Week 36 | 0 | 114 | 16.92 | 1.0x10^-17^ |
| 12 | Moba | Week 50 | Week 52 | 0 | 109 | 15.35 | 1.0x10^-17^ |
| 13 | Lulingu | Week 41 | Week 47 | 0 | 134 | 30.02 | 1.0x10^-17^ |
| 14 | Mushie | Week 44 | Week 48 | 0 | 80 | 11.21 | 1.0x10^-17^ |
| 15 | Bengamisa, Tshopo, Mangobo, Kabondo, Yakusu, Makiso Kisangani, Lubunga | Week 43 | Week 46 | 76.91 | 60 | 8.98 | 1.0x10^-17^ |
| 16 | Manono, Mulongo, Kiambi, Ankoro, Kabalo | Week 1 | Week 8 | 105.29 | 60 | 12.80 | 1.0x10^-17^ |
| 17 | Yahuma | Week 30 | Week 30 | 0 | 5 | 0.043 | 8.7x10^-06^ |
| 18 | Kasenga, Lukafu, Kikula | Week 1 | Week 4 | 109.77 | 22 | 4.35 | 2.1x10^-05^ |
| 19 | Dilala, Mutshatsha, Kilela Balanda, Lualaba, Kinda, Lubudi | Week 47 | Week 52 | 119.73 | 20 | 5.42 | 0.013 |
