# Supplementary material for: Modalities and preferred routes of geographic spread of cholera from endemic areas in eastern Democratic Republic of the Congo
Source: PLoS One. 2022 Feb 7;17(2):e0263160. doi: 10.1371/journal.pone.0263160 (PMC8820636; doi:10.1371/journal.pone.0263160)
Supplement: S17 Table — (DOCX) [file pone.0263160.s020.docx]

**S17 Table.** Spatiotemporal clusters of cholera cases, DRC, 2016.

| **Cluster number** | **Health zones** | **Start time** | **End time** | **Radius (km)** | **Observed cases** | **Expected cases** | ***p*** |
| --- | --- | --- | --- | --- | --- | --- | --- |
| 1 | Yahuma, Djolu, Basoko | Week 8 | Week 14 | 77.72 | 941 | 187.61 | 1.0x10^-17^ |
| 2 | Mbulala, Samba, Kongolo, Kabalo | Week 43 | Week 49 | 89.14 | 453 | 49.44 | 1.0x10^-17^ |
| 3 | Mampoko, Lotumbe, Mankanza, Wangata, Bomongo, Bolenge, Lilanga Bobangi, Mbandaka | Week 19 | Week 34 | 105.13 | 1952 | 820.96 | 1.0x10^-17^ |
| 4 | Kabondo Dianda, Butumba, Malemba Nkulu, Kamina Base, Bukama, Kinkondja | Week 2 | Week 16 | 92.11 | 1457 | 524.78 | 1.0x10^-17^ |
| 5 | Kakole, Kampene, Kitutu, Kamituga, Shabunda, Kasongo, Mulungu, Lusangi, Kimbi Lulenge, Pangi, Itombwe, Kabambare, Minembwe, Mwenga, Kitutu | Week 34 | Week 46 | 124.02 | 1056 | 368.78 | 1.0x10^-17^ |
| 6 | Pinga, Kayna, Kibua, Alimbongo, Musienene, Mweso, Kibirizi, Masisi, Walikale, Lubero, Birambizo, Manguredjipa, Biena, Bambo, Kitoyi, Masereka, Kirotshe | Week 17 | Week 23 | 110.27 | 690 | 186.02 | 1.0x10^-17^ |
| 7 | Kapolobwe, Likasi, Kikula, Kambove, Panda, Kowe, Vangu, Kipushi, Ruashi, Kanzenze, Tshamilemba, Mubunda, Lukafu, Kisanga, Kamalondo, Bunkeya, Katuba, Fungurume, Lubumbashi, Kapemba, Kenya | Week 2 | Week 17 | 96.31 | 962 | 335.57 | 1.0x10^-17^ |
| 8 | Ubundu, Yaleko, Lubunga, Makiso Kisangani, Mangobo, Lowa, Tshopo, Wanie Rukula, Kabondo, Opala | Week 25 | Week 37 | 117.57 | 996 | 375.13 | 1.0x10^-17^ |
| 9 | Nyemba, Kalemie | Week 27 | Week 31 | 87.81 | 965 | 388.47 | 1.0x10^-17^ |
| 10 | Kailo, Kindu, Alunguli | Week 38 | Week 52 | 58.93 | 536 | 149.61 | 1.0x10^-17^ |
| 11 | Mbanza Ngungu, Gombe Matadi, Boko Kivulu, Kwilu Ngongo, Kisantu | Week 41 | Week 46 | 52.88 | 228 | 26.12 | 1.0x10^-17^ |
| 12 | Fataki, Rimba, Linga, Drodro, Lita, Mangala, Rethy, Jiba, Logo, Kambala, Mahagi, Bunia, Angumu, Bambu, Aungba, Nizi, Nyarambe, Nyakunde, Mongbwalu, Tchomia, Kilo | Week 1 | Week 8 | 76.98 | 541 | 166.38 | 1.0x10^-17^ |
| 13 | Binga, Mbaya, Bulu, Ndage, Budjala, Djombo, Bosomondanda, Pimu, Lisala, Bangabola, Bosomanzi | Week 29 | Week 39 | 113.60 | 822 | 343.49 | 1.0x10^-17^ |
| 14 | Iboko, Pendjwa, Ingende | Week 49 | Week 52 | 63.53 | 103 | 4.01 | 1.0x10^-17^ |
| 15 | Pweto, Kilwa, Kasimba, Kiambi | Week 39 | Week 49 | 120.85 | 304 | 64.34 | 1.0x10^-17^ |
| 16 | Moanda, Kitona, Boma Bungu, Lukula, Boma | Week 50 | Week 52 | 62.80 | 74 | 1.97 | 1.0x10^-17^ |
| 17 | Ruzizi | Week 2 | Week 6 | 0 | 143 | 14.95 | 1.0x10^-17^ |
| 18 | Bili | Week 22 | Week 34 | 0 | 401 | 125.51 | 1.0x10^-17^ |
| 19 | Moba | Week 1 | Week 11 | 0 | 419 | 141.83 | 1.0x10^-17^ |
| 20 | Wapinda, Monga, Likati, Bondo, Yakoma, Wasolo, Abuzi, Yambuku, Yamongili | Week 50 | Week 52 | 122.66 | 79 | 3.18 | 1.0x10^-17^ |
| 21 | Oicha, Beni, Kamango, Kalunguta, Komanda, Mutwanga, Mabalako, Boga, Vohovi, Gethy, Katwa, Butembo, Kyondo | Week 24 | Week 27 | 72.51 | 105 | 12.33 | 1.0x10^-17^ |
| 22 | Lukolela, Tandembele, Bandjau, Irebu, Ntondo, Yumbi, Mushie | Week 17 | Week 35 | 121.56 | 306 | 139.69 | 1.0x10^-17^ |
| 23 | Monika, Basankusu, Boende, Bolomba | Week 31 | Week 31 | 91.8 | 28 | 0.80 | 1.0x10^-17^ |
| 24 | Dilala, Mutshatsha, Kilela Balanda, Lualaba, Kinda, Lubudi | Week 1 | Week 5 | 119.73 | 28 | 3.67 | 7.5x10^-13^ |
| 25 | Bokoro, Bosobe | Week 39 | Week 41 | 75.08 | 18 | 1.33 | 1.1x10^-11^ |
| 26 | Kwamouth, Bandundu, Kikongo, Bolobo, Maluku I | Week 35 | Week 40 | 111.29 | 47 | 14.96 | 1.5x10^-07^ |
| 27 | Watsa, Makoro, Damasi, Gombari, Biringi | Week 23 | Week 23 | 75.18 | 4 | 0.054 | 0.0022 |
