# Supplementary material for: Modalities and preferred routes of geographic spread of cholera from endemic areas in eastern Democratic Republic of the Congo
Source: PLoS One. 2022 Feb 7;17(2):e0263160. doi: 10.1371/journal.pone.0263160 (PMC8820636; doi:10.1371/journal.pone.0263160)
Supplement: S18 Table — (DOCX) [file pone.0263160.s021.docx]

**S18 Table.** Spatiotemporal clusters of cholera cases, DRC, 2017.

| **Cluster number** | **Health zones** | **Start time** | **End time** | **Radius (km)** | **Observed cases** | **Expected cases** | ***p*** |
| --- | --- | --- | --- | --- | --- | --- | --- |
| 1 | Kirotshe, Minova, Nyiragongo, Goma, Karisimbi | Week 27 | Week 37 | 38.45 | 8170 | 3539.59 | 1.0x10^-17^ |
| 2 | Bibanga, Tshitenge, Lukelenge, Muya, Dibindi, Tshilenge, Lubilanji, Bonzola, Kansele, Diulu, Bimpemba, Nzaba, Mpokolo, Mukumbi, Mulumba, Tshitshimbi, Kabinda, Kasansa, Ngandajika, Miabi, Tshilundu, Lubunga, Lukashi lualu, Kabeya Kamuanga, Kanda Kanda, Kalambayi Kabanga | Week 44 | Week 50 | 85.88 | 2186 | 474.97 | 1.0x10^-17^ |
| 3 | Yamongili, Bumba, Yambuku, Lolo, Yamaluka, Bosondjo, Abuzi, Yalimbongo, Aketi | Week 1 | Week 10 | 113.17 | 1382 | 192.88 | 1.0x10^-17^ |
| 4 | Mangembo, Luozi, Gombe Matadi, Kibunzi, Mbanza Ngungu, Kimpese, Massa, Mont Ngafula II, Kwilu Ngongo, Binza Ozone, Binza Météo, Sona Pangu, Mont Ngafula I, Selembao, Kintambo, Boko Kivulu, Bandalungwa, Bumbu, Kokolo, Ngiri Ngiri, Makala, Kasa Vubu, Lingwala, Kinkonzi, Police, Sona Bata, Lemba, Kalamu I, Ngaba, Kinshasa, Gombe, Kalamu II, Kisenso, Barumbu, Limeté | Week 17 | Week 27 | 123.45 | 1684 | 371.27 | 1.0x10^-17^ |
| 5 | Mambasa, Niania, Lolwa, Mandima | Week 8 | Week 11 | 91.82 | 574 | 33.42 | 1.0x10^-17^ |
| 6 | Pinga, Kayna, Kibua, Alimbongo, Musienene, Mweso, Kibirizi, Masisi, Walikale | Week 39 | Week 52 | 91.89 | 3587 | 1632.84 | 1.0x10^-17^ |
| 7 | Kabalo, Ankoro | Week 1 | Week 5 | 79.68 | 645 | 79.08 | 1.0x10^-17^ |
| 8 | Yumbi, Bolobo, Tandembele, Mushie, Lukolela | Week 8 | Week 18 | 97.56 | 720 | 105.98 | 1.0x10^-17^ |
| 9 | Pangi, Salamabila, Kalima, Shabunda, Alunguli, Kindu, Kunda, Kasongo, Kakole, Kailo | Week 1 | Week 8 | 120.69 | 770 | 138.94 | 1.0x10^-17^ |
| 10 | Kakenge, Bulape, Bena Leke, Mwetshi, Kole, Mweka, Mushenge, Bena Dibele | Week 45 | Week 51 | 104.51 | 827 | 166.79 | 1.0x10^-17^ |
| 11 | Mampoko, Lotumbe, Mankanza, Wangata, Bomongo, Bolenge, Lilanga Bobangi, Mbandaka | Week 6 | Week 18 | 105.13 | 640 | 100.52 | 1.0x10^-17^ |
| 12 | Kimbi Lulenge | Week 12 | Week 12 | 0 | 221 | 6.07 | 1.0x10^-17^ |
| 13 | Mwenga, Mwana, Mubumbano, Kaziba, Kamituga, Kaniola, Itombwe, Haut Plateau, Walungu, Nyangezi, Lemera, Nyatende, Bagira Kasha, Kitutu, Ibanda, Bagira Kasha, Kalonge, Ruzizi, Kabare, Uvira, Mulungu, Nundu, Miti Murhesa, Minembwe, Kahele, Bunyakiri, Idjwi, Katana | Week 35 | Week 47 | 114.38 | 5046 | 3199.72 | 1.0x10^-17^ |
| 14 | Kalemie, Kasimba, Kilwa, Moba | Week 1 | Week 19 | 101.09 | 1251 | 474.68 | 1.0x10^-17^ |
| 15 | Kinkondja | Week 21 | Week 25 | 0 | 298 | 35.28 | 1.0x10^-17^ |
| 16 | Lubudi, Fungurume, Kilela Balanda, Bukama | Week 48 | Week 52 | 83.66 | 381 | 74.87 | 1.0x10^-17^ |
| 17 | Binga, Mbaya, Bulu, Ndage, Budjala, Djombo, Bosomondanda, Pimu, Lisala, Bangabola, Bosomanzi | Week 4 | Week 12 | 113.60 | 276 | 37.71 | 1.0x10^-17^ |
| 18 | Mulongo, Mukanga, Manono, Lwamba, Malemba Nkulu | Week 34 | Week 43 | 96.42 | 2158 | 1232.69 | 1.0x10^-17^ |
| 19 | Yabahondo, Isangi, Basoko, Yahisule, Yakusu, Yahuma, Yaleko, Basali | Week 1 | Week 20 | 124.06 | 360 | 78.24 | 1.0x10^-17^ |
| 20 | Boma, Boma Bungu, Nzanza, Matadi, Lukula, Inga, Moanda | Week 1 | Week 15 | 62.80 | 387 | 94.58 | 1.0x10^-17^ |
| 21 | Lusangi, Kampene, Kongolo | Week 37 | Week 43 | 63.79 | 548 | 184.09 | 1.0x10^-17^ |
| 22 | Nyarambe | Week 20 | Week 25 | 0 | 160 | 15.43 | 1.0x10^-17^ |
| 23 | Mufunga Sampwe, Bunkeya, Mitwaba, Butumba | Week 26 | Week 31 | 110.22 | 184 | 26.36 | 1.0x10^-17^ |
| 24 | Dekese | Week 47 | Week 52 | 0 | 187 | 27.73 | 1.0x10^-17^ |
| 25 | Sia, Djuma, Mokala, Vanga, Bosobe | Week 30 | Week 36 | 76.37 | 270 | 71.87 | 1.0x10^-17^ |
| 26 | Tchomia, Bunia, Nizi, Gethy, Rwampara, Lita, Boga, Bambu, Drodro, Fataki | Week 42 | Week 49 | 73.18 | 268 | 74.72 | 1.0x10^-17^ |
| 27 | Koshibanda, Ipamu, Idiofa, Mikope, Kimputu, Mukedi, Ilebo, Banga Lubaka, Mungindu, Oshwe | Week 43 | Week 49 | 114.25 | 282 | 93.76 | 1.0x10^-17^ |
| 28 | Lowa, Ubundu | Week 24 | Week 27 | 105.83 | 50 | 2.53 | 1.0x10^-17^ |
| 29 | Wanie Rukula, Makiso Kisangani, Kabondo, Lubunga, Tshopo | Week 7 | Week 23 | 106.17 | 106 | 20.67 | 1.0x10^-17^ |
| 30 | Adi, Laybo, Ariwara, Adia, Aba, Aru, Makoro, Biringi, Aungba, Faradje, Mahagi, Kambala, Rimba, Logo | Week 4 | Week 7 | 124.64 | 43 | 2.17 | 1.0x10^-17^ |
| 31 | Kenya, Katuba, Kamalondo, Kapemba, Kisanga, Mubunda, Tshamilemba, Lubumbashi | Week 39 | Week 42 | 33.11 | 72 | 14.65 | 1.0x10^-17^ |
| 32 | Kikongo, Kenge, Bagata, Yanga bosa, Kwamouth, Boko, Bandundu | Week 24 | Week 34 | 96.54 | 103 | 29.79 | 1.0x10^-17^ |
| 33 | Kimvula, Nselo, Ngidinga, Popokabaka, Kisantu | Week 31 | Week 32 | 91.75 | 30 | 4.03 | 1.8x10^-12^ |
| 34 | Kilwa | Week 1 | Week 3 | 0 | 14 | 1.12 | 3.8x10^-07^ |
| 35 | Opala | Week 18 | Week 19 | 0 | 7 | 0.11 | 4.6x10^-07^ |
| 36 | Inongo, Kiri, Pendjwa, Bokoro | Week 26 | Week 30 | 94.09 | 23 | 4.02 | 1.5x10^-06^ |
| 37 | Songa, Kamina Base, Kamina, Kabongo, Kayamba, Kabondo Dianda | Week 45 | Week 51 | 104.14 | 51 | 17.75 | 2.7x10^-06^ |
| 38 | Beni, Kalunguta, Oicha, Mabalako, Vohovi, Mutwanga | Week 3 | Week 16 | 35.88 | 26 | 5.91 | 2.6x10^-05^ |
| 39 | Opienge | Week 42 | Week 43 | 0 | 10 | 0.70 | 8.3x10^-05^ |
| 40 | Tumba, Wembo Nyama, Kibombo | Week 31 | Week 31 | 57.44 | 9 | 0.54 | 0.0001 |
| 41 | Befale, Bongandanga | Week 16 | Week 16 | 89.20 | 4 | 0.029 | 0.0004 |
